# Nanomaterials‐Induced Redox Imbalance: Challenged and Opportunities for Nanomaterials in Cancer Therapy

**DOI:** 10.1002/advs.202308632

**Published:** 2024-02-21

**Authors:** Xumeng Wu, Ziqi Zhou, Kai Li, Shaoqin Liu

**Affiliations:** ^1^ School of Life Science and Technology Harbin Institute of Technology Harbin 150006 China; ^2^ Zhengzhou Research Institute Harbin Institute of Technology Zhengzhou 450046 China; ^3^ School of Medicine and Health Harbin Institute of Technology Harbin 150006 China

**Keywords:** reactive oxygen species (ROS), redox imbalance, reductive stress, stimuli‐responsive nanomaterials

## Abstract

Cancer cells typically display redox imbalance compared with normal cells due to increased metabolic rate, accumulated mitochondrial dysfunction, elevated cell signaling, and accelerated peroxisomal activities. This redox imbalance may regulate gene expression, alter protein stability, and modulate existing cellular programs, resulting in inefficient treatment modalities. Therapeutic strategies targeting intra‐ or extracellular redox states of cancer cells at varying state of progression may trigger programmed cell death if exceeded a certain threshold, enabling therapeutic selectivity and overcoming cancer resistance to radiotherapy and chemotherapy. Nanotechnology provides new opportunities for modulating redox state in cancer cells due to their excellent designability and high reactivity. Various nanomaterials are widely researched to enhance highly reactive substances (free radicals) production, disrupt the endogenous antioxidant defense systems, or both. Here, the physiological features of redox imbalance in cancer cells are described and the challenges in modulating redox state in cancer cells are illustrated. Then, nanomaterials that regulate redox imbalance are classified and elaborated upon based on their ability to target redox regulations. Finally, the future perspectives in this field are proposed. It is hoped this review provides guidance for the design of nanomaterials‐based approaches involving modulating intra‐ or extracellular redox states for cancer therapy, especially for cancers resistant to radiotherapy or chemotherapy, etc.

## Graphical abstract

Cancer cells display redox imbalance compared to normal cells, thus targeting these distinctive biochemical attributes provides new opportunities for cancer treatment. This work focuses on recent advancements in the design of stimuli‐responsive nanomaterials that selectively regulate redox balance in cancer cells over the threshold for activating cancer cell death.

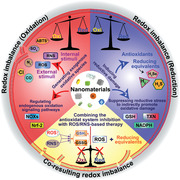

## 1 Introduction

Cancer remains a leading cause of death worldwide.^[^ ^1^ ^]^ Despite the advancements in cancer treatment modalities, the genetic heterogeneity, diversity, and complexity of tumors pose daunting challenges for developing more efficient and effective cancer therapies.^[^ ^2^ ^]^ Abundant data revealed that cancer cells experience oxidative stress during certain critical phases of their evolutionary and progressive stages.^[^ ^3^ ^]^ Mechanisms leading to oxidative stress in cancer cells may involve hyperactivation of anabolic pathways,^[^ ^4^ ^]^ increased mitochondrial function,^[^ ^5^ ^]^ malfunction of the electron transport chain (ETC) as a result of mitochondrial DNA mutations,^[^ ^6^ ^]^ and oncogenic pathway activation.^[^ ^7^ ^]^ Consequently, cancer cells exhibit redox imbalance compared to normal cells and deploy a variety of antioxidant mechanisms to counterbalance the oxidative damage resulting from oxidative stress. These mechanisms include glutathione (GSH),^[^ ^8^ ^]^ thioredoxin (Trx, aka TXN),^[^ ^9^ ^]^ antioxidant enzymes (e.g., glutathione peroxidases (GPXs),^[^ ^10^ ^]^ catalases (CATs),^[^ ^11^ ^]^ and superoxide dismutases (SODs),^[^ ^12^ ^]^ and their transcriptional regulators, such as the nuclear factor erythroid 2‐related factor 2 (Nrf‐2)^[^ ^13^ ^]^ and BTB domain and CNC homolog 1 (BACH1).^[^ ^14^ ^]^ Therefore, employing redox‐modulating strategies to specifically target these distinctive biochemical attributes of cancer cells presents a feasible therapeutic approach for cancer treatment. Indeed, various cancer treatment methods involving the regulation of redox levels have been widely reported in recent years. They are basically divided into three main categories: 1) enhancing the metabolic capacity of the antioxidants and antioxidation enzymes to suppress the tumor growth; 2) inducing excess highly reactive substances in cancerous cells to exceed the safe threshold and leading to the demise of these malignant cells; 3) disabling the cellular antioxidation systems in the tumor itself to prevent the clearance of reactive substances in cells.^[^ ^15^ ^]^ However, it has been frequently observed in clinical trials that employing antioxidants as cancer therapies can actually stimulate the emergence of additional phenotypes in malignant tumors, and tend to increase cancer incidence and cancer‐related deaths.^[^ ^16^ ^]^ Many natural or synthetic ROS‐inducers have been found to promote the generation of ROS by affecting the ETC, redox cycling compounds, and disrupting the antioxidative defense mechanisms. Most chemotherapeutic drugs, such as doxorubicin (Dox), cisplatin, 5‐fluorouracil (5‐FU), and arsenic trioxide (ATO), can kill cancer cells by directly or indirectly facilitating ROS accumulation.^[^ ^17^ ^]^ Nonetheless, the broader application of these agents in cancer therapy is substantially hindered by their lack of specificity and the associated systemic toxicity akin to the limitations encountered with traditional chemotherapy and radiotherapy regimens.^[^ ^18^ ^]^

The ability of nanomaterials to respond actively to local microenvironments and enable precise spatial and temporal functions confers diverse advantages for their utilization in cancer therapy. A series of stimuli‐responsive nanomaterials has been meticulously engineered and applied for the treatment of cancer. These materials are designed to either directly boost the production of ROS or disrupt antioxidative defense mechanisms, potentially resulting in cell death via apoptosis, autophagy, ferroptosis, or necrosis.^[^ ^19^ ^]^ Given this rapidly increasing interest in using nanomaterials for modulating redox imbalance in cancer cells, it is imperative to provide a comprehensive overview of the diverse stimuli‐responsive nanoplatforms available for regulating redox equilibrium within cancer cells. While numerous excellent reviews have described the realm of nanomaterials pertaining to oxidative stress,^[^ ^20^ ^]^ there is a lack of reports addressing the design of nanomaterials tailored to enhance ROS/reactive nitrogen species (RNS) production, disrupt endogenous antioxidant defense systems, or manage reduction imbalances, either individually or in combination. Here, we first delineate the physiological features of redox imbalance in cancer cells and highlight the challenges in modulating redox state in cancer cells. Then, we systematically categorize and expound upon nanomaterials designed to regulate redox imbalance according to their ability to target redox adaptations in cancer cells, particularly focusing on stimuli‐responsive nanomaterials. Finally, we offer insights into the future prospects of this field. We hope this review provides comprehensive information on different methodologies that bear an outstanding potential to further promote this very promising field from basic theory to clinical application.

## 2 Redox Imbalance and Biochemical Changes in Cancer

**Figure 1:**
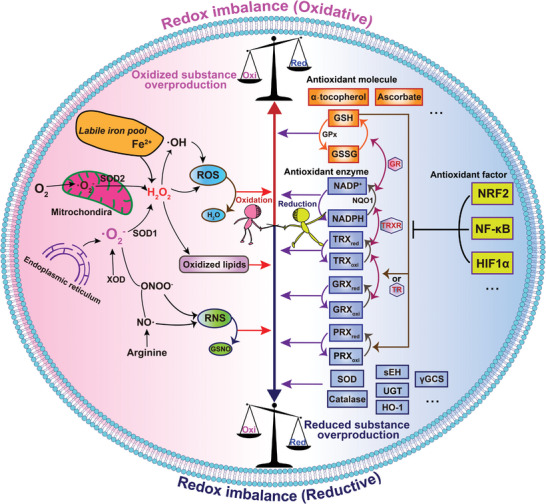
Schematic illustration of abnormal imbalance in the redox systems of tumor cells.

Oxidation/reduction (redox) homeostasis is an essential and dynamic process that allows proper cellular reactions and regulates biological responses.^[^ ^21^ ^]^ It is maintained by the net physiologic balance between the production of reactive oxidative substances containing ROS,^[^ ^22^ ^]^ RNS^[^ ^23^ ^]^ and their secondary products^[^ ^24^ ^]^ and the antioxidant defense system consisting of smaller antioxidant molecules, antioxidant enzymes (such as glutathione‐S‐transferases (GSTs), nicotinamide adenine dinucleotide phosphate (NADP(H)), NAD(P)H quinone dehydrogenase 1 (NQO1), GPXs, CATs, SODs, epoxide hydrolases (EHs), heme oxygenase‐1 (HO‐1), UDP‐glucuronosyl transferases (UGTs), gamma‐glutamylcysteine synthetases (γ‐GCSs), and others), as well as redox cofactors. The cellular redox state is regulated by the thioredoxin/thioredoxin reductases (Trx/TrxRs), peroxiredoxin/peroxiredoxin reductases (Prx/PrxRs), and glutaredoxins/glutaredoxins reductases (Grx/GrxRs) systems, which modify specific redox‐sensitive proteins, thereby triggering related signaling events. The Nrf‐2, nuclear factor kappa‐B (NF‐κB), and hypoxia‐inducible factor 1‐alpha (HIF‐1α) system may then be directly or indirectly redox‐regulated, leading to an antioxidant response (**Figure** **1**).

Unlike normal cells, hyperproliferative cancer cells have greater demands for the energy required to promote macromolecular biosynthesis and the generation of the essential precursors of amino acids, nucleotides, and lipids.^[^ ^25^ ^]^ Moreover, cancer cells must undergo metabolic rewiring to contend with the often‐decreased availability of nutrients, increased acidity, and onset of hypoxic conditions prevalent in the densely populated and expanding tumor microenvironment (TME).^[^ ^26^ ^]^ In response to these demands, cancer cells undergo metabolic reprogramming, leading to alterations in antioxidant‐to‐oxidant homeostasis. Although a comprehensive assessment of the redox status across various cell types and different phases is currently lacking,^[^ ^27^ ^]^ most cancer cells exhibit elevated levels of ROS^[^ ^28^ ^]^/RNS^[^ ^29^ ^]^ in the early phase of mitophagy. For example, when compared to normal tissue, where the H_2_O_2_ levels typically range ≈0.5 × 10^−3^ µmol 10^−4^ cells,^[^ ^30^ ^]^ the concentration of H_2_O_2_ in tumors can be as high as 100 µm.^[^ ^31^ ^]^ To evade senescence, apoptosis, necrosis, and ferroptosis, cancer cells strategically modulate multiple antioxidant defense mechanisms for the proper elimination of increased levels of ROS/RNS to establish new redox homeostasis.^[^ ^32^ ^]^ For example, increased expression or activity of antioxidative‐related enzymes such as SOD and its mimics, CATs, GPXs, and Prx have been demonstrated in many different cancers. The level of thiol‐based antioxidants GSH, Trx, and Prx is also upregulated to scavenge the excessive ROS.^[^ ^9^, ^33^ ^]^ In addition, the appropriate levels of ROS in the short‐to‐medium time span will trigger the upregulation of antioxidant transcription factors, such as HIF‐1α or Nrf‐2, to support cancer cell proliferation by genetic reprogramming^[^ ^33^, ^34^ ^]^ (Figure 1). However, redox alterations in cancer cells are very complex. For example, cancer stem cells and some drug‐resistant cancer cells maintain ROS levels to low amounts, which is mainly due to the expression of ROS scavenging molecules and their efficient DNA repair systems, thereby promoting resistance to oxidative targeted therapies. Cancer cells maintain this “tense and fragile balance” by counteracting the elevated ROS level with the alteration of redox signaling pathways responsible for increased antioxidant synthesis to promote the rapid proliferation, invasiveness, metastasis, and other malignant phenotypes of tumor cells.^[^ ^35^ ^]^ For more extensive exploration of redox homeostasis features, readers are referred to other comprehensive reviews.^[^ ^27^, ^34^, ^36^ ^]^

## 3 Nanomaterials that Manipulating Redox Balance in Cancer Cells for Cancer Therapy

**Figure 2:**
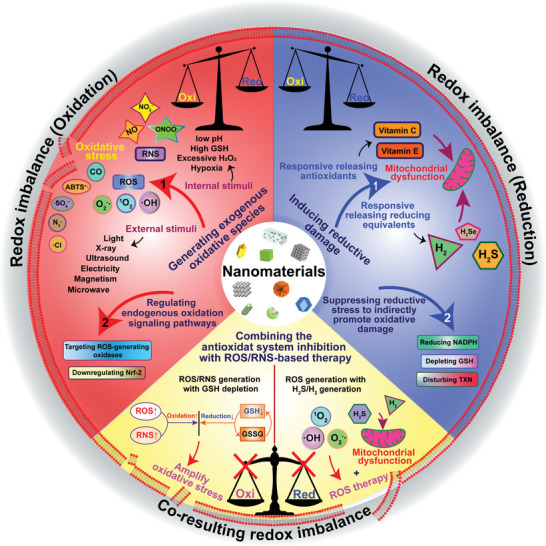
Classification of the nanomaterials that induce redox imbalance.

As outlined in the preceding sections, the dysregulated redox systems within cancer cells significantly contribute to processes like proliferation, survival, and metastasis in various cancer types. Consequently, perturbing the delicate tumor redox balance within tumors serves as a promising anti‐tumor approach with tumor selectivity. Therapeutic approaches either promoting reactive oxidative substances production or disrupting the antioxidant defense mechanisms in cancer cells could result in a dramatic increase in the generation of reactive oxidative substances to the point where they trigger cell death. Various drugs that manipulate redox equilibrium have achieved promising outcomes in killing cancer cells, however, the adverse effects and therapy resistance induced by reactive oxidative substances limit their therapeutic potential. The designable, multi‐functional, and modifiable properties of nanomaterials provide many opportunities to manipulate redox imbalance in cancer cells for cancer therapy and enhance the antitumor effects of pro‐oxidants. In the past few years, numerous responsive nanomaterials could manipulate redox imbalance in cancer cells for cancer therapy through three kinds of action mechanisms: promoting the generation of highly reactive substances, disrupting the antioxidative defense mechanisms, or increasing the production of both. In this section, we summarized the nanomaterials that manipulate redox balance in cancer cells for therapeutic purposes, with a particular focus on stimuli‐responsive nanomaterials. Based on these mechanisms of action, these responsive nanomaterials are classified into three main categories: 1) inducing excessive reactive oxidative substances under internal stimuli or external stimuli beyond the toxic threshold to induce cell death and tumor destruction; 2) elevating reducing agents in cells to disturb redox balance; 3) combining the inhibition of the antioxidant mechanisms with boosting ROS/RNS production (**Figure** **2**).

### 3.1 Responsive Nanomaterials That Induce Excessive Reactive Oxidative Substances

**Figure 3:**
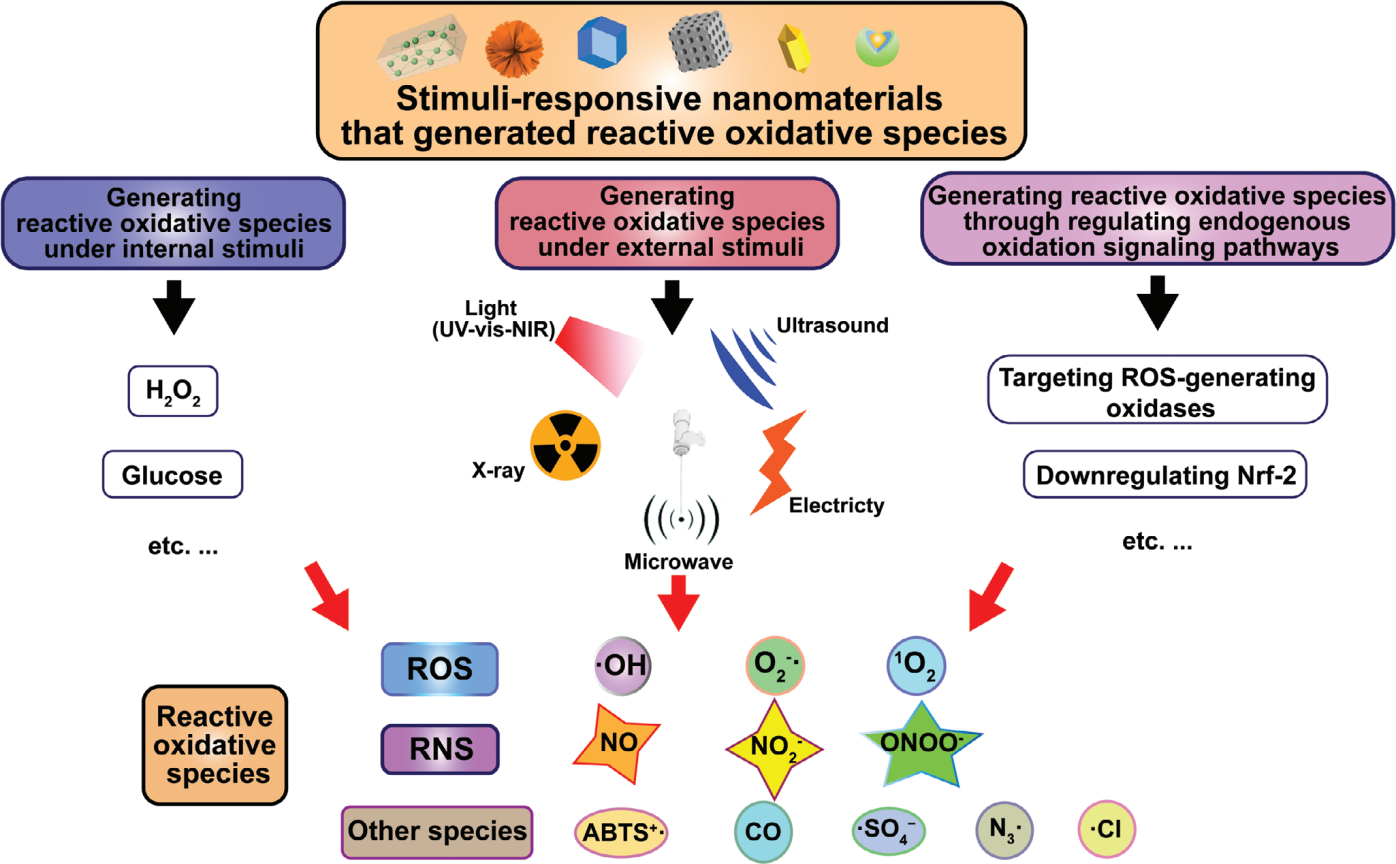
The category of stimuli‐responsive nanomaterials induces the generation of excessive reactive oxidative species.

Living species are continuously subjected to more than 20 kinds of chemically reactive species containing ROS, RNS, and their secondary products that are produced by internal sources (mitochondria, xanthine oxidase, phagocytes, reactions involving iron and other transition metals, peroxisomes, arachidonate pathways, exercise, ischemia/reperfusion, inflammation), external sources (environmental pollutants, tobacco smoke, radiations, certain drugs, etc.) and physiological factors (mental status and disease conditions).^[^ ^37^ ^]^ Examples of ROS, RNS, and their secondary products include hydroxyl radical (∙OH), hydrogen peroxide (H_2_O_2_), superoxide (O_2_∙^−^), peroxyl (ROO∙), and alkoxyl radicals (RO∙), hypochlorous acid (HClO), organic peroxides (ROOH), aldehydes (HCOR), nitric oxide (NO∙), nitrous oxide (N_2_O), peroxynitrite (ONOO^−^) and its protonated form (ONOOH), nitrogen dioxide (∙NO_2_), nitrosothiols, etc.^[^ ^22^ ^]^ Below a certain reactive oxidative species threshold, these molecules play a crucial role in the regulation of several cellular processes such as cell signaling, proliferation, differentiation, and cell death. However, the unpaired electrons of reactive oxidative species allow them to grab electrons from the surrounding substances indiscriminately and induce non‐specific damage to proteins, lipids, and DNA, resulting in senescence, degeneration, or fatal lesions in cells.^[^ ^38^ ^]^ Therefore, above the reactive oxidative species threshold, reactive oxidative species can trigger apoptotic signals.^[^ ^39^ ^]^ It is now known that many metabolically transformed and fast‐growing cancer cells have higher reactive oxidative species levels than neighboring normal cells. This provides a possible therapeutic window to eradicate cancer cells rather than normal cells by stimulating endogenous reactive oxidative species generation in tumor cells. Various nanomaterials and strategies have been explored for inducing excessive reactive oxidative species generation beyond the toxic threshold to induce cell death and tumor destruction. They are basically involved in three main categories: 1) inducing the generation of highly reactive oxidative species directly in tumor cells under internal stimuli, such as H_2_O_2_ and glucose; 2) inducing the generation of highly reactive oxidative species by external stimuli, such as light, ultrasound (US), X‐ray irradiation, etc.; 3) inducing the generation of highly reactive oxidative species through regulating endogenous oxidation signaling pathways (**Figure** **3**).

#### 3.1.1 TME‐Responsive Nanomaterials That Generating Reactive Oxidative Species Under Internal Stimuli

The abnormal metabolism and metabolite accumulation in cancer cells lead to the development of a unique TME during their evolution. The TME typically exhibits a low pH, high GSH concentration, excessive H_2_O_2_, severe hypoxia, etc.^[^ ^40^ ^]^ These characteristic features provide numerous potential targets for the development of internal stimuli‐responsive nanomaterials designed to induce excessive reactive oxidative species. These reactive oxidative species encompass ROS (·OH, ^1^O_2_, ·O_2_^−^), RNS (NO, ONOO^−^), and other reactive oxidative species (carbon‐centered radicals like CO and alkyl radicals, sulfate radicals). **Table** **1** summarizes the redox capacity, half‐life, pH tolerance, selectivity, chemical stability, and antitumor mechanism of these active species used for cancer therapy.

**Table 1:** Comparison of different reactive oxidative species.

Reactive oxidative species	Redox capacity [V vs NHE]	Half‐life	pH tolerance	Selectivity	Chemical stability	Therapeutic mechanism in tumor
·OH	2.7–2.8 V^[^ ^86^ ^]^	≈10^−9 ^s^[^ ^87^ ^]^	Wide^b^)	None^[^ ^88^ ^]^	Unstable^[^ ^89^ ^]^	The high oxidative capacity of ·OH causes chain oxidation reactions that lead to oxidation of the surrounding lipids, proteins, and DNA, thereby resulting in damage to the cell^[^ ^90^ ^]^
^1^O_2_	2.2 V^[^ ^91^ ^]^	≈10^−6 ^s^[^ ^92^ ^]^	3–11^[^ ^93^ ^]^	Higher affinity for unsaturated carbon or amine groups^[^ ^94^ ^]^	Strong versatile inorganic ions resistance^[^ ^94^ ^]^	^1^O_2_ quickly oxidizes the sulfur‐containing or aromatic amino acids in proteins and also causes strong damage to lipid membranes^[^ ^95^ ^]^
·O_2_^−^	0.31 V^[^ ^96^ ^]^	ms–hours^a^) ^[^ ^97^ ^]^	Wide^[^ ^98^ ^]^	Attacking the positively charged components of any organic species in the absence of protons^[^ ^98^ ^]^	Unstable^[^ ^98^ ^]^	·O_2_^−^ react with O_2_ and NO to generate H_2_O_2_ and ^−^ONOO, causing lipid peroxidation damage and mitochondrial dysfunction^[^ ^99^ ^]^
NO·	1.2 V^[^ ^100^ ^]^	0.09–2 s in vivo^[^ ^101^ ^]^	Wide^[^ ^102^ ^]^	Readily reacting with metal complexes, hypervalent complexes, and hemoglobin^[^ ^102^ ^]^	Stable for biomolecules whose orbitals are completely filled^[^ ^102^ ^]^	Lipid peroxidation and nitrosative stress triggered by NO· lead to DNA and plasma membrane damage, resulting in tumor apoptosis^[^ ^103^ ^]^
ONOO^−^	1.4 V^[^ ^104^ ^]^	≈10 ms in vivo^[^ ^105^ ^]^	Highly depending on pH^[^ ^105b^ ^]^	Rapidly reacting with CO_2_ and biomolecules.^[^ ^105b^ ^]^	Unstable^[^ ^106^ ^]^	ONOO^−^ mediates protein oxidation and nitration, lipid peroxidation, and mitochondrial dysfunction, ultimately leading to cancer cell death^[^ ^107^ ^]^
NO_2_^−^	1.03 V^[^ ^108^ ^]^	110 s^[^ ^109^ ^]^	–	Rapidly reacting with organic compounds such as biological lipid membrane^[^ ^110^ ^]^ or hemoglobin,^[^ ^109^ ^]^ and exhibits moderate reactivity with phenoxide ions, anilines, phenothiazines, thiols, and ascorbate^[^ ^108a^ ^]^	Unstable^[^ ^111^ ^]^	NO_2_^−^ induces an increase in intracellular calcium ions and ROS/RNS levels, leading to endoplasmic reticulum stress and mitochondrial dysfunction^[^ ^112^ ^]^
·SO_4_^−^	2.60 V^[^ ^113^ ^]^	≈3–4 × 10^−5^ s^[^ ^114^ ^]^	2–9^[^ ^115^ ^]^	Selectively attacking specific chemical bonds^[^ ^116^ ^]^	Unstable^[^ ^117^ ^]^	The high oxidizing capacity and long diffusion distance of ·SO_4_^−^ can efficiently mediate oxidative stress in tumor^[^ ^118^ ^]^
·Cl	2.47 V^[^ ^119^ ^]^	–	–	Rapidly reacting with electron‐rich moieties^[^ ^119^, ^120^ ^]^	Unstable^[^ ^121^ ^]^	**·**Cl causes one‐electron oxidation with DNA skeletons to inflict severe cellular damage directly^[^ ^122^ ^]^
HOCl	1.48 V^[^ ^123^ ^]^	1 × 10^−7^ s^[^ ^124^ ^]^	3–6^[^ ^125^ ^]^	Rapidly reacting with the sulfur‐containing amino acid side‐chains of Met and Cys^[^ ^126^ ^]^	Relatively stable^[^ ^127^ ^]^	HOCl selectively meditates malignant cells to apoptosis^[^ ^128^ ^]^
ABTS∙^+^	0.68 V^[^ ^129^ ^]^	8 × 10^−3^ s	5–9^[^ ^130^ ^]^	Almost none^[^ ^131^ ^]^	Stable in low temperatures (below 5 °C)^[^ ^132^ ^]^	The thermal‐labile azo initiator in the tumor can decompose under heating or US to generate two ABTS∙^+^ to achieve oxygen‐independent tumor killing^[^ ^133^ ^]^
·CF_3_	−0.58 V^[^ ^134^ ^]^	Very short (<10^−10 ^s)^[^ ^134^ ^]^	4.8–9.6^[^ ^135^ ^]^	Reacting with 18 of the 20 common amino acids, which exhibit relative inertness toward ·OH^[^ ^136^ ^]^	Unstable^[^ ^134^ ^]^	·CF_3_ resulted in downregulation of ERCC1 in chemotherapy, which inhibit the repair of cisplatin‐damaged DNA^[^ ^137^ ^]^
N_3_∙	1.32 V^[^ ^138^ ^]^	<2 × 10^−8 ^s^[^ ^138^ ^]^	4–13^c^)	Selectively oxidizing aromatic amino acid residues in proteins^[^ ^138^ ^]^	Unstable^[^ ^138^ ^]^	The generated N_3_∙ radical in tumor cells can destroy lysosomal membrane structure without oxygen^[^ ^139^ ^]^

^a)^ It depends on the concentration of ·O_2_^−^;^[^ ^140^ ^]^

^b)^ Fenton reaction produces more OH at lower pH;

^c)^ N_3_∙ radical‐mediated electron transfer oxidation is more efficient in alkaline aqueous solutions than in acidic conditions.^[^ ^138^ ^]^

##### Generation of ·OH

**Figure 4:**
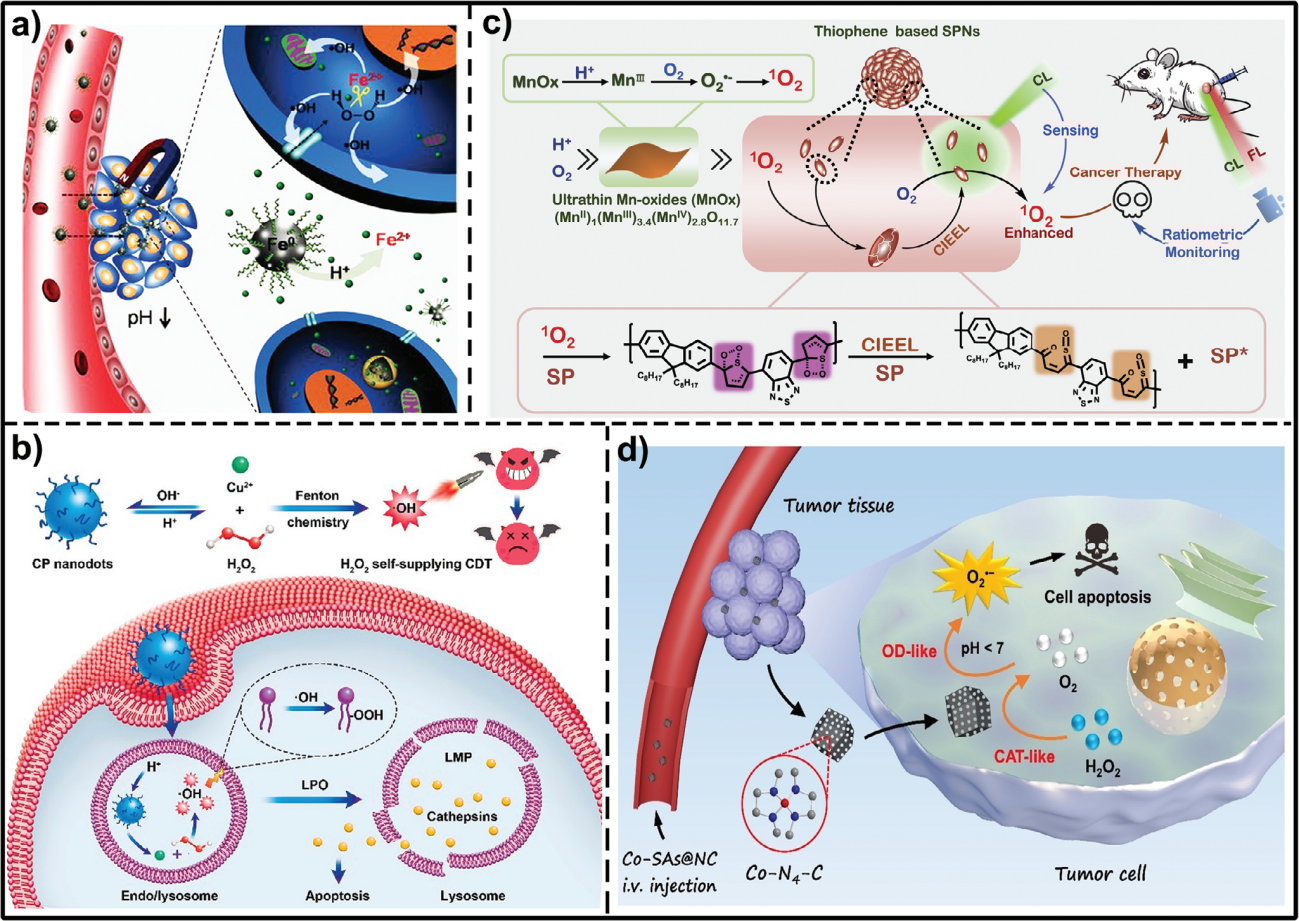
Schematic illustration of TME‐responsive nanomaterials that generate ROS under internal stimuli through Fenton or Fenton‐like reactions for cancer treatment. a) The application of AFeNPs in cancer therapy by localized Fenton reaction. Reproduced with permission.^[^ ^43^ ^]^ Copyright 2016, Wiley‐VCH. b) Copper peroxide nanodots release Cu^2+^ and H_2_O_2_ under acid TME to generate ·OH through a Fenton‐like reaction, thereby achieving H_2_O_2_ self‐supplying CDT process. Reproduced with permission.^[^ ^64^ ^]^ Copyright 2019, American Chemical Society. c) Chemodynamic and chemiluminescenct system based on ultrathin MnO_x_ nanosheet and semiconducting polymer nanoparticles. Reproduced with permission.^[^ ^77^ ^]^ Copyright 2020, Elsevier. d) Co single atom on N‐doped porous carbon (Co‐SAs@NC) as a bifunctional nanozyme triggered production of ·O_2_^−^ for synergistic tumor therapy. Reproduced with permission.^[^ ^85^ ^]^ Copyright 2022, Wiley‐VCH.

·OH, a highly reactive ROS, has a very high oxidation potential (2.7 eV) among radicals (Table 1). The prevailing design principle for generating ·OH involves Fenton or Fenton‐like reactions, typically based on transition metal elements with multiple valences.^[^ ^41^ ^]^ These transition metal elements with multiple valences play a significant role in TME manipulation. They facilitate the conversion of the overexpressed H_2_O_2_ into ·OH through an electron transfer process, thereby consuming the reducing GSH and ultimately achieving overall regulation of TME.^[^ ^42^ ^]^ Based on transition metal‐based Fenton/Fenton‐like reactions and the TME, Shi's group synthesized amorphous iron nanoparticles (AFeNPs) and used them as theragnostic agents (defined as chemodynamic therapy: CDT).^[^ ^43^ ^]^ Ionization of the AFeNPs enables on‐demand ferrous ion release in mild acidity of the tumor and subsequently promotes the disproportionation of overproduced H_2_O_2_ to produce ·OH at the tumor site (**Figure** **4**a). The lethal ·OH is capable of impairing DNA and converting polyunsaturated fatty acids to lipid peroxides, followed by the activation of a series of antitumorigenic pathways, thus inducing cell apoptosis/ferroptosis to achieve tumor treatment. Considering that Fenton/Fenton‐like reactions are difficult to trigger under alkaline conditions and that the tumor cell (from 100 µm to 1 mm)^[^ ^31^ ^]^ has a higher H_2_O_2_ concentration than the normal cell (≈20 nm),^[^ ^44^ ^]^ AFeNPs enables highly selective tumor treatment with minimal toxic side‐effects. Although the exact mechanism of Fe‐based‐Fenton therapy is far from fully understood due to the complicated TME and inevitable nanoparticle‐cell interactions,^[^ ^45^ ^]^ this does not hinder this method from emerging as a potent strategy for cancer treatment. Various Fe‐carrying nanomaterials such as iron oxide,^[^ ^46^ ^]^ Zero‐valent iron nanoparticles,^[^ ^47^ ^]^ iron chelated‐polymer,^[^ ^48^ ^]^ iron carbide,^[^ ^49^ ^]^ iron sulfide,^[^ ^50^ ^]^ ferrocene,^[^ ^51^ ^]^ iron‐engineered SiO_2_,^[^ ^52^ ^]^ single‐atom iron on MoS_2_,^[^ ^53^ ^]^ and iron‐organic complexes^[^ ^54^ ^]^ have been exploited to destroy cancer cells through Fe^2+^‐catalyzed ·OH generation (**Table** **2**). In addition to iron‐based nanomaterials, other metal ions, like copper (Cu), cobalt (Co), nickel (Ni), manganese (Mn), molybdenum (Mo), and titanium, also have similar reactivity to conventional Fenton reactions through Fenton‐like reactions, and the reaction efficiency is much higher than that of Fenton in the weakly acidic TME. Therefore, numerous Cu,^[^ ^55^ ^]^ Co,^[^ ^56^ ^]^ Ni,^[^ ^57^ ^]^ Mn,^[^ ^58^ ^]^ Mo,^[^ ^59^ ^]^ cerium (Ce),^[^ ^60^ ^]^ ruthenium (Ru),^[^ ^61^ ^]^ tungsten (W),^[^ ^62^ ^]^ and Ti^[^ ^63^ ^]^‐based nanomaterials have been engineered as Fenton‐like agents to generate ·OH. For example, Chen et al. synthesized copper peroxide nanodot as an activatable agent for enhanced CDT by self‐supplying H_2_O_2_. After endocytosis into tumor cells, the acidic environment of endo/lysosomes accelerated the dissociation of copper peroxide nanodot, enabling a release of abundant H_2_O_2_ in close proximity to an efficient Fenton catalyst^[^ ^64^ ^]^ (Figure 4b). Several excellent review papers have summarized these nanomaterials for cancer treatment.^[^ ^65^ ^]^

**Table 2:** Summary of the representative nanomaterials that generate ROS under internal stimuli.

Material	ROS type	ROS‐generating substance and therapy type	ROS producing mechanism	Comment	References
AFeNPs	∙OH	Fe^2+^	Fenton reaction^a)^	The first report of the nanomaterials to CDT.	[43]
MM@HMFe@BS	∙OH	Fe_2_O_3_	Fenton reaction	Hollow mesoporous iron oxide (HMFe) with high exposure of active atoms for enhanced ∙OH production.	[46a]
FDPC NCs	∙OH	Fe_3_O_4_	Fenton reaction	FDPC NCs achieve dynamic T2/T1‐switchable MRI and tri‐mode PTT‐chemo‐CDT of tumors under NIR irradiation.	[46b]
DMON@Fe^0^/AT	∙OH	Fe^0^ NPs	Fenton reaction	The ∙OH production by DMON@Fe^0^/AT attacks mitochondria and downregulates the expression of ferroportin 1, which disrupts the cellular iron metabolism system to kill 4T1 cells more efficiently than Fe^0^ nanoparticles.	[47c]
POEGMA‐b‐PTKDOPA	∙OH	Fe^3+^‐polymer	Fenton reaction	GO_x_ released from the nanomaterial generates H_2_O_2_ to catalyze Fe^3+^ to generate ∙OH, while the combination of CDT and ICB leads to an immune response to eliminate primary and distant tumors.	[48]
Fe_5_C_2_‐GOD@MnO_2_	∙OH	Fe_5_C_2_	Fenton reaction	The generated O_2_ and GOD (by decomposing MnO_2_ nanoshells) exhaust glucose in the tumor, thereby generating H_2_O_2_ which accelerates the Fenton reaction catalyzed by the Fe_5_C_2_.	[49]
ISNP	∙OH	FeSO_4_	Fenton reaction	Particle size reduction of FeSO_4_ from micron to nano size by hot‐melt extrusion enhanced the cellular uptake of ISNP, which exhibited enhanced antiproliferation and apoptosis potentials in colon adenocarcinoma cells.	[50a]
FeS_2_	∙OH	FeS_2_	Fenton reaction	Bioinformatics analysis guides ∙OH oxidation therapy.	[50b]
Fc@MSN‐F_DNA_/PTAD	∙OH	Fc	Fenton reaction	H_2_O_2_ permeates into the nanosphere and reacts with ferrocene to produce ·OH via Fenton reaction, which cleaves FDNA to detach ROX from PTAD, thus in turn, lights the ROX fluorescence (realizing in vivo fluorescence imaging of H_2_O_2_).	[51]
AMP NRs	∙OH	MIL‐100	Fenton reaction	AMP NRs simultaneously producing ·OH in response to the H_2_O_2_ of the TME and disrupting intracellular GSH endows the ability of ECDT.	[54a]
BSO/GA–Fe(II)	∙OH	Fe(II) complex	Fenton reaction	BSO/GA–Fe(II)@liposome is able to amplify intracellular oxidative stress via increasing ∙OH generation and reducing GSH biosynthesis.	[54b]
GOx@ZIF@MPN	∙OH	Fe(III)‐MPN	Fenton reaction	The tannic acid in the metal polyphenol network (MPN) reduced Fe(III) to Fe(II), which reacts with the self‐produced H_2_O_2_ (by GOx) to generate ∙OH for autocatalytic Fenton therapy.	[54c]
Cu_9_S_8_	∙OH	Cu^2+^	Fenton‐like reaction^b)^	Abundant defect sites improving the Fenton‐like catalytic efficiency of H_2_O_2_	[55a]
Copper peroxide	∙OH	Cu^2+^	Fenton‐like reaction	Copper peroxide easily dissociates in the acidic microenvironment of tumors.	[55c]
Mo_2_C POM	^1^O_2_	Mo^5+^	Fenton‐like reaction	The process of Mo^5+^ oxidation to Mo^6+^ led to a large amount of ^1^O_2_ production.	[59]
CMTN	^1^O_2_	MoO_4_^2−^	Fenton‐like reaction	The liposome vesicle co‐encapsulation system regulates pH 10–11, making MoO_4_^2−^ catalyze H_2_O_2_ to generate ^1^O_2_.	[76]
MnO_x_ nanosheets	^1^O_2_	MnO_x_	Fenton‐like reaction	Found the Mn center produces ^1^O_2_ first.	[77]
ClO@MOF/F68+Asc	^1^O_2_	NaClO+H_2_O_2_	Redox reaction	Ascorbate produces H_2_O_2_ that reacts with ClO^−^ to stoichiometrically produce ^1^O_2_.	[78]
Co‐SAs@NC	·O_2_^−^	Oxidase‐like nanozyme	CAT‐like enzyme to produce O_2_, then oxidase‐like enzyme catalyzes O_2_ to ·O_2_^−^	Density functional theory calculations reveal that the Co site is more prone to forming ·O_2_^−^ rather than ·OH from H_2_O_2_.	[85]

^a)^ Traditional Fenton reaction involves a chain of reactions initiated by H_2_O_2_ and Fe^2+^. The first step includes the catalytic decomposition of H_2_O_2_ by Fe^2+^ to produce on‐site highly active ∙OH (Equation 1) and the reduction of formed Fe^3+^ by H_2_O_2_ to regenerate Fe^2+^, as well as produce HO_2_∙^[^ ^98^ ^]^ (Equation 2);
(1)
$${\mathrm{Fe}}^{2+}+H_{2}O_{2}\to {\mathrm{Fe}}^{3+}+{\mathrm{OH}}^{-}+\cdot \text{OH},\;k_{1}=\left(40-80M^{-1}s^{-1}\right)$$


(2)
$${\mathrm{Fe}}^{3+}+H_{2}O_{2}\to {\mathrm{Fe}}^{2+}+{\mathrm{HO}}_{2}\cdot +H^{+},\;k_{2}=\left(0.001-0.01M^{-1}s^{-1}\right)$$

^b)^ In addition to Fe, some other metal elements can also generate ∙OH by catalyzing H_2_O_2_, these types of catalytic reactions were defined as Fenton‐like reactions.^[^ ^65d^ ^]^

Abbreviations: ZIF, Zeolitic imidazolate framework; MOF, Metal–organic frameworks; GOx, Glucose oxidase; MRI, Magnetic resonance imaging; PTT, Photothermal therapy; ICB, Immune checkpoint blockade; ECDT, Enhanced chemodynamic therapy.

However, these Fe‐based nanomaterials are still far from satisfactory due to several factors, including the relatively high tumor intracellular pH,^[^ ^66^ ^]^ insufficient endogenous levels of H_2_O_2_, and the conversion of high‐activity Fenton chemical reagents to low‐activity forms, etc.^[^ ^67^ ^]^ To overcome the pH limitation and increase endogenous H_2_O_2_ levels within tumors, glucose oxidase (GOD) was integrated with Fe‐based nanomaterials to supply H_2_O_2_,^[^ ^49^, ^68^ ^]^ such as Fe_5_C_2_,^[^ ^49^, ^69^ ^]^ Fe_3_O_4_,^[^ ^70^ ^]^ γ‐Fe_2_O_3_,^[^ ^71^ ^]^ ferrocene,^[^ ^72^ ^]^ etc. For example, Lin et al. have designed a MnO_2_‐encapsulated and GOD‐loaded magnetic Fe_5_C_2_ core–shell structured nanosystem (Fe_5_C_2_‐GOD@MnO_2_) to provide a source of H_2_O_2_ and O_2_. The acidic TME allows for the decomposition of the MnO_2_ shell into Mn^2+^ and O_2_, and can also trigger the release of GOD. The released GOD can effectively catalyze glucose to generate plenty of H_2_O_2_ in the tumor region under sufficient oxygen conditions, thus accelerating the subsequent Fenton reaction catalyzed by the Fe_5_C_2_ magnetic core in mildly acidic TME.^[^ ^49^ ^]^ Moreover, it has been reported that the overexpressed ferritin heavy chain (FHC) in cancer cells can catalyze rapid oxidation of Fe^2+^ into less reactive Fe^3+^ and sequester Fe in ferritin, which significantly diminishes the antitumor efficacy of the Fenton reaction. To counteract this, small interfering RNA (siRNA)‐embedded amorphous Fe^0^ nanoparticles (Fe^0^‐siRNANPs) have been synthesized to enhance Fenton reaction process through gene‐silencing siRNA‐mediated downregulation of FHC.^[^ ^47a^ ^]^

##### Generation of ^1^O_2_

^1^O_2_ represents the first excited state of the dioxygen molecule, displaying heightened reactivity, and is one of the ROS species generated during the oxidative phosphorylation (OXPHOS) process.^[^ ^73^ ^]^ It readily reacts with biological molecules like unsaturated lipids, and α amino acids of proteins (tryptophan, histidine, methionine), which are integral components of cell and nuclear membranes. This interaction leads to the generation of a range of other reactive intermediates and disrupts the redox homeostasis of tumor cells.^[^ ^74^ ^]^ Furthermore, it has also been clarified that the generation of ^1^O_2_ outside the cell membrane can selectively inactivate membrane‐associated catalase, consequently re‐activating intercellular ROS‐RNS‐driven apoptosis‐inducing signaling.^[^ ^75^ ^]^ Given its potential to achieve selective apoptosis or necrosis through localized generation, coupled with its mild redox capacity (2.2 V/NHE, NHE means normal hydrogen electrode), long half‐life (1 µs vs ≈1 ns (∙OH)), strong versatile inorganic ions resistance, and broad pH tolerance (Table 1), ^1^O_2_ treatment holds promises as a target therapeutic strategy. However, the most convenient and efficient way to generate this species is through the photosensitized excitation of ambient triplet oxygen. Only a few metal‐based nanomaterials consisting of Mo^[^ ^59^, ^76^ ^]^ and Mn^[^ ^77^ ^]^ active centers have the capability to generate ^1^O_2_ without the need for light irradiation. For example, Song et al. have developed a high‐efficiency chemodynamic and chemiluminescenct system comprising ultrathin MnO_x_ nanosheet and semiconducting polymer nanoparticles.^[^ ^77^ ^]^ The ultrathin MnO_x_ nanosheets catalyze the conversion of endogenous H_2_O_2_ to ^1^O_2_ under the trigger of acidity, representing a novel chemodynamic process (Figure 4c). The ^1^O_2_ produced by MnO_x_ can then substitute light and specifically excite thiophene‐based semiconducting polymer nanoparticles to emit photons for near‐infrared chemiluminescent imaging, which greatly amplifies the generation of ^1^O_2_. Besides, Zhao et al. have proposed an energy‐free and tumor‐specific ^1^O_2_ therapy by intravenous delivery of ClO^−^‐loaded ZIF and concurrent intraperitoneal administration of ascorbate.^[^ ^78^ ^]^ Under the TME, ascorbate produced H_2_O_2_ that could diffuse into the tumor cells where it would react with ClO^−^ released from nanocarriers to stoichiometrically produce ^1^O_2_.

##### Generation of ·O_2_^−^

It has been well known that the NADPH oxidases (NOXs) 1–3 can be activated via the assembly of cytosolic subunits and produce ·O_2_^−^.^[^ ^79^ ^]^ Low concentration of the generated ·O_2_^−^ is quickly decomposed by the SODs in the body.^[^ ^80^ ^]^ However, excessive exogenous ·O_2_^−^ readily reacts with proteins, DNA, and lipids, leading to irreversible damage to cellular components and disrupting cell metabolism.^[^ ^81^ ^]^ Therefore, various approaches have been devised to generate ·O_2_^−^, including type I photoreactions based on benzo phenothiazine or Nile blue NIR cationic photosensitizers,^[^ ^80^, ^82^ ^]^ biochemical reactions, and molecular drugs.^[^ ^83^ ^]^ Dai et al. have developed a ROS‐promoting combination drug delivery platform via a biocompatible metal‐polyphenol networks self‐assembly process by encapsulating Dox and platinum prodrugs in nanoparticles.^[^ ^84^ ^]^ The platinum drug and Dox can activate NOXs to generate ·O_2_^−^. Cai et al. constructed the Co single‐atom nanozyme on N‐doped porous carbon via Co−N coordination, which displayed TME‐responsive cascade enzymatic activities with both catalase‐ and oxidase‐like properties.^[^ ^85^ ^]^ The cascade enzymatic reactions enable the decomposition of cellular endogenous H_2_O_2_ to produce ·O_2_^−^ (Figure 4d). By incorporation of Dox as the chemotherapeutic agent, the single‐atom nanozyme‐based platform achieved significantly enhanced antitumor effects.

##### Generation of RNS

**Figure 5:**
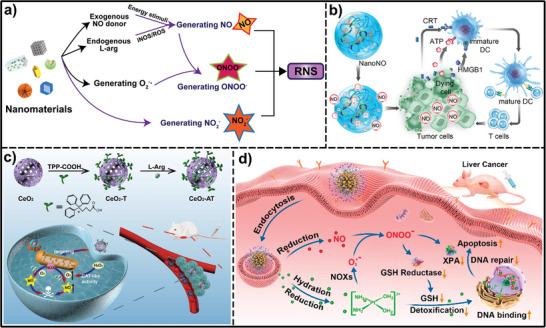
Schematic illustration of nanomaterials induced RNS the generation of RNS under internal stimuli for cancer treatment. a) The approach of stimuli‐responsive nanomaterials to induce RNS generation. b) Nanoassemblies of *S*‐nitrosylation polymers as a NO nanogenerator enable controlled NO release in response to tumoral GSH, inducing immunogenic cell death (ICD) to enhance immunotherapy. Reproduced with permission.^[^ ^146^ ^]^ Copyright 2022, American Chemical Society. c) Schematic illustration of a nanozyme‐based NO generator, CeO_2_‐AT to specifically produce NO under the catalysis of NOS in mitochondria of cancer cells for selective oncotherapy. Reproduced with permission.^[^ ^147^ ^]^ Copyright 2022, Springer Nature. d) A supramolecular Pt/NO prodrug nanoassemblies strategy realized ONOO^−^‐potentiated chemotherapy of liver cancer. Reproduced with permission.^[^ ^148^ ^]^ Copyright 2021, American Chemical Society.

RNS plays an important role in cell signaling, immunity, and tissue homeostasis.^[^ ^141^ ^]^ Typical RNS, including NO, nitroso ion (NO^+^), ONOO^−^, NO_2_, S‐nitrosomercaptan (SNOs), and nitrogen oxides (NO_x_), are derived from the interaction between NO and various ROS^[^ ^142^ ^]^ or from L‐arginine (L‐Arg) by various nitric oxide synthase (NOS) enzymes.^[^ ^143^ ^]^ Similar to ROS, the biological effects of RNS are also concentration‐dependent. For example, ·NO can promote solid tumor angiogenesis at a concentration below 0.1 µm.^[^ ^144^ ^]^ However, at high ·NO concentration(>1 µm), it can form highly oxidizing ONOO^−^, which induces DNA damage, cell apoptosis, and eventual tumor regression.^[^ ^145^ ^]^ Therefore, precise cancer therapy can be realized by enhancing RNS generation (**Figure** **5**a).

As the source of all RNS in the biological systems, NO has a short half‐time and limited diffusion distance within the body (<10 s and <1000 µm, respectively) after its generation.^[^ ^107^ ^]^ Moreover, the biological function of NO is highly dependent on its concentration.^[^ ^149^ ^]^ Therefore, various stimuli‐responsive nanomaterials have been designed to precisely regulate NO generation and achieve satisfactory therapeutic outcomes. Generally, in situ NO generation in the desired tissues can be attained through either endogenous or exogenous triggers, such as ROS, GSH, and hyperthermia. These strategies for NO generation can be classified into two categories: 1) Synthetic NO donors, such as organic nitrates, nitrites, metal–NO complexes, nitrosamines, S‐Nitrosothiols, and diazeniumdiolates,^[^ ^150^ ^]^ are loaded into nanomaterials^[^ ^151^ ^]^ and triggered to release NO by an exogenous or endogenous stimulus such as transition metal ions,^[^ ^152^ ^]^ pH,^[^ ^153^ ^]^ GSH,^[^ ^154^ ^]^ enzymes,^[^ ^155^ ^]^ and nanozymes.^[^ ^156^ ^]^ For example, Wang et al. have developed *S*‐nitrosylation polymers and their nanoassemblies as a NO nanogenerator.^[^ ^146^ ^]^ The NO nanogenerator enables controlled NO release in response to tumoral GSH, inducing immunogenic cell death (ICD) (Figure 5b). In addition, NO donors‐based strategy could be combined with chemotherapeutic drugs to overcome multidrug resistance and enhance the therapeutic effect. For instance, Ji et al. synthesized mitochondria‐targeting BO (α‐CD‐DOX‐NO‐DA NPs) containing both a mitochondria‐penetrating peptide PEG‐(KLAKLAK)_2_CGKRK and α‐cyclodextrin‐based prodrugs for DOX and NO.^[^ ^157^ ^]^ When taken up by cancer cells, such specific mitochondria‐targeted delivery of NO proves crucial in inducing mitochondria dysfunction through facilitating mitochondrial membrane permeabilization and downregulating ATP level, which can inhibit P‐glycoprotein‐related bioactivities and formation of tumor‐derived microvesicles to combat drug resistance and cancer metastasis. 2) Endogenous NO is generated from the nitrogen atom of the terminal guanidine group of arginine in the presence of NOS under physiological conditions or through the oxidation of arginine by some ROS, such as H_2_O_2_.^[^ ^158^ ^]^ Inspired by the biosynthesis of endogenous NO that utilizes L‐Arg as a natural donor,^[^ ^159^ ^]^ a variety of L‐Arg‐based nanosystems have been developed.^[^ ^160^ ^]^ However, the limited oxidation of TME and the availability of L‐Arg pose challenges in generating sufficient NO, limiting the therapeutic effectiveness of L‐Arg‐based nanosystems. To enhance the NO generation in tumor tissues, elevating the oxidation of TME and the supplement of L‐Arg are effective strategies for enhancing antitumor activity. For example, the combination of arginine and GOx is an effective strategy for facilitating NO generation.^[^ ^161^ ^]^ Enzyme mimics that accelerate the production of O_2_ and ^1^O_2_ can also enhance NO generation from arginine.^[^ ^147^, ^162^ ^]^ For instance, Sun et al. have synthesized a nanozyme‐based NO generator, cerium oxide (CeO_2_)‐AT,^[^ ^147^ ^]^ motivated by the fact that mitochondria of cancer cells express excessive NOS. After endocytosis into cancer cells, the generator triggered the production of NO molecules within the mitochondria of cancer cells through the catalytic action NOS, thus disrupting the mitochondrial respiratory chain of tumor cells and further inducing cell apoptosis (Figure 5c). In addition, the generator with CAT‐like activity can catalyze H_2_O_2_ to produce O_2_, which can promote the generation of NO and improve the performance of NO gas therapy. Similarly, Chen et al. have also designed an endogenous generator that concurrently produces ^1^O_2_ and NO by integrating the arginine with Ru nanozyme. Combining the antitumor activity of ^1^O_2_, NO, the chiral Ru nanozymes realize the “cocktail therapy” by inducing tumor cell apoptosis as well as ferroptosis.^[^ ^162b^ ^]^

ONOO^−^, as a product of the reaction between NO and ·O_2_^−^, is far more toxic than most free radicals, including ·OH.^[^ ^163^ ^]^ Such toxicity is attributed to its direct oxidation capacity as well as the formation of carbonate (CO_3_∙^−^) and nitrite (∙NO_2_) radicals through radical‐mediated nitration reactions,^[^ ^164^ ^]^ which induce a variety of detrimental effects, including DNA strand breakage, disruption of membrane structure by lipid peroxidation, direct damage to mitochondria, and the promotion of cell death.^[^ ^165^ ^]^ Due to the transitory lifetime and limited diffusion distance of NO and ∙O_2_^−^, achieving precise control over their simultaneous production within the same cellular locale presents a significant challenge for the efficient generation of ONOO^−^. Deng et al. addressed this challenge by devising a tailored approach involving supramolecular Pt/NO prodrug nanoassemblies for ONOO^−^‐potentiated chemotherapy of liver cancer.^[^ ^148^ ^]^ The supramolecular prodrug nanoassemblies were prepared by supramolecular self‐assembly of the host block copolymer and the two guest prodrugs: NO donor and Pt prodrug. Upon targeted delivery into liver cancer cells through endocytosis, both Pt(II) and NO are intracellularly released to produce a highly toxic ONOO^−^, which in turn downregulates the levels of GSH reductase and xeroderma pigmentosum group A, thus synergistically decreasing detoxification processes and blocking the repair of DNA damage caused by Pt‐based chemotherapy (Figure 5d).

##### Generation of Other Reactive Oxidative Species

Cancer treatment based on oxygen‐free radicals has emerged as a promising clinical approach. However, most solid tumors contain severely hypoxic areas,^[^ ^166^ ^]^ which prevent the formation of oxygen‐free radicals. A feasible solution to address this issue involves the delivery of a precursor compound into the tumor, allowing for the in situ generation of radicals upon activation. Most free radical‐generating precursors are chemically unstable due to their susceptibility to in vivo decomposition, which inevitably increases the side effects. Herein, we provided an overview of the anticancer effects of carbon‐centered radicals (CO, alkyl radicals) and sulfate radicals, and the principles for the design of stimuli‐responsive nanomaterials to precisely control the free radicals release in the tumor site.

**Figure 6:**
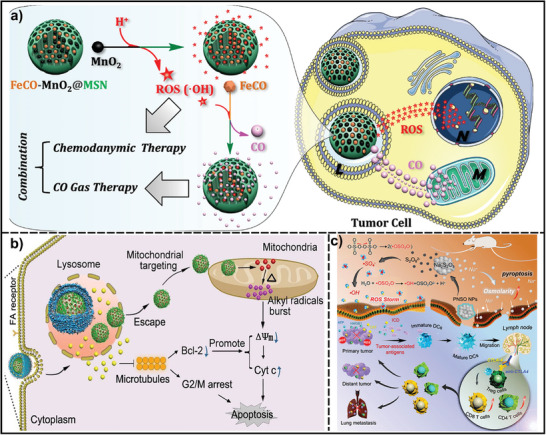
Schematic illustration of nanomaterials induced the generation of other radicals under internal stimuli for cancer treatment. a) The mechanisms for acid‐triggered sequential release of ·OH and CO by the FeCO‐MnO_2_@MSN nanomedicine for synergetic therapy. Reproduced with permission.^[^ ^175c^ ^]^ Copyright 2019, Springer Nature. b) AIPH/MSN‐TPP@Lipo/DTX‐FA NPs released alkyl radicals in mitochondria at high temperatures and then combined with DTX to enhance the cell apoptosis process. Reproduced with permission.^[^ ^176^ ^]^ Copyright 2020, Elsevier. c) PNSO releases sulfate radicals under TME and triggers ROS storms to lead to immunotherapy through the ICD process. Reproduced with permission.^[^ ^177^ ^]^ Copyright 2020, American Chemical Society. Abbreviations: TPP, 5,10,15,20‐Tetraphenylporphyrin.

As an endogenous signaling molecule, carbon monoxide (CO) can inhibit the cytochrome of NOXs and the mitochondrial respiratory chain by interacting with heme, thus increasing the production of ROS and inducing mitochondrial damage and dysfunction in cancer cells.^[^ ^167^ ^]^ Therefore, the use of CO as a therapeutic agent for cancer has already been extensively studied.^[^ ^168^ ^]^ A growing number of CO‐releasing molecules, including metal carbonyl compounds, aldehydes, oxalates, boron carboxylates, and silacarboxylates, have been developed to carry and release CO in response to various endogenous stimuli,^[^ ^169^ ^]^ such as mild acidity,^[^ ^170^ ^]^ overproduced H_2_O_2_,^[^ ^171^ ^]^ and reductive molecules (e.g., GSH)^[^ ^172^ ^]^ or enzymes.^[^ ^173^ ^]^ However, therapies relying on CO‐releasing molecules face many challenges, including limited stability in biological environments, potential toxicity, lack of precise spatial‐temporal control, and low cellular uptake and tissue accumulation. The use of stimuli‐responsive nanomaterials could realize high CO payloads, lower dosage, enhanced biosafety, and targeted CO delivery to specific sites, thus they have been designed to settle aforementioned challenging problems.^[^ ^174^ ^]^ For example, He's group has used mesoporous silica nanoparticles and MOF nanocomplex to encapsulate metal carbonyl compounds to realize the intratumoral acid‐triggered release of CO through a Fenton‐like reaction.^[^ ^175^ ^]^ As shown in **Figure** **6**a, small‐sized MnO_2_ nanoparticles and FeCO were sequentially loaded into mesoporous silica nanoparticles. Within the intratumoral acidic environment, MnO_2_ produced Mn^2+^ and ·OH, subsequently triggering CO release from Fe_3_(CO)_12_. The produced ·OH and CO accelerated DNA damage and rendered mitochondrial collapse, exhibiting unique effects of therapeutic synergy.^[^ ^175c^ ^]^

Alkyl radicals can be generated from some azo‐based initiators, requiring only mild heat stimulus without oxygen participation. These radicals effectively eradicate cancer cells by directly oxidizing cellular components or reacting with oxygen to generate O_2_‐containing free radicals, such as alkoxy and peroxy groups. Leveraging the unique biological feature of the mitochondrial matrix, which maintains a temperature ≈6–9 °C higher than the surrounding cytoplasm, a core–shell structure drug delivery system targeting mitochondria was developed. This system involved loading 2,2′‐azobis[2‐(2‐imidazolin‐2‐yl) propane] dihydrochloride (AIPH) into triphenylphosphine‐modified mesoporous silica nanoparticles, which were then encapsulated by folic acid tagged pH‐sensitive liposomes containing docetaxel.^[^ ^176^ ^]^ Once delivered to mitochondria, AIPH generates alkyl radicals in the high‐temperature environment, leading to cause oxidative damage and enhancing the cellular apoptosis process (Figure 6b).

Sulfate radicals (·SO_4_^−^), as intermediates generated by activation of persulfate or peroxymonosulfate (PMS), have a longer half‐life time (30–40 µs) and higher redox potential (2.5–3.1 V) than most of the free radicals.^[^ ^178^ ^]^ Therefore, combining them with oxygen radicals becomes an advisable synergistic strategy for cancer therapy. In a pioneering study, Liu et al. have prepared phospholipid‐coated Na_2_S_2_O_8_ nanoparticles (PNSO NPs) for the in situ generation of Na^+^ and S_2_O_8_^2−^ through gradual degradation.^[^ ^177^ ^]^ These species can then transform into toxic ·SO_4_^−^ and ·OH, regardless of the levels of H_2_O_2_ and pH within the TME. The substantial production of Na^+^, toxic ·SO_4_^−^ and ·OH results in a surge of osmolarity, rapid cell rupture and lysis, and caspase‐1‐related pyroptosis. Furthermore, these PNSO NPs can regulate the immunosuppressed TME and activate systemic antitumor immune responses to combat tumor metastasis and recurrence (Figure 6c).

In this section, we presented an overview of recent advances in the design and fabrication of responsive nanomaterials to induce excessive reactive oxidative species by taking advantage of TME such as low pH, high GSH concentration, excessive H_2_O_2_, severe hypoxia, etc. A variety of strategies and nanomaterials are extensively exploited for ROS/RNS generation for tumor suppression. These nanosystems exhibit high efficiency, good targeting capacity, stimuli sensitivity, and biocompatibility, thereby making them highly attractive. However, few ROS/RNS‐generating nanosystems have been approved in clinics or clinical trials. The obstacles include unclear pharmacokinetics of these nanomaterials in the human body, difficulty of large‐scale, repeated preparation, etc. The responsive nanomaterials to induce oxygen‐free oxidative species have not yet been widely developed. Considering that most solid tumors contain severely hypoxic areas, this strategy may be a promising research direction. In addition, TME differs significantly from the normal cellular microenvironment and is not limited to the above points. Searching for new TME‐response features will help create more promising nanomaterials and develop more TME‐response strategies.

#### 3.1.2 TME‐Responsive Nanomaterials that Generating Reactive Oxidative Species Under External Stimuli

External energy fields such as light, X‐ray, and US irradiation have the potential to enhance the generation of ROS and improve anti‐tumor effects. In response, numerous external stimuli‐responsive nanomaterials have been rationally designed to achieve a precise and controlled generation of reactive oxidative species. These nanomaterials exhibit intriguing advantages, including minimal or non‐invasiveness and specific spatiotemporal selectivity. According to the external stimuli response sources, they are roughly divided into light, X‐ray, US, and other stimulation methods.

##### Light Stimuli‐Responsive Nanosystems for Inducing the Generation of Reactive Oxidative Species

Due to its merits of spatiotemporal addressability, noninvasiveness, high specificity, and easy operation, phototherapy is considered to be a promising approach for inducing the generation of ROS. Moreover, as an external stimulus, light is also extensively used to control the release behaviors of other free radicals (including NO, CO, carbon‐centered radicals, and other radicals) at precise locations.

##### Light‐Induced ROS Generation

**Figure 7:**
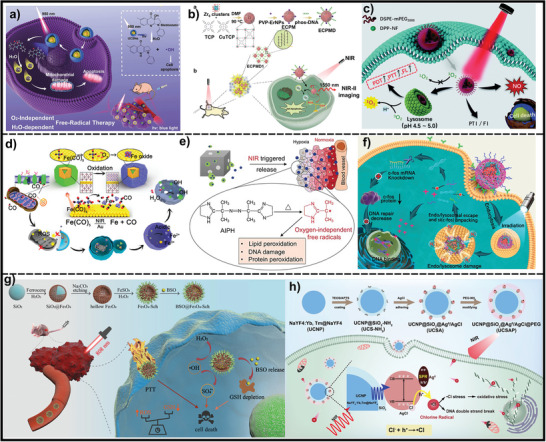
Schematic illustration of nanomaterials generated reactive oxidative species under light‐stimuli for cancer treatment. a) NIR‐I light up‐converted to blue light to irradiate Blebbistatin to generate ·OH through oxygen‐independent PDT. Reproduced with permission.^[^ ^196^ ^]^ Copyright 2021, Wiley‐VCH. b) Schematic illustration of the acidic TME‐responsive ECPM nanohybrids for NIR‐induced PDT and downconverted NIR‐II imaging. Reproduced with permission.^[^ ^200^ ^]^ Copyright 2020, American Chemical Society. c) Schematic illustration of DPP‐NF NPs for controllable “on–off” release of NO under light/dark conditions. Reproduced with permission.^[^ ^211^ ^]^ Copyright 2018, Wiley‐VCH. d) Design of a controlled CO delivery nanomaterial for improving cancer therapy. Reproduced with permission.^[^ ^214^ ^]^ Copyright 2020, American Chemical Society. e) Schematic illustration showing the controlled release and generation of alkyl radicals upon irradiation by NIR laser. Reproduced with permission.^[^ ^217^ ^]^ Copyright 2017, Wiley‐VCH. f) CNPPtCP/si(c‐fos) generates oxygen‐independent N_3_∙ for endo/lysosomal escape, accompanied with the subsequently released Pt(II) and si(c‐fos) for synergistic cancer therapy. Reproduced with permission.^[^ ^228^ ^]^ Copyright 2020, American Chemical Society. g) BSO@Fe_3_O_4_‐Sch nanocomposites produce ∙SO_4_^−^ under the attacking of abundant ·OH, which is produced through Fenton reaction trigged by NIR laser. Reproduced with permission.^[^ ^229^ ^]^ Copyright 2021, Wiley‐VCH. h) Schematic diagram of UCSAP synthesis and ·Cl generation for hypoxic tumor therapy. Reproduced with permission.^[^ ^231^ ^]^ Copyright 2020, Wiley‐VCH.

In the field of nanomaterials that generate ROS by light, photodynamic strategy (photodynamic therapy: PDT) has extensively been used to treat various cancers.^[^ ^179^ ^]^ Specifically, PDT works via two mechanisms: type I and type II reactions.^[^ ^180^ ^]^ In type‐I reactions, the charge transfer among photoexcited photosensitizers with triple oxygen (^3^O_2_) or water molecules leads to the generation of ·OH and ·O_2_^−^.^[^ ^181^ ^]^ While in type II reactions, the excited triplet of photosensitizers directly transfers its energy to ^3^O_2_, yielding reactive ^1^O_2_ for therapeutic purposes.^[^ ^182^ ^]^ To date, ≈20 photosensitizers have been commercialized or used in clinical trials. However, these available photosensitizers face several major challenges, such as limited tissue penetration depth, low targeting efficiency, restricted O_2_ supply due to hypoxic microenvironments, and elevated levels of GSH. To overcome these grave challenges, transition metal coordination complexes, organic‐fluorophores, and semiconducting polymer nanoparticles have been extensively exploited as attractive PDT agents.^[^ ^183^ ^]^ Inorganic nanomaterials, including TiO_2_,^[^ ^184^ ^]^ ZnO,^[^ ^185^ ^]^ g‐C_3_N_4_,^[^ ^186^ ^]^ Pt‐CDs,^[^ ^187^ ^]^ Bi and its oxides,^[^ ^188^ ^]^ etc., have emerged as promising ideal photosensitizer candidates for PDT. Various nanosystems‐based solutions have been developed to improve the PDT effect. To enhance the effects of PDT in deep tissues, photosensitizers with a high two‐photon absorption have been designed, including Au nanoclusters,^[^ ^189^ ^]^ quantum dots,^[^ ^190^ ^]^ carbon nanomaterials,^[^ ^191^ ^]^ silica nanoparticles,^[^ ^192^ ^]^ polymer nanoparticles,^[^ ^193^ ^]^ or other nanoparticles.^[^ ^194^ ^]^ Moreover, applying light conversion nanomaterials which can convert NIR light and X‐rays with high tissue penetration depth into visible light has emerged as a new technology for activating photosensitizers for deep‐tissue PDT.^[^ ^195^ ^]^ For instance, Chen et al. have used multiphoton excited up‐conversion nanoparticles (UCNPs) as energy transducers to convert NIR light into blue light through the fluorescence resonance energy transfer (FRET) strategy,^[^ ^196^ ^]^ thus realizing oxygen‐independent ·OH bursts via H_2_O‐mediated photocleavage of blebbistatin^[^ ^197^ ^]^ (**Figure** **7**a). Chen et al. have engineered a nanosystem that consists of SrAl_2_O_4_:Eu^2+^ core with double‐layer silica coats. Photosensitizer merocyanine 540 is embedded into the mesoporous silica layer.^[^ ^198^ ^]^ Upon irradiation, SrAl_2_O_4_:Eu^2+^ core converts X‐rays to visible light photons, thus activating merocyanine 540 to trigger the production of ^1^O_2_ for PDT therapy. The substantial overlap between the X‐ray‐excited optical luminescence of SrAl_2_O_4_:Eu^2+^ and the excitation wavelength of merocyanine 540 augments the effectiveness of photosensitizer activation with low‐energy X‐rays. Moreover, persistent luminescence nanoparticles that maintain long afterglow luminescence after the removal of the light source are an emerging class of luminescent materials as transducers for X‐ray‐induced PDT.^[^ ^199^ ^]^ Yang's group has presented a strategy for depth‐independent and repeatable PDT by using low‐dose X‐ray‐activatable W(VI)‐doped ZnGa_2_O_4_: Cr NPs as an excitation source and Zn(II) phthalocyanine tetrasulfonic acid as photosensitizer. The persistent luminescence continues exciting the coupled photosensitizer after the X‐ray irradiation has been removed, resulting in a significantly reduced X‐ray dosage (≈0.18 Gy) to minimize the side effects of PDT treatment.^[^ ^199b^ ^]^ The combination of the deep penetration of X‐rays and the specific targeting of PDT minimizes the side effects caused by X‐ray irradiation with ultra‐high doses and overcomes the limitations of shallow light irradiation penetration. Furthermore, Photosensitizers have also been conjugated with photothermal nanomaterials to achieve PTT‐enhanced PDT owing to the heat produced by PTT can increase blood flow and thus improve oxygen supply to enhance PDT efficiency.^[^ ^200^ ^]^

To enhance the specific distribution of photosensitizers in tumor sites, several activatable nanomaterials responsive to the acidic TME were recently engineered for tumor‐specific delivery of the photosensitizer and enhanced PDT against tumors.^[^ ^201^ ^]^ Our group has also reported a pH‐responsive i‐motif DNA‐mediated strategy to achieve selective tumor accumulation of photosensitizer and enable deep‐tissue PDT.^[^ ^202^ ^]^ As shown in Figure 7b, the virus‐like erbium‐based rare‐earth nanoparticles decorated copper doped MOFs nanohybrids were modified with pH‐responsive i‐motif DNA strands via the metal–phosphate coordination interactions. Within the acidic TME, the i‐motif DNA strands can form quadruplex structures, resulting in the assembly of nanohybrids and selective tumor accumulation. Under low‐energy photon excitation, the emitted visible photon from the ErNPs could be easily harvested by the porphyrin ligands in copper‐doped MOFs for NIR‐induced photodynamic therapy.

##### Light‐Controlled Release of NO

Various NO donors with photostability, such as S‐nitrosothiols, metal‐NO complexes, *N*‐nitrosamines, nitrobenzene, etc., have been developed for NO release. However, most light‐responsive NO donors are activated by ultraviolet and visible light, thus limiting their tumor application. To achieve deep‐tissue penetration, an increasing number of nanocarriers, including carbon nanostructures,^[^ ^203^ ^]^ silica NPs,^[^ ^204^ ^]^ TiO_2_ NPs,^[^ ^205^ ^]^ noble metal NPs,^[^ ^206^ ^]^ metal sulfide NPs,^[^ ^207^ ^]^ Fe_3_O_4_ NPs,^[^ ^208^ ^]^ copolymers,^[^ ^209^ ^]^ and UCNPs^[^ ^210^ ^]^ have been exploited to encapsulation of photoactive NO donors to enable photocontrolled release of NO. Zhang et al. first used the NIR to visible upconversion properties of lanthanide cations doped NaYF_4_ nanocrystals to trigger photochemical NO release from an iron nitrosyl complex.^[^ ^210b^ ^]^ Guo et al. have reported ruthenium nitrosyl and TPP‐functionalized N‐GQD as a mitochondria‐targeting nanoplatform. The nanoplatform specifically localized in the mitochondria and released NO accompanied by PTT when irradiated with 808 nm light.^[^ ^203a^ ^]^ l‐Arg/Dox‐loaded liposomal Au@CuS yolk–shell nanoparticles were designed to sequentially release NO and Dox for multidrug resistance (MDR) cancer therapy under 808 nm laser irradiation.^[^ ^206^ ^]^ The sequential release of NO and Dox could significantly inhibit P‐gp expression and enhance Dox accumulation in Dox‐resistant MCF‐7/ADR cells. Wang et al. have used the covalent bonding of a NIR‐responsive NO photodonor (4‐nitro‐3‐trifluoromethylaniline, NF), and pH‐sensitive unit (dimethylaminophenyl‐) and diketopyrrolopyrrole core to achieve ^1^O_2_ generation and NO release under weakly acidic conditions of lysosomes (pH 4.5–5.0) (Figure 7c).^[^ ^211^ ^]^

##### Light‐Controlled Release of CO

The photolytic features of metal carbonyl complexes have been integrated with nanocarriers for the photocontrolled release of CO.^[^ ^212^ ^]^ For instance, graphene oxide nanosheet was employed as carriers for Mn‐carbonyl and ruthenium carbonyl to achieve NIR‐responsive release of CO.^[^ ^213^ ^]^ Zhang et al. have designed a controlled CO delivery nanomaterial by encapsulating iron pentacarbonyl (Fe(CO)_5_) within an Au nanocage under an oxygen‐free atmosphere^[^ ^214^ ^]^ (Figure 7d). Under aerobic conditions, Fe(CO)_5_ is transformed into iron oxide on the surface of the Au nanocage in a controllable manner, efficiently preventing leakage and oxidation of the encapsulated Fe(CO)_5_, thereby enhancing its stability and biocompatibility. Under NIR irradiation, the photothermal effect of Au nanocages triggers the decomposition of the encapsulated Fe(CO)_5_ to generate CO and iron resulting in remarkable synergistic effects in cancer cells, as presented by inducing mitochondrial damage, autophagy, and the destruction of autolysosome. In addition, inspired by CO_2_ natural capture and transformation, Zhang's group has prepared an innovative CO‐producing photocatalyzed nanomaterial, Ag_3_PO_4_ doped carbon‐dot‐decorated C_3_N_4_ nanoparticles functionalized with histidine‐rich peptide, which could transform tumor endogenic CO_2_ to CO under the precise control of body‐penetrating 630 nm light, significantly enhancing chemotherapy toward prostate cancer cells.^[^ ^215^ ^]^

##### Light‐Responsive Generation of Carbon‐Centered Radicals

The thermal decomposition of azo initiators to produce alkyl radicals is constrained at physiological temperatures. The NIR‐based photothermal effect provides a feasible solution to accelerate the generation of free radicals. In 2017, Zhang et al. presented a novel therapeutic strategy for NIR‐induced generation of alkyl radicals.^[^ ^216^ ^]^ An initiator of 2,2‐azobis[2‐(2‐imidazolin‐2‐yl)propane] dihydrochloride (AIBI) was loaded in the hollow interiors of gold nanoclusters (AuNCs), followed by coating of thermal‐responsive poly(*N*‐isopropylacrylamide‐*co*‐acrylamide) as a gatekeeper to form AIBI@AuNC‐copolymer. Under NIR irradiation, the plasmonic heating of AuNCs facilitated the decomposition of AIBI to generate alkyl radicals, as well as causing the phase transition of the copolymer to release the blocked alkyl radical. Similarly, Xia et al. have also demonstrated the use of AuNCs filled with AIPH (the same as AIBI) along with phase‐change material to achieve a controlled generation of free radicals for cancer therapy^[^ ^217^ ^]^ (Figure 7e). Since the photothermal efficiency of Au nanocages is not satisfactory, nanomaterials with higher photothermal efficiency were conjugated to radical initiators to generate alkyl radicals, including porphyrin‐based organic nanoparticles,^[^ ^218^ ^]^ carbon‐coated iron carbide (Fe_5_C_2_) nanoparticles,^[^ ^219^ ^]^ CuFeSe_2_@BSA,^[^ ^220^ ^]^ MoS_2_@PCM,^[^ ^221^ ^]^, Bi_2_Se_3_,^[^ ^222^ ^]^, and others.^[^ ^176^, ^223^ ^]^ Another carbon‐centered radical, trifluoromethylation radicals (·CF_3_), possesses a stronger electrophilic ability than ROS when it comes to attacking unsaturated bonds and most amino acids in organisms.^[^ ^136^ ^]^ Zheng et al. have presented a triaryl methane/CF_3_SO_2_Na‐based liposomal nanosystem that efficiently promotes ferroptosis and apoptosis to treat drug‐resistant tumors.^[^ ^224^ ^]^ Under 630 nm light irradiation, the nanosystem generated ∙O_2_^−^, ·CF_3_, and SO_2_ within drug‐resistant tumor cells. The generated substance is coordinated to disrupt the redox balance of the drug‐resistant system by increasing oxidation and decreasing reduction, prompting the accumulation of a large amount of lipid peroxidation to induce ferroptosis.

##### Light‐Responsive Generation of Other Radicals

Photoactive platinum azido complexes exhibit remarkable stability in the absence of light. However, upon irradiation, they can be activated to generate azidyl radicals (N_3_∙), ·OH, and anticancer‐active Pt(II) species. This property underscores their potential as photosensitizers for O_2_‐independent PDT.^[^ ^225^ ^]^ Based on the unique characteristics of platinum azido complexes, Huang et al. have bound a Pt(IV)–azide complex and demethylcantharidin to form a multifunctional single‐drug, which was further conjugated to an amphiphilic block copolymer to form a micelle.^[^ ^226^ ^]^ Under ultraviolet A irradiation and endosomes/lysosomes environments, the micelle was activated to release Pt, N_3_∙, and demethylcantharidin, showing great potential for overcoming acquired tumor resistance to cisplatin. Furthermore, their group further encapsulated Pt(IV)‐azide complexes in various nano/microcarriers for tumor therapy.^[^ ^227^ ^]^ For example, they connected the photoreactive Pt(IV) azido precursor drug to siRNA for synergistic cancer therapy^[^ ^228^ ^]^ (Figure 7f).

Light activation of peroxodisulfate and peroxymonosulfate‐based nanosystems could generate ∙SO_4_^−^, irrespective of the amount of H_2_O_2_, O_2_, and pH value. As an avant‐garde paradigm, Wu et al. constructed a multifunctional hybrid nanoplatform with a hollow Fe_3_O_4_ core and cell pseudopod‐like Schwertmannite shell. The Fe_3_O_4_ core conducts Fenton reaction catalysts to produce ·OH, while Schwertmannite shells convert SO_4_^2−^ into ∙SO_4_^−^ when exposed to abundant ·OH (Figure 7g).^[^ ^229^ ^]^ The combination of dual‐radical production, Fe_3_O_4_ nanocrystal‐mediated PTT, and GSH depletion causes an imbalance in the intracellular redox state, eventually leading to cell death. Besides, Ding et al. have prepared peroxymonosulfate‐loaded hollow mesoporous CuS NPs with polyethyleneimine modification for photothermal‐enhanced sulfate radical nanotherapy in melanoma. Upon irradiation with 1064 nm light, the copper ions are initially released from CuS, followed by the effective activation of PMS to generate toxic ∙SO_4_^−^ and ∙OH, which is independent of the O_2_ and H_2_O_2_ content within the tumor.^[^ ^230^ ^]^

Chlorine radical (·Cl), with an oxidation potential (2.47 V) second only to ·OH, has a longer lifespan and diffusion distance, and also shows stronger and faster reactivity to certain electron‐rich substances by nucleophilic addition (Table 1). Since there are many nucleophilic atoms and functional groups in biological molecules, ·Cl holds enormous potential for highly efficient tumor eradication via ·Cl‐induced cellular stress.^[^ ^232^ ^]^ Bu's group^[^ ^231^ ^]^ has designed the ·Cl nano‐generators with a SiO_2_‐coated UCNPs interior and decorated with Ag^0^/AgCl hetero‐dots on the outside. As illustrated in Figure 7h, upon NIR irradiation, the short‐wavelength emission light UCNPs catalyze Ag^0^/AgCl to generate ·Cl independent of O_2_/H_2_O_2_, which induces cellular ·Cl stress and DNA double‐strand breaks in tumor cells.

In summary, as one of the most common excitation forms of external energy fields, light stimuli‐responsive nanomaterials capable of generating reactive oxidative species have matured significantly. The clinicalization of photosensitizers has propelled extensive research into light‐induced ROS nanomaterials. Impressive strides have been made in the utilization of light to activate novel free radicals such as CO, ∙SO_4_^−^, and ·Cl. Future efforts in this area should focus on enhancing the penetration ability of light (extending into the NIR‐II region and beyond) while maintaining efficient generation of oxidative species, aiming to achieve applications that are closer to clinical.

##### X‐Ray Stimuli‐Responsive Nanosystems for Inducing Reactive Oxidative Species Generation

**Figure 8:**
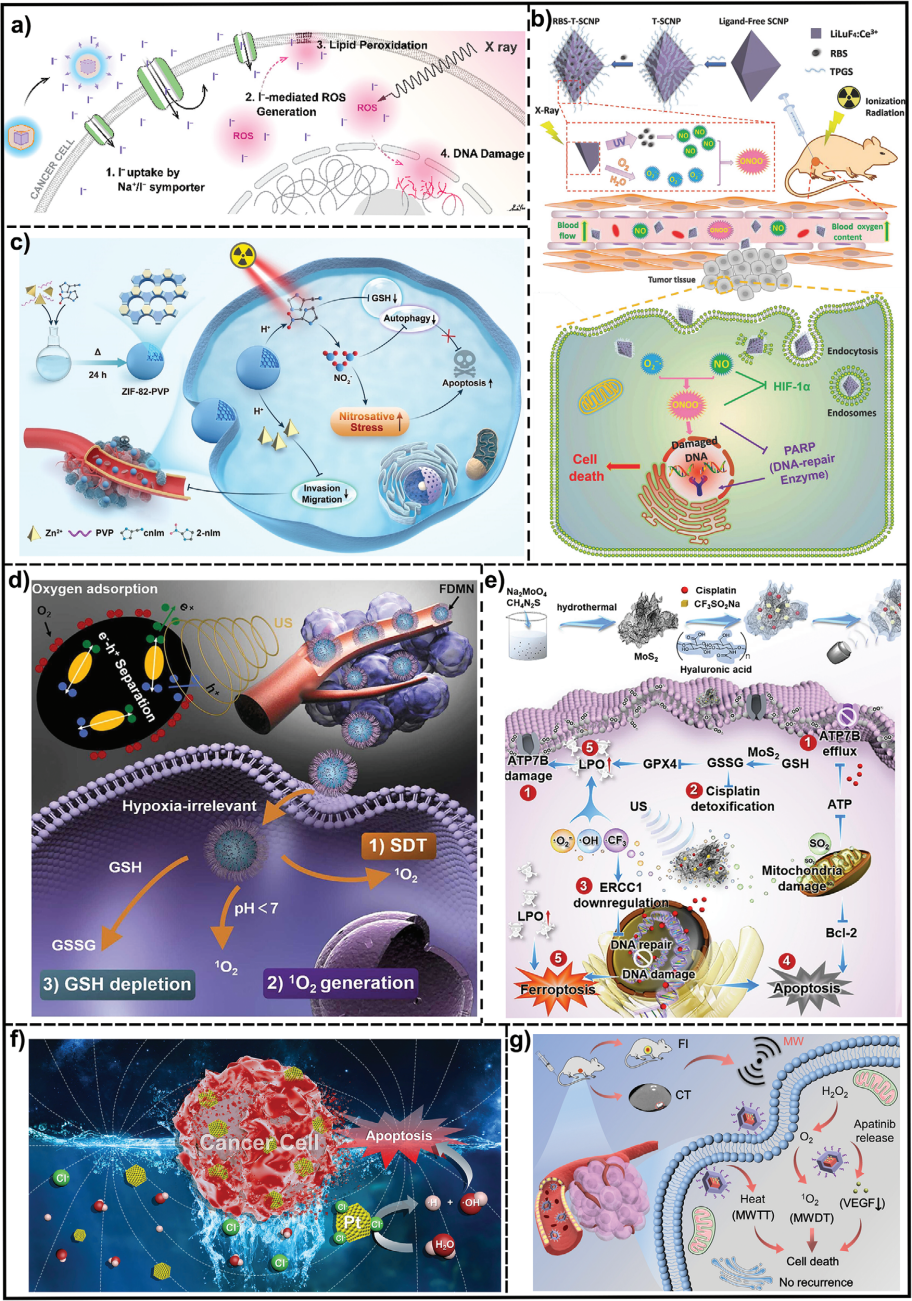
Schematic illustration of nanomaterials generated reactive oxidative species under external stimuli for cancer treatment. a) Schematic illustration of the radiation‐induced production of ROS by PMAO‐KI NPs. Reproduced with permission.^[^ ^246^ ^]^ Copyright 2021, American Chemical Society. b) Schematic illustration showing the synthetic route to RBS‐T‐SCNPs and the X‐ray‐controlled ONOO^−^ generation for on‐demand cancer radiotherapy. Reproduced with permission.^[^ ^248^ ^]^ Copyright 2018, Wiley‐VCH. c) Schematic illustration of the electrophilic ligands released by ZIF‐82‐PVP generate NO_2_^−^ under X‐ray irradiation to enhance lethal nitrosative stress. Reproduced with permission.^[^ ^249^ ^]^ Copyright 2021, Wiley‐VCH. d) Fe‐doped multivalent manganese oxide nanoparticles (FDMNs) via vacancy engineering strategy as a hypoxia‐irrelevant sonosensitizer for enhanced sonodynamic therapy. Reproduced with permission.^[^ ^268^ ^]^ Copyright 2022, Elsevier. e) Schematic of ·OH, ·CF_3_, and SO_2_ formation process of HA@MoCF_3_Pt NPs and mechanism of inhibiting cisplatin resistance under US irradiation. Reproduced with permission.^[^ ^137^ ^]^ Copyright 2022, Elsevier. f) Schematic illustration of ·OH generation by PtNPs under the electric field. Reproduced with permission.^[^ ^275^ ^]^ Copyright 2019, Wiley‐VCH. g) Schematic diagram of BMCAP generates ROS and heat for tumor therapy under MW irradiation. Reproduced with permission.^[^ ^286^ ^]^ Copyright 2022, Elsevier.

X‐rays have a higher penetration in the human body than NIR light.^[^ ^233^ ^]^ More importantly, X‐ray irradiation exposure leads to the radiolytic formation of various ROS, including ∙O_2_^−^, making X‐Ray an ideal excitation source for ROS generation.^[^ ^234^ ^]^ A great mass of multifunctional nanomaterials has been developed for augmenting X‐rays‐induce ROS generation inside tumor tissues and elevating the antitumor therapeutic impact. They can be commonly divided into three types: 1) Light conversion nanomaterials for X‐ray‐induced PDT. These nanomaterials can convert X‐rays into visible lights to activating photosensitizers, including scintillating NPs,^[^ ^18^, ^235^ ^]^ persistent luminance NPs,^[^ ^199^ ^]^ aggregation‐induced emission (AIE) NPs,^[^ ^236^ ^]^ and nanoscale MOFs.^[^ ^237^ ^]^ Recently, Wang et al. have prepared a class of organic phosphorescence nanoscintillators based on 9,9’‐(6‐iodophenoxy‐1,3,5‐triazine‐2,4‐diyl)bis(9H‐carbazole), which act in a dual capacity as scintillators and photosensitizers. This approach demonstrated great potential for deep tumor treatment with a low dose of 0.4 Gy and negligible adverse effects.^[^ ^238^ ^]^ 2) High atomic number (high‐Z) elements‐based nanomaterials. They respond to ionizing radiation and enhance the therapeutic effect of radiotherapy, including noble metals (Au, Ag, Pt, etc.), rare earth elements (Gd, Ho, Tm, etc.), and other heavy elements (Hf, Ta, W, Bi, etc.).^[^ ^239^ ^]^ Moreover, high‐Z elements can be integrated with semiconductor nanomaterials to further enhance the therapeutic effect of radiotherapy.^[^ ^240^ ^]^ For instance, Wang et al. have used Bi and I as High‐Z elements to fabricate ultrasmall semiconductor materials to photocatalytic generate ROS for radiosensitization.^[^ ^241^ ^]^ However, previous studies have shown that DNA repair occurs within the first 5 min after exposure to X‐ray irradiation, and then cells activate the DNA damage response (DDR) pathway to repair damaged DNA.^[^ ^242^ ^]^ PTT can inhibit DNA repair, improve the permeability of NPs in tumor cells, and boost the O_2_ perfusion to sustain O_2_‐dependent therapy. Therefore, many multifunctional nanoplatforms have been designed to achieve PTT‐enhanced radiotherapy.^[^ ^243^ ^]^ For example, our group has utilized W_18_O_49_ nanospheres to generate intracellular ^1^O_2_ and ·OH under X‐ray irradiation, resulting in three‐times enhancement in the inhibition of 4T1 cell proliferation.^[^ ^244^ ^]^ Moreover, to increase O_2_ concentrations in hypoxia tumor tissues, oxygen‐carrying and oxygen‐generating strategies have been also employed to improve nanomaterials‐sensitized radiotherapy. Gu's group synthesized SnS_2_@Fe_3_O_4_ nanocomposite consisting of SnS_2_ nanoplates and Fe_3_O_4_ quantum dots and proposed a concept of X‐ray‐facilitated redox cycling of peroxidase‐like nanozyme for high‐efficiency and persistent ROS generation in tumor therapy.^[^ ^245^ ^]^ 3) Organic small molecule‐linked nanomaterials. For example, Xie et al. have reported a potassium iodide NP‐based radiosensitizer. By employing the Na^+^/I^−^ symporter for iodine delivery and radiosensitization, they achieved complete tumor eradication in 80% of treated animals without inducing additional toxicity^[^ ^246^ ^]^ (**Figure** **8**a).

X‐rays are also used as external stimuli to penetrate the biological tissues for RNS generation.^[^ ^247^ ^]^ The simultaneous production of NO and ·O_2_^−^ in the same region presents a significant challenge for the efficient generation of ONOO^−^. To solve this issue, Gu et al. have introduced theranostic scintillating nanoparticles containing Ce‐doped LiLuF_4_ and UV‐responsive NO donors Roussin's black salt.^[^ ^248^ ^]^ The Ce‐doped LiLuF_4_ can not only act as radiosensitizers for enhancing the yield of ROS, but also convert X‐ray into UV light to activate the photoactive Roussin's black salt to release NO (Figure 8b). Such simultaneous release of NO and ·O_2_^−^ ensures the efficient, X‐ray‐controlled generation of ONOO^−^ in tumors, consequently improving the radiotherapy efficiency by directly damaging DNA, inhibiting the PARP‐associated DNA‐repair process and overcoming the hypoxia‐associated resistance in radiotherapy. Bu's group^[^ ^249^ ^]^ has proposed a strategy for the treatment of hypoxic prostate cancer based on X‐ray‐induced nitrosative stress, using an electrophilic ZIF‐82. After internalization, pH‐responsive ZIF‐82‐PVP nanoparticles release electrophilic ligands and Zn^2+^. The released electrophilic ligands were able to capture low‐energy electrons derived from X‐rays to produce NO_2_^−^, resulting in the inhibition of autophagy and increasing lethal nitrosative stress levels (Figure 8c).

##### US Stimuli‐Responsive Nanosystems for Inducing ROS Generation

Compared with UV–vis light and NIR light, US offers superior tissue penetration capabilities (up to 20 cm depth by 1 MHz US wave) and can enhance vasculature permeability, which improves the cellular uptake and tissue permeability of therapeutic agents for the treatment of tumors. More importantly, low‐intensity US irradiation can activate sonosensitizers concentrated in both tumors and tumor neovascular epithelial cells to produce a large amount of cytotoxic ROS such as ^1^O_2_ and ·OH through the cavitation effect and sonoluminescence (called sonodynamic effect, SDT for short).^[^ ^250^ ^]^ The ROS produced by SDT have the potential to destroy organelles of tumor cells and damage tumor vascular epithelial cells to release thromboxane, resulting in forming thrombus in tumor blood vessels and causing ischemic necrosis of tumor tissues. Thus, SDT has been widely explored as an external stimulus for inducing ROS generation.

Sonosensitizers are typically categorized into two main groups: inorganic sonosensitizers and organic sonosensitizers. Organic sonosensitizers include porphyrins and their derivatives,^[^ ^251^ ^]^ photosensitizing substances,^[^ ^252^ ^]^ etc.^[^ ^253^ ^]^ However, organic sonosensitizers suffer from hydrophobicity, low bioavailability, and low accumulation in tumor sites, greatly restricting the efficacy of SDT. To address these challenges, researchers have extensively explored the use of both inorganic and organic nanocarriers. Like other O_2_‐dependent therapies, insufficient oxygen supply in the hypoxic microenvironment also impacts the efficacy of SDT. Therefore, oxygen‐carrying and oxygen‐generating strategies have been widely used to moderate hypoxia and enhance the efficacy of SDT. For example, Chen and co‐workers have designed an oxygen‐self‐produced SDT nanoplatform by using fluorocarbon‐chain‐functionalized hollow mesoporous organosilica NPs as carriers to load IR780. This nanoplatform effectively overcame the hypoxia‐specific transportation barriers, supplied sufficient oxygen to hypoxic PANC‐1 cells, especially upon exposure to US irradiation, and alleviated hypoxia.^[^ ^254^ ^]^ Moreover, to reduce the toxicity of sonosensitizers to healthy tissues, TME‐responsive smart nanomaterials have been developed for completing the effective accumulation at tumor sites. For instance, Wang et al. have reported new sonosensitizers consisting of porphyrins, chelated Cu^2+^, and PEG. The overexpressed GSH in the tumor reduces Cu^2+^ to generate Cu(I), leading to precise SDT with minimized side effects.^[^ ^255^ ^]^

Inorganic sonosensitizers can overcome the inherent drawbacks of organic sonosensitizers, with high hydrophilicity, better US stability, and lower phototoxicity. Among inorganic sonosensitizers, TiO_2_ was the first inorganic sonosensitizer in SDT due to its ability to generate ROS via the dispersion of electrons and holes.^[^ ^256^ ^]^ However, the wide bandgap and rapid electron‐hole recombination (50 ± 30 ns) of pure TiO_2_ result in a low yield of ^1^O_2_. To tackle this problem, three types of strategies have been developed to suppress the recombination of electrons and holes: integrating TiO_2_ with other multifunctional NPs to form nanocomposite,^[^ ^257^ ^]^ enhancing its electroconductivity,^[^ ^258^ ^]^ or creating an oxygen‐deficient layer.^[^ ^259^ ^]^ For example, Geng and colleagues have designed semiconductor *p‐n* junctions using pyridine N‐doped carbon dots (N‐CDs) as a *p*‐type semiconductor and oxygen‐deficient TiO_2−x_ nanosheets as a *n*‐type semiconductor. The rate constants of ^1^O_2_ and ·OH generation by US‐excited N‐CDs@TiO_2−x_ *p‐n* junctions are 4.3 and 4.5 times higher than those of TiO_2_, respectively, exhibiting enhanced sonodynamic performance and GSH depletion capability for complete eradication of malignant tumors without relapse.^[^ ^260^ ^]^ Besides TiO_2_‐based sonosensitizers, other inorganic semiconductor nanomaterials have also been explored as SDT sonosensitizers to promote ROS production, which include WO_x_,^[^ ^261^ ^]^ VS_4_,^[^ ^262^ ^]^ black phosphorus (BP),^[^ ^263^ ^]^ heterostructures,^[^ ^264^ ^]^ TiH_1.924_ nanodots,^[^ ^265^ ^]^ and defects/vacancies‐containing nanomaterials,^[^ ^266^ ^]^ layered double hydroxides (LDHs),^[^ ^267^ ^]^ etc. For instance, Wang et al. have demonstrated that ZIF‐8 nanocrystals could be used as both a bioactive anticancer agent and a sonosensitizer for efficient cancer therapy.^[^ ^266b^ ^]^ The specific coordinatively unsaturated Zn–N active site on the surface of ZIF‐8 greatly facilitates ROS generation through enhancing the electron transfer to the lower unoccupied molecular orbital (LUMO) via ligand to metal charge transfer (LMCT) under US irradiation, while the release of Zn^2+^ interrupts zinc homeostasis. Moreover, SDT still suffers from impaired efficacy in the treatment of most tumors due to insufficient oxygenation. Considerable efforts have been devoted to self‐generation of O_2_ at tumor sites. Sun et al. have synthesized Fe‐doped multivalent manganese oxide nanoparticles via a vacancy engineering strategy.^[^ ^268^ ^]^ These nanoparticles not only limited the recombination of electron–hole pairs, but also absorbed enough oxygen on the surface, thus bestowing nano‐sonosensitizers with a hypoxia‐irrelevant ^1^O_2_ generation ability (Figure 8d).

In addition to various strategies for increasing ROS generation in SDT, US irradiation has been used to increase the generation of other radicals,^[^ ^269^ ^]^ including RNS, carbon‐centered radicals, and sulfate radicals. In 2010, Gu's group^[^ ^270^ ^]^ designed NO microreactors for US‐triggered NO generation and MRI localization of the reactor by using a double emulsion method to simultaneously encapsulate L‐Arg and superparamagnetic nanoparticles. Jin et al. have also developed a US‐triggered NO release nanoplatform by encapsulating a NO‐releasing molecule into a rattle‐type nano‐carrier of superparamagnetic iron oxide/hollow mesoporous silica nanoparticles, and the amount of released NO can be finely tuned by adjusting the power of the US.^[^ ^271^ ^]^ Guo et al. have reported a US‐responsive drug delivery system by encapsulating an AIPH‐loaded C‐TiO_2_ hollow nanoshells functionalized with a platelet membrane, which can efficiently produce ROS and alkyl radicals upon US excitation.^[^ ^272^ ^]^ Du et al. have developed a smart nanoplatform for multimodal synergistic therapy using MoS_2_ nanoflowers as both a sonosensitizer and nanozyme, CF_3_SO_2_Na as a CF_3_· precursor and cisplatin as the chemotherapeutic drug.^[^ ^137^ ^]^ Under US irradiation, MoS_2_ nanoflowers catalyze the decomposition of H_2_O_2_ and H_2_O to produce ·OH· and ·O_2_^−^, respectively. Then, the produced OH· subsequently triggers the decomposition of CF_3_SO_2_Na into CF_3_·. The generation of ·OH·, ·O_2_^−^ and CF_3_· could reverse the cisplatin resistance and intensify ovarian cancer treatment by activating apoptosis and ferroptosis programs (Figure 8e). Moreover, the integration of the ∙SO_4_^−^ generation agent, PMS, into a Pd‐catalyzed hydrogenated mesoporous titanium dioxide exhibited excellent US‐excited ROS generation under the support of Pd nanozyme‐mediated O_2_ supply and concurrently ·SO_4_^−^ production, which remarkably enhanced the therapeutic efficacy of SDT for cancer.^[^ ^273^ ^]^

##### Other Stimuli‐Responsive Nanosystems for Inducing Reactive Oxidative Species Generation

Besides the three common external stimuli‐responsive nanosystems mentioned above, various nanomaterials based on electrical field, magnetic field, and microwave have also been developed for inducing ROS generation.

Electrodynamic therapy (EDT) refers to the use of an external electric field to generate ROS to destroy tumor cells.^[^ ^274^ ^]^ Nanomaterials can amplify the ROS generation ability of EDT. In 2019, Liu's group discovered that the surface of Pt NPs reacts with H_2_O and Cl^−^ to generate ·OH under the action of an electric field^[^ ^275^ ^]^ (Figure 8f). In vivo experiments showed that this strategy could eliminate surface tumors larger than 500 mm^3^. After that, the combination of Pt‐based EDT with starvation therapy and chemotherapy has been tried to improve ROS‐based dynamic therapy.^[^ ^276^ ^]^ Moreover, the high level of Cl^−^ will promote the production of ·OH in EDT. Introducing exogenous Cl^−^ to tumor tissues or transporting extracellular Cl^−^ into cancer cells using a chloride ion transporter has been shown to significantly improve ROS generation in EDT.^[^ ^277^ ^]^

As a noninvasive, controllable, and penetrating external stimulus, magneto‐thermal therapy (MHT) has been widely applied in clinical tumor ablation.^[^ ^278^ ^]^ However, it is difficult to achieve complete tumor ablation in the site by heat alone due to the uneven enrichment of magnetic nanomaterials in tumors. To address this issue, Shi's group has proposed a novel magnetoelectrodynamic therapy method (MEDT) by employing CoFe_2_O_4_–BiFeO_3_ (CFO‐BFO) magnetoelectric nanoparticles for ·OH and ·O_2_^−^ generation.^[^ ^279^ ^]^ Under the stimulation of alternating magnetic fields, the magnetostrictive CFO core and the multiferroic BFO shell produce magneto‐generated electrons and holes, ultimately facilitating the generation of ROS for effective tumor therapy.

Microwave (MW) irradiation, characterized by its low tissue invasiveness and high tissue penetrability, stands as an ideal external stimulus for cancer therapy.^[^ ^280^ ^]^ However, the energy in the MW (400–2500 MHz) is relatively low (10^−3^ eV), thus ablating tumors with MW irradiation have a limited diameter of 3 cm due to the restriction of heat transmission diameter.^[^ ^281^ ^]^ To address the above problems, some MW‐sensitive nanomaterials have been investigated to enhance MW‐mediated cancer therapy.^[^ ^280^ ^]^ For instance, Meng's group has revealed that liquid metal (LM) supernanoparticles (SNPs) activated by MW irradiation can generate ·OH and ·O_2_^−^, which are produced by the electron transfer from Ga to the water and oxygen adsorbed in the mesopores of SNPs.^[^ ^282^ ^]^ Additionally, some novel nanomaterials have been also explored to generate ^1^O_2_ under MW irradiation, including Cu_2_ZnSnS_4_ nanocomposites,^[^ ^283^ ^]^ g‐C_3_N_4_ QDs,^[^ ^284^ ^]^ TiMOF@Ni NPs@covalent organic frameworks (COF),^[^ ^285^ ^]^ Bi‐Mn‐MOF@COF‐based BMCAP^[^ ^286^ ^]^ (Figure 8g). However, the mechanism of these nanomaterials generating ^1^O_2_ under MW remains unclear. One possible explanation^[^ ^285^ ^]^ is that they produce “hot spots” under MW irradiation, resulting in point defects or weak surface bonds, where the reaction substrate (H_2_O, O_2_, H_2_O_2_) is adsorbed to generate ROS.

Collectively, with rationally designed multifunctionality, enormous responsive nanomaterials have been developed for generating reactive oxidative species under different external stimuli to disrupt intracellular redox homeostasis, ultimately inducing apoptosis, autophagy, necrosis, and ferroptosis for cancer therapy. A large number of in vivo examples demonstrated that these types of stimuli‐responsive nanosystems have made outstanding contributions to improving the efficiency of ROS/RNS‐based therapy, driving collaborative treatment of cancer, and circumventing some drug resistance mechanisms. However, many questions need to be solved before their clinical applications, such as the poor controllability and low specificity of nanosystems, long‐term safety, and the power and accuracy of the external stimuli generator. Fortunately, several nanomedicines have been approved in clinic or clinical trials. For example, the iron oxide nanoparticle‐based nanomedicine (NanoTherm) won approval in Europe for the treatment of glioblastoma. A single‐arm study in two centers demonstrated that thermotherapy using intratumoral magnetic nanoparticles in conjunction with a reduced radiation dose is safe and effective and leads to longer OS‐2 compared to conventional therapies in the treatment of recurrent glioblastoma.^[^ ^287^ ^]^ The clinical pilot device study demonstrated that gold‐silica nanoparticles (AuroShell particles) designed to absorb near‐infrared light at wavelengths of high tissue transparency ‐mediated focal laser ablation was successfully achieved in 94% (15/16) of patients, with no significant difference in International Prostate Symptom Score or Sexual Health Inventory for Men observed after treatment, providing a highly localized light‐based strategy for the treatment of prostate cancer.^[^ ^288^ ^]^ HfO_2_ nanoparticles, which are activated by external‐beam radiotherapy to generate oxygen free radicals, in a phase 2–3 clinical trial exhibit promising results clinically in patients with locally advanced soft‐tissue sarcoma, without adding toxicity to the surrounding tissues.^[^ ^289^ ^]^ These external stimuli‐responsive nanosystems have shown great prospects for clinical translation.

#### 3.1.3 Responsive Nanomaterials that Regulating Endogenous Oxidation Signaling Pathways

In addition to exploring nanomaterials designed for the overproduction of reactive oxidative species beyond the toxic threshold in tumor tissues, the abrogation of oxidative equilibrium by using exogenous nanomaterials to regulate endogenous oxidation signaling pathways provides an alternative therapeutic approach.

##### Targeting ROS‐Generating Oxidases

Various xenobiotic, metabolic, and other physiological pathways are known to generate ROS. Several sources of ROS in cells and tissue have been identified, including mitochondrial electron transfer chain and enzymatic reaction of lipoxygenases, monoamine oxidases, xanthine oxidase, uncoupled NOS, NOX enzymes, and cytochrome P450 oxidases.^[^ ^290^ ^]^ Therefore, targeting these ROS‐generating oxidases through nanomaterials is an attractive modality to treat cancer.

**Figure 9:**
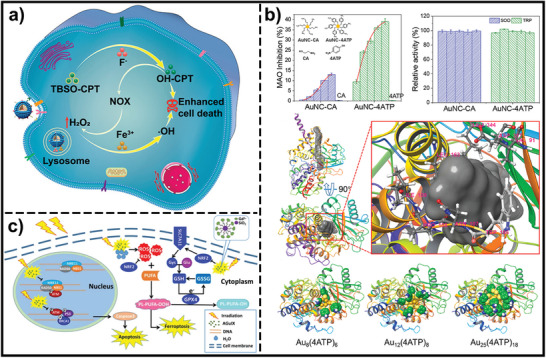
Schematic illustration of nanomaterials regulating endogenous oxidation substances. a) The release of OH‐CPT from the cleavage of TBSOCPT by fluoride ions activates NOX to upregulate intracellular H_2_O_2_ and amplify the iron‐induced Fenton reaction to achieve synergistic therapy. Reproduced with permission.^[^ ^295^ ^]^ Copyright 2022, American Chemical Society. b) Upper figure: MAO oxidase inhibitory ability of different concentrations of MAOI‐like AuNC, bottom figure: Computational analysis of MAOI activity of size‐dependent AuNCs. Reproduced with permission.^[^ ^305^ ^]^ Copyright 2021, Elsevier. c) AGuIX nanoparticles amplify the ferroptosis system by inhibiting the Nrf‐2‐GSH‐GPX4 signaling pathway. Reproduced with permission.^[^ ^312^ ^]^ Copyright 2022, Springer Nature.

NOX catalyzes the transfer of electrons from NADPH to molecular oxygen, resulting in the generation of O_2_∙^−^ and H_2_O_2_. The NOX family includes seven isoforms with different activation mechanisms: NOX1, NOX2, NOX3, and NOX5 produce ·O_2_^−^ while NOX4, DUOX1, and DUOX2 generate H_2_O_2_. NOX‐mediated ROS production plays a central role in various signaling pathways involved in the regulation of cell proliferation, differentiation, and apoptosis.^[^ ^291^ ^]^ Numerous findings highlighted that NOX‐induced ROS production provokes the acquisition of chemoresistance and contributes to cancer progression.^[^ ^292^ ^]^ Therefore, modulation of NOX expression, as well as their activity, is considered a new promising therapeutic approach for cancer treatment. There have been three attempts to target NOX‐mediated ROS production in cancer therapy. One methodology is using NADPH inhibitors or genetic silencing to suppress ROS‐triggered somatic mutations and abnormal signaling pathways involved in tumor development and angiogenesis.^[^ ^293^ ^]^ Unfortunately, this approach does not currently show significant improvement due to the lack of highly specific and validated inhibitors for different NOX enzymes. Nanocarriers could significantly improve the effect of the inhibitors. For example, to overcome resistance to radiotherapy, Zhu et al. have developed a bioactive and CD44 targeted hyaluronic acid nanoparticle encapsulated with a NOX inhibitor GKT831, which had stronger inhibitory effects on ROS generation and cell proliferation than GKT831 alone in cancer cells.^[^ ^294^ ^]^ Another methodology is activating the expression of NOXs, enhancing NOXs activity, or inducing exogenous NOX NADPH mimics. For example, camptothecin,^[^ ^295^ ^]^ cisplatin,^[^ ^84^, ^296^ ^]^ and Dox^[^ ^297^ ^]^ can activate the membrane‐spanning NOXs. A recent study showed that NADPH‐producing and DOX‐conjugating *Escherichia coli* (*E. coli*) exhibited enhanced antitumor efficacy.^[^ ^298^ ^]^ He et al. have developed a nanoamplifier for CDT by coupling photoresponsive conjugated polymer NPs with ferrocene and cisplatin prodrug.^[^ ^299^ ^]^ Zhang et al. have constructed a bifunctional biomimetic MOF‐F bioorthogonal nanoplatform by coating cancer cell membranes on an F^−^ absorbed MOF for prodrug activation and enhanced synergistic CDT.^[^ ^295^ ^]^ After entering cells, the acidic lyso/endosome environment can disrupt the pH‐sensitive metal–ligand bonds of MIL‐53, subsequently releasing F^−^ and Fe^3+^. The released F^−^ is able to initiate the desilylation reaction in situ to activate OH‐CPT. The generated OH‐CPT is found to activate NOX to replenish intracellular H_2_O_2_, which further intensifies Fenton reaction (**Figure** **9**a). In addition, some nanomaterials such as Pt NPs have NADH oxidase‐like activity. Wu et al. have reported a NADPH‐initiated cascade nanocatalytic system for ferroptotic tumor therapy by grafting Pt NPs onto MIL‐101(Fe) framework possessing NOX/SOD‐mimic activities and Fenton reaction capacity. The NOX nanozyme catalyzed NADPH oxidation to produce ∙OH/·O_2_^−^, which largely prevents the GSH regeneration and de‐activates glutathione peroxidase 4 (GPX4), promoting lipid peroxidation for ferroptotic cell death.^[^ ^300^ ^]^ Chong et al. have developed a cascade catalytic nanovaccine, metal‐polyphenol networks adsorbed on the model antigen ovalbumin templates, in which the “Fe‐GA catalytic unit” mimicked the ROS transfer process of NOX2.^[^ ^301^ ^]^

Monoamine oxidase A (MAOA) is a flavoprotein anchored to the mitochondrial outer membrane, that catalyzes the oxidative deamination of a number of biogenic and dietary amines to generate H_2_O_2_ as a by‐product in the process.^[^ ^302^ ^]^ Recent studies have indicated that MAOA either has altered expression levels or exerts a regulatory effect in a variety of cancer types such as prostate cancer.^[^ ^303^ ^]^ Therefore, suppressing MAOA/MAOB (isoenzymes) activity holds potential therapeutic prospects for tumor control.^[^ ^304^ ^]^ Recently, Wu et al. found that AuNCs modified with cysteamine or 4‐aminothiophenol, ≈1–3 nm in size, were found to have MAO inhibitor activity^[^ ^305^ ^]^ (Figure 9b). The possible mechanism is that the proper size of AuNCs with ligands containing amino groups can bind tightly with the entrance to active sites of MAO, blocking the enzyme‐substrate interaction.

##### Downregulating Nrf‐2

As discussed in Part 2, Nrf‐2 plays an important role in the regulation of cellular redox homeostasis.^[^ ^306^ ^]^ Nrf‐2 not only regulates the biosynthesis, utilization, and regeneration of three major antioxidant molecules, GSH, Trx, and NADPH, but also controls the production of ROS through mitochondria and NOXs.^[^ ^307^ ^]^ Nrf‐2 has multifaceted roles in cancer cells. Transient Nrf‐2 activation has protective roles against carcinogenesis and cancer development, while permanent activation of Nrf‐2 promotes malignant progression, chemo/radio resistance.^[^ ^308^ ^]^ Several studies have provided evidence that preventing the permanent activity of Nrf‐2 by peptide, small‐molecule inhibitors, and small interfering RNAs (siRNAs) renders cancer cells susceptible to apoptosis and sensitizes cancer cells to chemo/radiotherapeutics.^[^ ^309^ ^]^ Numerous nanocarriers have been designed to improve the efficacy in targeting the TME and the combined administration of Nrf‐2 modulators and conventional antineoplastic agents.^[^ ^310^ ^]^ Apart from using nanomaterials as carriers for Nrf‐2 modulators, recent studies demonstrated that several inorganic nanomaterials can directly downregulate Nrf‐2. Hsieh et al. found that zero‐valent iron nanoparticles enhanced GSK3β/β‐TrCP‐dependent degradation of Nrf‐2 through activation of the AMPK/mTOR signaling pathway, thereby triggering ferroptotic cell death through excessive oxidative stress and lipid peroxidation.^[^ ^311^ ^]^ Sun et al. designed the AGuIX nanoparticles that promoted not only radiation‐induced DNA damage and apoptosis, but also ferroptosis by Nrf‐2‐GSH‐GPX4‐dependent pathway after irradiation may regulate the anti‐ferroptosis system by inhibiting the Nrf‐2‐GSH‐GPX4 signaling pathway (Figure 9c), enabling radiosensitization of gadolinium‐based nanomaterials to kill triple‐negative breast cancer (TNBC) cells.^[^ ^312^ ^]^

In summary, redox signaling pathways play a vital role in regulating redox homeostasis. Redox signaling‐based nanomedicine approach has emerged as a new platform for cancer therapy, where responsive nanomaterials modulate the expression of ROS‐generating oxidases as well as their activity and downregulate Nrf‐2 to trigger excessive ROS formation. However, responsive nanomaterials regulating endogenous oxidation signaling pathways are rare. Moreover, the mechanism of nanomaterials regulating redox signaling is also fully understood. Therefore, a thorough understanding of nanomedicine‐regulated redox signaling would inspire researchers to design and develop more novel nanomaterials that could be used as an alternative strategy for the treatment of cancer, where angiogenesis plays a vital role. In addition, an aberrant redox signaling cascade can be caused by several exogenous and endogenous factors and remain incompletely understood. A complete understanding of redox signaling pathways influenced cancer progression and chemo‐or radio‐resistance would provide a guide for designing more nanosystems to regulate redox signaling pathways and improve cancer therapy.

### 3.2 Responsive Nanomaterials Inducing Reductive Damage

One characteristic of most cancer cells that distinguishes them from normal cells is that they exhibit higher ROS levels. However, to counteract excessive ROS production, some cancer cells and cancer stem cells constantly attempt to augment their antioxidant defense capacity, resulting in excessive production/accumulation of reducing equivalents, such as GSH, NADPH, NADH, and the free thiol group in the cysteine (Cys) residues of proteins, which drives the cells to reductive stress.^[^ ^313^ ^]^ Currently, most therapeutic strategies such as chemotherapy and radiotherapy are intended to kill cancer cells through the generation of ROS, however, these strategies can eventually facilitate the development of drug/radioresistance.^[^ ^314^ ^]^ Recent studies have revealed that excess reductive stress has similar deleterious effects as oxidative stress such as DNA damage, mitochondrial dysfunctions, reductions in cellular metabolism, and cell death via apoptosis or endoplasmic reticulum stress, thus leading to cellular dysfunction.^[^ ^315^ ^]^ For example, chronic reductive stress not only perturbs ROS signaling functions, but also promotes ROS production, as the reduced elements of redox pairs or their antioxidant enzymes using them as coenzymes partially reduce O_2_ forming ·O_2_^−^.^[^ ^315b^ ^]^ Moreover, at the endoplasmic reticulum (ER), protein disulfide bonds are inappropriately formed under reductive stress, resulting in misfolding or aggregation of proteins^[^ ^315^, ^316^ ^]^ suggesting that reductive stress might provide an opportunity to prevent or treat cancer.^[^ ^315^, ^317^ ^]^ Nanotechnology can also alter reductive stress in a tumor or its environment to suppress the proliferation and/or induce cell death (apoptosis) in tumor cells by delivering or generating additional reduction equivalents as well as reducing antioxidant mechanisms to indirectly cause oxidative damage. In this section, a series of responsive nanomaterials that cause reductive damage are summarized, including using responsive nanosystems for delivering antioxidants, producing high levels of reducing equivalents in the cellular environment or suppressing reductive stress to indirectly promote oxidative damage.

#### 3.2.1 Responsive Nanocarriers for Delivery of Antioxidants

Numerous studies from preclinical to clinical models have suggested that natural or artificial antioxidants, including resveratrol, curcumin, celastrol, ascorbic acid, etc., provide a protective effect in the development of cancer or an anti‐tumor effect.^[^ ^315b^ ^]^ Recent study demonstrated that the treatment of cancer cells with antioxidants under hypoxic conditions produces high levels of NAD(P)H before cell death, resulting in reductive stress instead of oxidative stress.^[^ ^318^ ^]^ Based on these findings, several responsive nanocarriers have been designed to deliver antioxidants into the cancer cells to further strengthen the degrees of reducing stress.^[^ ^319^ ^]^ For example, ascorbic acid (Vitamin C, AA for short) serves as an optimal reducing agent in human fluids, but its low transmembrane efficiency critically limits its antioxidative function within the cells. To address this problem, Tang et al. have designed poly(2‐vinylpyridine)‐polyethylene glycol‐folic acid functionalized core‐shell nanostructure of CdTe quantum dots with mesoporous silica coating, which can be used as a pH‐sensitive multifunctional drug nanocarrier with controllable release of AA under hypoxic environment.^[^ ^320^ ^]^ AA was found to enhance the accumulation of reductive species, break the redox balance in HepG2 cells, and trigger the reductive stress to induce the apoptotic signaling pathway, thereby dealing minimum damage to normal tissues. Zhao et al. have constructed the reductive lipid NPs loaded with vinorelbine and reductive component Vitamin E, which can effectively reduce oxidative stress and significantly outperform free vinorelbine in preventing tumor progression.^[^ ^321^ ^]^

#### 3.2.2 Responsive Nanosystems Generating Reducing Equivalents

Given that reductive stress can be useful against cancer, responsive nanosystems generating reducing equivalents to induce reductive stress in tumors or their environment without affecting other tissues have gradually been developed for cancer therapy. In this section, reductive stress‐inducing nanosystems are classified according to reducing equivalents, such as hydrogen sulfide, hydrogen selenium, hydrogen gas, etc. (**Table** **3**).

**Table 3:** Summary of the representative nanomaterials that generate reduction substances.

Material	Reduction substance type	Reduction substance generating substance	Other treatment types	Therapy mechanism	Comment	Ref.
CY‐PSD	H_2_S	Polysulfide	–	Mitochondrial dysfunction	The released H_2_S by CY‐PSD mediates mitochondrial dysfunction and oxidative stress inhibition, leading to 63% tumor suppression	[325a]
Pry‐Ps@CP‐PEG	H_2_S	Polysulfide	PTT	Mitochondrial dysfunction	The released H_2_S causes mitochondrial dysfunction and anti‐inflammatory effects, thereby inhibiting tumor growth	[325b]
Cur‐coloaded mPEG‐PLGA	H_2_S	SH‐aspirin	–	Mitochondrial dysfunction	The combination of SH‐ASA and Cur‐induced apoptosis through the mitochondrial‐based signaling pathway	[325c]
PEG‐PADT	H_2_S	ADT	–	Mitochondrial dysfunction	The released H_2_S by micelles exerts the anti‐proliferative effect in‐vitro and in‐vitro	[325d]
ZnS@ZIF‐8/ICG/TPZ	H_2_S	ZnS	Chemotherapy, PDT	Catalase inhibition	The combined effects of ROS, H_2_S, and hypoxia‐activated TPZ enabled more effective and lasting anticancer treatment	[326a]
DOX‐ZnS@SiO_2_	H_2_S	ZnS	Chemotherapy	Catalase inhibition	ZnS generated H_2_S in acidic TME, which inhibits CAT activity and thus increases ROS production.	[326b]
NP1	H_2_S	H_2_S polymetric donor P2	PTT	COX IV inhibition	The generated H_2_S by NP1 suppresses mitochondrial respiration by inhibiting the expression of COX IV	[326d]
PEG‐FeSe_2_	H_2_Se	FeSe_2_	PTT	Mitochondrial dysfunction	H_2_Se caused the downregulation of the HMGB1 protein, which is sensitive to oxidative stress in 4T1 tumor cells	[335]
PdH‐MOF	H_2_	PdH		Mitochondrial dysfunction	The PdH‐MOF exhibited a high hydrogen loading capacity, sustained hydrogen release profile, high tumor targeting ability, high photothermal effect	[339b]
DOX+MBN	H_2_	MBN	Chemotherapy	Inhibiting aerobic respiration of gastric cells	2D MgB_2_ nanosheet as acid‐responsive hydrogen‐releasing prodrug realizes hydrogenochemotherapy	[340]
AB@MPDA‐PEG	H_2_	AB	PTT	Mitochondrial dysfunction	Combination of hydrogen and photothermal therapies	[341a]
AB/DOX@HMPDA‐PEG	H_2_	AB	Chemotherapy	Mitochondrial dysfunction	AB releases H_2_ controllably in an acidic TME, which blocks mitochondrial respiration to reduce ATP production and enhances chemotherapy	[341b]
HC‐AB NPs	H_2_	AB	PTT	Mitochondrial dysfunction	HC‐AB NPs are stimulated by acidic TME to release H_2_, achieving synergetic hydrogenothermal therapy combined with NIR‐II PTT	[341d]
mPDAB	H_2_	AB	PTT	Mitochondrial dysfunction	The mPDAB eliminates the primary tumors, while also restraining the outgrowth of distant dormant tumors	[341e]
AB@MSN	H_2_	AB	–	Mitochondrial dysfunction	AB@MSN exhibits a high H_2_ loading capacity (130.6 mg/g, more than 1370 times higher than that of the traditional H_2_@liposome)	[341f]
Nano‐CaH_2_	H_2_	CaH_2_	Immunotherapy	Mitochondrial dysfunction	CaH_2_ reacts with H_2_O to simultaneously achieve hydrogen therapy and Ca^2+^‐mediated immunotherapy	[343]
PdH_0.2_	H_2_	PdH_0.2_	PTT	Mitochondrial dysfunction	H_2_ was stabilized in the lattice of Pd crystals, and the hydrogenation led to an enhancement of near‐infrared absorption (PTT)	[344]
MgG	H_2_	Mg	Immunotherapy	Mitochondrial dysfunction	H_2_‐induced mitochondrial dysfunction and disruption of redox homeostasis combined with neutralization of the acidic TME ultimately realize tumor therapy in patient xenografts and mouse&rabbit models	[345]

Abbreviations: PSD, Polysulfide; ADT, Anethole ditholethione; COX IV, Cytochrome c oxidase IV; ASA, Aspirin.

##### Generation of H_2_S

**Figure 10:**
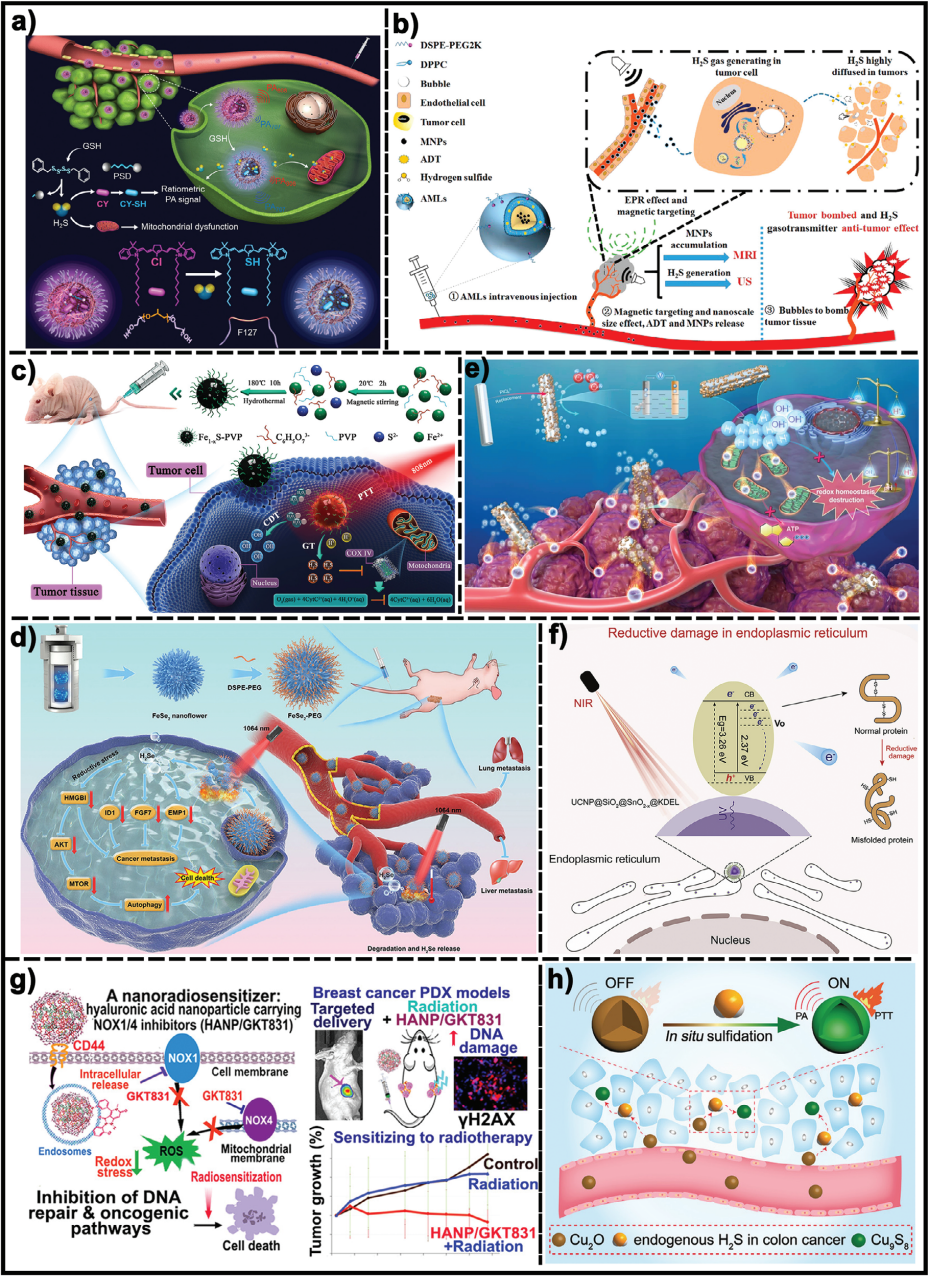
Schematic illustration of nanomaterials inducing reduction imbalances. a) Ratiometric photoacoustic (PA) monitored TME‐initiated H_2_S therapy for TNBC. Reproduced with permission.^[^ ^325a^ ^]^ Copyright 2020, Wiley‐VCH. b) Nano to the micro conversion of AMLs for US/MR dual‐modal imaging and the spatiotemporal‐bombed combination tumor accurate therapy. Reproduced with permission.^[^ ^326e^ ^]^ Copyright 2017, American Chemical Society. c) H_2_S released from Fe_1−x_S‐PVP under acidic TME inhibits COX IV activity to control tumor growth. Reproduced with permission.^[^ ^326c^ ^]^ Copyright 2021, Wiley‐VCH. d) Tumor accumulation of photoreleased H_2_Se from FeSe_2_‐PEG nanoflowers induces cell death and inhibits liver and lung metastasis. Reproduced with permission.^[^ ^335^ ^]^ Copyright 2021, Elsevier. e) The preparation of MgG for TME modulation and enhanced exogenous hydrogen therapy. Reproduced with permission.^[^ ^345^ ^]^ Copyright 2022, Springer Nature. f) The light‐triggered electrons by UCSNKNPs attack protein disulfide bonds, leading to structural disruption of ER, thereby enabling reductive damage cancer therapy. Reproduced with permission.^[^ ^348^ ^]^ Copyright 2022, Elsevier. g) HANP/GKT831 reduces ROS generation by inhibiting NOX1 and NOX4, resulting in reductive stress for the treatment of PDX tumors. Reproduced with permission.^[^ ^294^ ^]^ Copyright 2022, American Chemical Society. h) Representation of the in situ reaction of Cu_2_O and endogenous H_2_S triggered PA imaging and PTT for colon cancer. Reproduced with permission.^[^ ^359^ ^]^ Copyright 2018, Wiley‐VCH.

Hydrogen sulfide (H_2_S), an endogenous gasotransmitter, is derived from Cys and/or homocysteine in reactions catalyzed by cystathionine γ‐lyase and cystathionine β‐synthase, or from 3‐mercaptopyuvate in a reaction catalyzed by 3‐mercaptopyruvate sulfurtransferase. H_2_S influences a broad range of physiological functions via post‐translational modification of the thiol redox proteome, converting Cys thiols to persulfides.^[^ ^322^ ^]^ Recent studies have revealed that, at higher concentrations, H_2_S induces reductive stress by inhibiting the electron transfer chain, causing a reductive shift in the NAD^+^/NADH, FAD/FADH_2_, and CoQ/CoQH_2_ redox couples.^[^ ^323^ ^]^ The H_2_S‐induced redox changes might fan out from the mitochondrion to other compartments such as cytoplasm. Moreover, higher concentrations of H_2_S can also increase metabolic acid–lactate production and impair the pH regulatory system, leading to cell apoptosis.^[^ ^324^ ^]^ These findings implicate that producing exogenous high levels of H_2_S may represent a potential selective anti‐cancer therapeutic strategy. However, progress in the development of H_2_S therapy is hindered by the limited availability of donors and their poor stability during blood circulation, as well as their lack of specificity for targeting tumors.^[^ ^325^ ^]^ To achieve precisely controllable H_2_S therapy, various H_2_S‐generating nanosystems, including those delivering H_2_S donors such as PSD,^[^ ^325a,b^ ^]^ SH‐ASA,^[^ ^325c^ ^]^ and anethole dithiothione group^[^ ^325d^ ^]^ (Table 3). For example, Li et al. have developed a polysulfide‐based theranostics approach for detecting and treating TNBC using cyanines as a ratiometric PA probe^[^ ^325a^ ^]^ (**Figure** **10**a). Polysulfide within the nanosystem quickly released H_2_S to a therapeutic concentration, which is real‐time sensed by cyanines based on ratiometric PA signals (PA_707_/PA_808_). This polysulfide‐based theranostics exhibited real‐time H_2_S monitoring and tumor pinpointing. In addition, the unique characteristics of tumor cells and external stimuli have also been utilized for the localized generation of H_2_S.^[^ ^326^ ^]^ For instance, Cen et al. have synthesized ZnS@BSA nanoclusters via a self‐assembly approach for enhanced immunotherapy. Upon acidic TME, zinc and sulfur ions were released from the nanoclusters. The released zinc ions improved the catalytic enzyme activity of cGAS and significantly enhanced cGAS/STING signals, while sulfur ions react with intracellular hydrogen ions to generate intracellular H_2_S that specifically inhibit the activity of catalase and thus lead to the accumulation of ROS in tumor cells.^[^ ^327^ ^]^ Gu's group has reported a simple yet stimuli‐responsive magnetic nanoliposome delivery system, which consists of small superparamagnetic nanoparticles encapsulated in the aqueous core of the liposomes and hydrophobic ADT doped in the phospholipid shell.^[^ ^326e^ ^]^ As shown in Figure 10b, the magnetic nanoliposome delivery system preferentially targets the tumor tissue when spatiotemporally navigated by an external magnetic field, and the released anethole ditholethione continuously generated large numbers of microsized H_2_S bubbles via an enzymatic trigger. Such a nano‐ to microsize change enables anethole ditholethione to be localized within the TME, microbubble‐enhanced US imaging, and spatiotemporal‐bombed combination tumor accurate therapy. Yang et al. have prepared polyvinyl pyrrolidone‐modified multifunctional iron sulfide nanoparticles with high PT conversion efficiency to enhance the CDT and H_2_S‐mediated therapy.^[^ ^326c^ ^]^ In a weakly acidic tumor environment, the NPs can release S^2−^ and Fe^2+^. The released S^2−^ combines with H^+^ to in situ produce H_2_S gas, which suppresses the activity of the enzyme cytochrome c oxidase in cancer cells. The PT effect further facilitates the Fe^2+^‐mediated Fenton reaction to generate abundant ∙OH radicals for tumor ablation (Figure 10c).

##### Generation of H_2_Se

A balance of selenium (Se) that forms part of the selenoaminoacids, selenomethionine, and selenocysteine is needed for several biological functions in the human body, and an excess and/or insufficient intake can result in adverse health effects.^[^ ^328^ ^]^ In addition, Se and Se‐containing compounds have been proven to be selectively absorbed by cancer cells and have been shown to function as antioxidant and prooxidant agents, providing the basis for their potential applications in cancer prevention and treatment.^[^ ^329^ ^]^ The mechanisms of action of Se and Se‐containing compounds are very diverse, including GPX‐like activity, reduction of oxidative stress, cytotoxic activity, and the triggering of apoptotic events, etc. Several studies have demonstrated that some Se‐containing compounds such as aryl diselenides induce cytotoxicity in cancer cells by causing reductive stress rather than oxidative stress.^[^ ^330^ ^]^ H_2_Se, a highly reactive molecule with reducing properties, is a shared metabolite of dietary Se‐compounds and formed from Na_2_SeO_3_ via selenodiglutathione through reduction by thiols and NADPH‐dependent reductases and released from selenocysteine by lyase action.^[^ ^331^ ^]^ Under hypoxic conditions, H_2_Se accumulation can lead to reductive stress.^[^ ^332^ ^]^ However, the half‐life for the oxidation of H_2_Se to elemental Se in air‐saturated water at pH 7 is quite short (≈30 s^[^ ^333^ ^]^), making the controllable production of H_2_Se technically challenging. Although recently several new small‐molecule H_2_Se donors have been developed via bond hydrolysis or by the ─SH moiety of Cys to achieve continuous release of the unstable biomolecule under physiologically relevant conditions,^[^ ^334^ ^]^ they still suffer from the relatively slow H_2_Se release, poor long‐circulation ability and potential toxicity caused by non‐specific biodistribution. To address these concerns, Lu's group^[^ ^335^ ^]^ has developed polyethylene glycol‐modified ferrous selenide nanoflowers with NIR‐II photoactivatable H_2_Se generation ability, which disintegrated and released H_2_Se upon the irradiation of 1064 nm laser (Figure 10d). This PTT‐reduction gas synergistic therapy effectively inhibited liver and lung metastasis in the 4T1 mouse model, offering a promising strategy for the design of H_2_Se‐nanomaterials that trigger reduction stress.

##### Generation of H_2_

Recent studies have found that H_2_ can interact with and scavenge free radicals, including ·OH and ONOO^−^ anions,^[^ ^336^ ^]^ disrupt intracellular redox balance, activate endogenous antioxidant enzymes, induce apoptosis and pyroptosis in tumor cells, etc.^[^ ^336^, ^337^ ^]^ These features make H_2_ a highly promising strategy against cancer.^[^ ^337^, ^338^ ^]^ Various H_2_ carriers including Pd‐based nanocrystals,^[^ ^339^ ^]^ MgB_2_ nanosheets,^[^ ^340^ ^]^ ammonia borane‐loaded mesoporous silica NPs,^[^ ^341^ ^]^ Fe NPs,^[^ ^342^ ^]^ and CaH_2_^[^ ^343^ ^]^ were fabricated to achieve the storage, targeted delivery and controlled release of H_2_ (Table 3). They are roughly divided into two strategies: 1) Nanocarrier‐mediated H_2_ delivery: For instance, He et al. have synthesized a 2D MgB_2_ nanosheet as a new type of acid‐responsive hydrogen‐releasing prodrug, realizing hydrogenochemotherapy by the combination of facile oral administration of polyvinylpyrrolidone‐encapsulating MgB_2_ nanosheet pills with routine intravenous injection of Dox.^[^ ^340^ ^]^ 2) In situ production of H_2_ by nanogenerators in response to exogenous or endogenous stimuli: He's group^[^ ^344^ ^]^ has designed a cubic PdH_0.2_ nanocrystal and used it for exogenous H_2_ release through NIR light. As shown in Figure 8e, the synthesized PdH_0.2_ nanocrystals have exhibited high intratumoral accumulation capability, and their hydrogenation results in the significant enhancement of NIR absorption, which enables the PAI‐guided release of bio‐reductive hydrogen as well as PTT. Wang et al. have designed a smart PdH@MnO_2_/Ce6@HA yolk–shell nanoplatform for synergistic H_2_/PTT/PDT in combating cancer.^[^ ^339a^ ^]^ Liu et al. have designed a micro‐galvanic cell by in situ reduction of a small amount of Pt on the surface of Mg rods (Figure 10e). The obtained Mg‐based galvanic cell can be etched by water to allow the continuous generation of H_2_ and Mg(OH)_2_. After implanting MgG rods into tumors, the continuous generation of H_2_ could induce mitochondrial dysfunction and disruption of intracellular redox homeostasis, while the byproduct Mg(OH)_2_ could neutralize the acidic TME.^[^ ^345^ ^]^ Both strategies can significantly enhance the targeted accumulation of H_2_ and accelerate therapeutic effects.

##### Cellular Compartments‐Targeted Reductive Damage

The redox potential is different within various intracellular subcellular organelles. For example, mitochondria require a more reduced potential and the reported range of GSH/GSSG is from 20:1 to 40:1; while the endoplasmic reticulum (ER) needs a more oxidative environment for the folding of secretory proteins through the formation of disulfide bridges and the ratio of GSH/GSSG varies from 1:1 to 3:1.^[^ ^346^ ^]^ Therefore, the ER is susceptible to reducing damage caused by excessive electron pressure, which will disrupt the protein folding state within the ER by breaking disulfide bonds in proteins in an oxidative damage‐independent manner.^[^ ^347^ ^]^ For this purpose, Bu's group^[^ ^348^ ^]^ has designed a NIR‐responsive, ER‐targeted electron donor by using Yb^3+^ and Tm^3+^ co‐doped UCNPs as the light converters and oxygen vacancies of self‐doped SnO_2−x_ NPs shell as sacrificial electron donors. As shown in Figure 10f, the generated ultraviolet fluorescence from the UCNPs under NIR irradiation excites the separation of electron‐hole pairs in the SnO_2−x_ shell. Photogenerated electrons abort the protein folding process in the ER via a reductive manner, achieving upregulation of the transcription factor Nrf‐2, elevated reductive equivalents, and effective cancer therapeutic outcomes. Notably, this strategy is by the utility of reductive damage by harnessing, rather than antagonizing, the intrinsic antioxidant defenses of cancer cells. This unique electronic interference therapy may have broad indications for other intractable diseases.

In summary, with the advent of new technologies that measure reductive stress and regulate reductive stress, emerging evidence suggests that an overly reductive environment also results in profound cellular damage and dysfunction. According to the abovementioned research data, responsive nanomaterials that can deliver or generate reducing equivalents could disrupt the redox balance in cancer cells and ultimately induce the apoptotic signaling pathway, suggesting that manipulating reductive stress may represent a new therapeutic avenue for cancers defined by specific genotypes. However, responsive nanomaterials targeting reductive stress are rare, and this strategy is expected to receive extensive attention and research. Moreover, further study is needed to fully understand the role of reductive stress induced by nanomaterials on cell metabolism and its impact on protein function and the potential compartmentalization, which would help translate this strategy into new cancer therapies.

#### 3.2.3 Responsive Nanosystems Suppressing Reductive Stress to Indirectly Promote Oxidative Damage

As described earlier, cancer cells produce excessive levels of antioxidants and high concentrations of reduced nucleotide co‐factors to overcome oxidative damage. Therefore, inhibiting intracellular antioxidant systems (e.g., antioxidant molecules (GSH and NADPH), reducing cofactors (NADH and FADH), and antioxidant enzymes (HO‐1) is a potential strategy to enhance the efficiency of ROS‐mediated cancer therapy.

NADPH is well known as an essential electron donor and an indispensable cofactor that is used for transferring and reserving reduction potential for numerous anabolic reactions.^[^ ^349^ ^]^ A growing body of evidence has shown that with the metabolic reprogramming of NADPH, cancer cells increase the demand for NADPH for power redox defense and anabolic reactions to sustain their rapid growth.^[^ ^350^ ^]^ It has been reported that selectively lowering levels of NAD(H) and NADPH by targeting NAD(P)H‐synthesis pathways or NAD(P)H‐related enzymes impairs tumor metabolism and induces excessive ROS accumulation, leading to cell toxicity and death.^[^ ^351^ ^]^ For example, NOXs catalyze the conversion of NADPH and oxygen into NADP^+^ and ROS, and inhibition of the NOX1 and NOX4 signaling pathways has great potential in tumor treatment.^[^ ^293c^ ^]^ Recently, Yang's group^[^ ^294^ ^]^ has reported the preparation of HANP nanoparticles by self‐assembly and encapsulation of the NOX1/4 dual inhibitor GKT831 (HANP/GKT831) (Figure 10g). Compared to GKT831 alone, HANP‐encapsulated GKT831 showed robust inhibition of ROS production and cell growth in the human cancer cell lines and the breast cancer patient‐derived xenograft models. On the contrary, recent studies have demonstrated that excess accumulation of NADH leads to disruption of de novo lipid, amino acid, and nucleotide biosynthesis due to the decreased electron acceptors.^[^ ^352^ ^]^ Recently, Zhang et al. reported a selective H_2_O_2_ electrochemical nanosensor, which is prepared by electrodeposition of Prussian blue (PB) and polyethylenedioxythiophene (PEDOT) onto carbon fiber nanoelectrode. With this nanosensor, they find that the level of intracellular H_2_O_2_ increases with NADH treatment, and intratumoral injection of high‐dose of NADH can inhibit tumor growth in mice, providing a potential therapeutic strategy for cancer therapy.^[^ ^353^ ^]^ Proposing the concept of hybrid bacteria, Sun et al. conjugated Dox onto the surface of a non‐pathogenic *E. coli* strain overexpressing glucose dehydrogenase through a stable linker of amide bonds. More amounts of NADPH are produced in tumor sites by glucose dehydrogenase within *E. coli*, promoting the generation of toxic ROS within the tumor. The toxic ROS generation further amplifies the therapeutic effect of DOX‐mediated chemotherapy and immunotherapy.^[^ ^298^ ^]^

In addition to NAD(P)H, GSH is also overproduced in tumor cells (≈1–10 mm, 4 times greater than healthy cells) to counteract the comparatively raised H_2_O_2_ level,^[^ ^354^ ^]^ providing tumor cells with robust resistance to ROS‐mediated treatments. To boost the efficiency of ROS‐mediated cancer therapy, numerous GSH‐consumption nanosystems have been developed, which can be divided into two strategies: depletion of GSH^[^ ^355^ ^]^ or reduction of GSH synthesis.^[^ ^355^, ^356^ ^]^ To deplete GSH, Tan et al. have utilized the MnO_2_ nanosheet system to enhance the chlorin e6‐mediated PDT effect. In this system, MnO_2_ nanosheets reduced the cellular levels of GSH.^[^ ^357^ ^]^ Wang et al. synthesized a nanosized copper‐based MOF through one‐step self‐assembly between copper ions and the catechol ligand, which could oxidize GSH into GSSG and simultaneously disassemble due to the reduction of coordinated copper. Subsequently, the released catechol ligands and Cu^+^ promoted the production of intracellular ROS.^[^ ^358^ ^]^ Yang's group^[^ ^359^ ^]^ creatively discovered that the strong binding ability of cuprous oxide (Cu_2_O) to endogenous H_2_S in colon tumor sites is beneficial for tumor elimination (Figure 10h). After entering the colon tumor site, Cu_2_O reacts with endogenous H_2_S to form copper sulfide, which exhibits NIR absorption and can be used for PA imaging and PTT. For reducing GSH synthesis, Chen et al. reported salicylazosulfapyridine (SASP)/ZnO nanoparticles (NPs) to enhance the PDT effect of ZnO NPs by impairing GSH synthesis via SASP‐induced inhibition of the cystine/glutamate antiporter.^[^ ^360^ ^]^

In addition, since the cellular antioxidant defense mechanism is highly complex, simply decreasing the level of a single antioxidant molecule may have a limited impact on improving the efficiency of ROS‐mediated cancer therapy. Zhong et al. developed a versatile PDT nanosensitizer containing GSH inhibitor l‐buthionine sulfoximine and the HO‐1 inhibitor protoporphyrin zinc(II) to suppress the innate antioxidant defense system of cancer cells in a two‐pronged manner.^[^ ^361^ ^]^ The Trx/TrxRs system,^[^ ^362^ ^]^ which mitigates ROS stress through thiol and selenol groups, is another pivotal system. Cys is crucial for maintaining the TXN oxidation‐reduction systems.^[^ ^363^ ^]^ Based on this, Zhang's group^[^ ^364^ ^]^ has constructed a nanoplatform FePt@SiO_2_ NPs (sSFP). After the nanoplatform reached the tumor site, FePt initiated the Fenton reaction, causing an increase in intracellular free radicals (Ferroptosis). Furthermore, siRNA on the material interfered with xCT expression, blocking Cys uptake, and depleting intracellular Cys. Cys depletion disrupted the oxidation‐reduction system, amplifying the ferroptosis effect on the tumor. Detection of GSH and TXN proteins in cells and tumor tissues showed that the nanoplatform simultaneously disrupted both the GSH oxidation‐reduction system and the TXN oxidation‐reduction system. In a separate study, Cai et al.^[^ ^365^ ^]^ loaded Se nanoparticles onto natural β‐glucan nanotubes to prepare BFP‐Se nanomaterials. Metabolomics revealed that BFP‐Se‐related metabolic reactions primarily pertained to oxidative stress in liver cancer cells. Similarly, Western blot experiments demonstrated that BFP‐Se‐depleted intracellular GSH, inhibited the expression of Txnip/Trx and Nrf‐2/GPX4‐related antioxidant systems, ultimately inducing apoptosis and ferroptosis in liver cells. These studies offer valuable insights into the design of nanomaterials capable of simultaneously disrupting the oxidation‐reduction balance from the source.

### 3.3 Responsive Nanosystems for Boosting ROS/RNS Production and Inhibiting Antioxidant Mechanisms

As discussed in Part 3.1, the ROS/RNS‐dependent strategies can cause tumor cell death, and regulate immunogenicity through ICD for immunotherapy. However, considering intricate redox homeostasis in the TME and the heightened ROS elimination due to elevated antioxidant mechanisms, the ROS/RNS‐dependent strategies are still restricted by low ROS production efficiency, the short lifespan of ROS, and insufficient immune activation. The challenges lie in simultaneously reinforcing ROS/RNS production and reducing their expenditure via modulating antioxidant mechanisms. To address these challenges, some intelligent nanosystems have been developed to produce sufficient ROS/RNS, combined with the depletion of accumulated reducing substances (such as GSH) or the inhibition of over‐expressed antioxidant enzymes like catalase.

GSH, as the major cellular antioxidant, plays an important role in maintaining the balance between oxidation and antioxidation. The GSH concentration in cancer cells can reach as high as 10 mm, at least fourfold that in normal cells, which scavenge excessive ROS. Depleting intracellular GSH breaks the redox homeostasis and causes ROS accumulation, thus boosting the antitumor efficiency of ROS/RNS‐based cancer therapies. Therefore, to address these challenges, various functional nanosystems combining GSH‐consuming ability and ROS/RNS‐producing ability have been proposed to magnify oxidative stress in cancer cells and improve the outcomes of ROS/RNS‐based therapy^[^ ^356^, ^366^ ^]^ (**Table** **4**) These systems can be divided into two strategies: 1) Consuming existing GSH within cancer cells by oxidizing GSH or directly reacting with the thiol group of GSH. For example, some inorganic nanomaterials such as platinum (Pt),^[^ ^333^ ^]^ copper (Cu),^[^ ^367^ ^]^ gold (Au),^[^ ^368^ ^]^ manganese dioxide (MnO_2_),^[^ ^369^ ^]^ etc., and some organic molecule‐based nanosystem such as 5‐norbornene‐2‐carbaldehyde,^[^ ^370^ ^]^ hemin^[^ ^371^ ^]^ etc., are capable of interacting with GSH and effectively deplete intracellular GSH in cancer cells. Fan et al. designed an intelligent nanoparticle constructed by synthesizing tetrasulfide bond‐doped mesoporous silica nanoparticles, followed by conjugating GOx on the surface and loading l‐Arg into the mesopores. This nanoparticle possesses GSH depletion, H_2_O_2_ self‐supply, and l‐Arg loading properties for multimodal cancer therapy.^[^ ^348^ ^]^ 2) Inhibiting GSH biosynthesis from the upstream pathway. Huang et al. construct a homotypic cancer cell membrane‐camouflaged iron‐small interfering RNA nanohybrid. The SLC7A11‐targeted siRNA in the nanohybrid inhibits the biosynthesis of GSH by cutting off the supply of intracellular Cys, resulting in the inactivation of GPX4 and the accumulation of ROS generated from the Fenton reaction induced by iron. Both effects further increase the accretion of lipid peroxides to enhance iron‐induced ferroptosis.^[^ ^372^ ^]^

**Table 4:** Summary of the representative nanomaterials that simultaneously generate free radicals/RNS with GSH depletion.

Materials	Free radicals/RNS type	Free radicals/RNS generating element/material	GSH depleting element/material	Other treatment type	Comment	References
FeCysPW@ZIF‐82@CAT Dz	·OH	Fe	imidazole ligand	gene‐silencing	Degraded Zn^2+^ assists CAT Dz to cleave CAT mRNA to accumulate H_2_O_2_, and the dissociated electrophilic ligand depletes intracellular GSH, thereby enabling hypoxic tumor therapy under FeCysPW‐mediated CDT	[383]
UCNPs@Cu–Cys–GOx	·OH	Cu^+^ (CDT)	Cu^2+^	Ferroptosis, Starvation therapy, Immunotherapy	GOx initiates starvation therapy to amplify Cu^+^‐mediated CDT, while the oxidized Cu^2+^ consumes GSH to amplify CDT and ferroptosis. The produced oxidative stress reverses the immunosuppressive TME, binding α‐PD‐L1 for immunotherapy	[384]
GOx@[Cu(tz)]	·OH	Cu ions (CDT, PDT)	Cu^2+^	PDT; GOx; Cuproptosis	GSH stimulates GOx@[Cu(tz)] to turn on GOx starvation therapy, increased H_2_O_2_ enhances hypoxic PDT (type‐I) and CDT effects and releases Cu^+^ leading to cuproptosis	[385]
TP/2‐DG @HMnO_2_@HA	·OH	Mn^2+^	HMnO_2_, Triptolide	Starvation therapy	HMnO_2_ consumes GSH to generate Mn^2+^ to initiate CDT, and the released TP and 2‐DG block the generation of Nrf‐2 and ATP, respectively, so as to cut off the endogenous GSH supply and amplify the oxidative damage by CDT	[386]
MoWODH NCs	·OH	Mo^4+^, W^5+^	Mo^6+^, W^6+^	PTT, Chemo	Mo^4+^ and W^5+^ initiate CDT, and the oxidized Mo^6+^ and W^6+^ reduce GSH; they combine MoWO with up to 45.5% NIR‐II region PTT efficiency and chemotherapy of Dox to achieve tumor suppression	[387]
HXV_2_O_5_	·OH	multivalent V (CDT, SDT)	multivalent V	–	multivalent V elements simultaneously initiate CDT (low‐valence V) and GSH depletion (high‐valence V), combining the SDT effect of V to triple oxidative stress	[388]
H@Gd‐NCPs	·OH	Gd (Radiotherapy)	Hemin	Immunotherapy	Gd in H@Gd‐NCPs sensitizes X‐rays to generate ROS, while Hemin enhances peroxidase‐like properties to deplete GSH. This oxidative stress process induces ICD, combined with immune checkpoint blockade therapy to achieve primary, distant, and metastatic tumor treatment	[389]
Au–TiO_2_	·OH	Au–TiO_2_ (Radiotherapy)	Au–TiO_2_ (Radiotherapy)	–	The Schottky heterojunction formed by AuNR and TiO_2_ can not only consume GSH but generate ·OH under X‐rays, thus realizing the treatment of subcutaneous and orthotopic tumor	[390]
Pd SAzyme	·OH	POD‐like nanozyme	GSHOx‐like nanozyme	Ferroptosis	Pd SAzyme's mimetic POD and GSHOx enzymes catalyze ROS generation and GSH consumption, resulting in a large accumulation of LPO to initiate ferroptosis	[391]
p–n‐CD@CCM	^1^O_2_	p–n‐CD (SDT)	Photogenerated holes	–	CD of the p‐n junction inhibits recombination of the e^−^‐h^+^ pair to mediate ^1^O_2_ generation (e^−^) and GSH depletion (h^+^) under US irradiation	[392]
HSA‐Ce6‐IrO_2_	^1^O_2_	Ce6 (SDT)	Ir^4+^	Ferroptosis	Ce6 and IrO_2_ generate ^1^O_2_ through SDT, which promotes LPO accumulation to induce ferroptosis; the high‐valent Ir(IV) reduces GSH to inhibit GPX4, thus enhancing the ferroptosis	[393]
Ce6‐PEG‐HKN15	^1^O_2_+ ·OH	Ce6 (PDT); Fe^2+^ (CDT)	Fe^3+^	PDT, Ferroptosis (endogenous)	Ce6 generates ^1^O_2_ through PDT to destroy the intracellular iron pool, and the released Fe^3+^ consumes GSH to generate Fe^2+^, thereby initiating CDT to generate ·OH. These radicals oxidize PUFA to induce ferroptosis	[394]
CPMNS	^1^O_2_+·OH	Co^2+^ (CDT), Mo^5+^	Mo^5+^	Ferroptosis	Co^2+^‐catalyzed Fenton‐like ·OH production and Russell mechanism‐enabled ^1^O_2_ emergence, while Mo^5+^ to Mo^4+^ conversion causes GSH consumption to initiate ferroptosis	[395]
LDNPs@Fe/Mn‐ZIF‐8	·O_2_^−^+·OH	Fe^2+^, Mn^2+^ (CDT), LDNPs@Fe/Mn‐ZIF‐8 (PDT)	Fe^3+^/Mn^3+^/Mn^4+^	–	The dual doping of Fe^2+^/Mn^2+^ not only reduces the band gap of ZIF‐8 photosensitizer to generate ·O_2_^−^, but also generates ·OH through CDT; and the oxidized high‐valent Fe/Mn further depletes GSH	[396]
SnO_2−x_@AGP	ABTS∙^+^	SnO_2−x_ (PDT)	ABTS∙^+^	PTT	Glucose consumption by GOx leads to an increase in H_2_O_2_, the generated ABTS∙^+^ consumes intracellular GSH and generates ROS. The oxidative stress combined with the PTT effect of SnO_2‐x_ achieves tumor ablation	[397]
Gox‐S4MSN@L‐arg	NO	L‐Arg	Tetrasulfide	Ferroptosis; Starvation therapy	The doped tetrasulfide bond reacts with GSH to induce ferroptosis, and the GOx consumes glucose to generate H_2_O_2_. The exposed L‐Arg generates NO under H_2_O_2_, achieving ferroptosis/starvation/NO therapy	[398]

Abbreviation: PUFA, Polyunsaturated fatty acids; GOx, Glucose oxidase; GSHOx: Glutathione oxidase.

Besides GSH, increased expression of antioxidant enzymes in cells like catalase also decreases ROS production,^[^ ^362^, ^373^ ^]^ limiting the effect of ROS/RNS‐based cancer therapies.^[^ ^374^ ^]^ Previous investigations have revealed that exogenous H_2_S can inhibit catalase activity in cancer cells.^[^ ^375^ ^]^ Inspired by this biochemical event, several nanosystems that integrate the H_2_S donor with the ROS generator can generate H_2_S gas and ROS in response to the acidic TME,^[^ ^376^ ^]^ achieving a “1+1>2” synergistic anti‐tumor treatment (**Table** **5**). Besides, He et al. developed Z‐scheme SnS_1.68_‐WO_2.41_ nanocatalyst to achieve NIR‐photocatalytic generation of oxidative holes, which oxidizes/consumes intratumoral over‐expressed GSH and simultaneously generate hydrogen molecules. The generated hydrogen molecules and GSH consumption inhibited cellular energy metabolism and destroyed the cellular anti‐oxidation defense system by GSH decrease and subsequent ROS increase, realizing combined hole/hydrogen therapy.^[^ ^377^ ^]^ Similarly, Du's group also designed an asymmetric and lollipop‐like nanostructure consisting of gold nanorod/titanium dioxide to produce ROS and H_2_ in TME for enhanced PTT/PDT against hypoxic tumors.^[^ ^378^ ^]^

**Table 5:** Summary of the representative nanomaterials that simultaneously generate ROS and generate H_2_S*/H_2_.

Materials	Reduction substance	H_2_S/H_2_ generating material	Free radicals/RNS type	Free radicals/RNS generating element/material	Other treatment type	Comment	Ref
FeS@BSA	H_2_S	FeS	·OH	Fe^2+^ (CDT)	–	FeS releases H_2_S and Fe^2+^ under acidic TME, H_2_S inhibits catalase activity and Fe^2+^ catalyzes the generation of ·OH to amplify ROS	[376a]
HPCu_7_S_4_@MCF‐7	H_2_S	HPCu_7_S_4_	·OH	Cu^+^ (CDT)	PTT	Cu and H_2_S generated by HPCu_7_S_4_ under acidic TME initiate CDT and gas therapy, respectively	[376b]
CuFe_2_O_4_	H_2_S^a)^	CuFe_2_O_4_ ^a)^	·OH	Cu^+^, Fe^2+^	PTT	When the high concentration of H_2_S in colon cancer is depleted by Cu^2+^ and Fe^3+^, the oxidized Cu^+^ and Fe^2+^ drive CDT and PTT for synergistic therapy	[399]
CaO_2_‐HA NPs	H_2_S	tetra‐sulfide bonds in DMOS	^1^O_2_	Chloroperoxidase	–	Under acidic TEM, the four‐sulfide bond‐breaking process consumes GSH, and at the same time, the released CPO generates ^1^O_2_, while CaO_2_ releases Ca^2+^ and H_2_O_2_. ROS accumulated by these strategies to oxidative stress	[376d]
TiS*_X_* NSs	H_2_S	TiS*_X_* NSs	^1^O_2_	TiS*_X_* NSs (SDT)	Immunotherapy	TiS_X_ NSs generate H_2_S under acidic TEM, followed by the generation of S defects and surface oxidized TiO_X_ to generate ^1^O_2_ under US. The produced H_2_S and ^1^O_2_ inhibit mitochondrial respiration and ATP synthesis, leading to cell apoptosis	[376e]
ZNNPs@FA	H_2_S^a)^	ZNNPs@FA^a)^	^1^O_2_	ZNNPs	–	ZM1068‐NB consumes H_2_S while producing NIR and PA signal, which not only cause mitochondrial damage, but the decomposition product of ZM1068‐ketone can generate ^1^O_2_ by PDT	[400]
TSH	H_2_S	(NH_4_)_2_S	·OH+^1^O_2_	TDCAc (PDT)	–	(NH_4_)_2_S release H_2_S to inhibit catalase activity and induce iron pools in cells to accumulate ·OH. TDCAc generates ^1^O_2_ and heat through Type‐I PDT and PTT	[376c]
Z‐scheme SnS_1.68_‐WO_2.41_	H_2_	SnS_1.68_‐WO_2.41_	·OH	SnS_1.68_‐WO_2.41_	GSH depletion	NIR excitation of WO_3‐x_ injects hot electrons into SnS_2‐y_ to achieve controlled hydrogen evolution and hole‐synchronized GSH oxidation, thereby disrupting cellular ADS to synergistically induce tumor apoptosis	[377]
PCN‐224@Pd/H_2_	H_2_	Pd	^1^O_2_	Porphyrin (PDT)	–	H_2_ released from Pd nanocrystals and ^1^O_2_ generated by porphyrin on NPMOF under PDT do not interfere with each other and synergistically induce cell death	[401]
L‐TiO_2_‐GNR	H_2_	TiO_2_‐GNR	·OH+·O_2_^−^+^1^O_2_	TiO_2_‐GNR (PDT)	PTT	The electrons generated by GNRs under NIR were injected into the conduction band of L‐TiO_2_ through the Schottky junction. Both of these electrons and the remaining holes on GNRs generate ·OH, ·O_2_^−^, ^1^O_2,_ and H_2_	[378]

^a)^ For colon cancer treatment, nanomaterials exert therapeutic effect by depleting H_2_S.

Abbreviation: ADS, Anti‐oxidation defense system; GNRs, Gold nanorods; NPMOF: Nano‐porphyrin metal–organic frameworks.

Above in vitro and in vivo results manifested that the integration of ROS/RNS production with the inhibition of antioxidant mechanisms could overcome the limitation of the ROS/RNS‐dependent strategies and holds great promise to acquire the synergistic enhanced therapeutic effect. Future studies are needed to understand the antioxidant mechanisms of different types of cancer cells and design intelligent nanosystems that can precisely control the generation of ROS/RNS‐based strategy and the suppression of specific antioxidant mechanisms.

## 4 Conclusion and Future Prospective

In general, intracellular ROS levels are higher in cancer cells than in normal counterparts. Cancer cells protect themselves from oxidative stress through the upregulation of multiple antioxidant pathways to buffer ROS levels for survival and growth, causing greater tumor survival and chemo/radioresistance. Disrupting the redox balance in cancer through overloading cancer cells or its environment with excessive oxidative stress or modulating the reductive stress underlying the regulation of antioxidant enzymes and metabolic pathways in the TME will provide novel anticancer therapeutic approaches. By taking advantage of responsive nanomaterials, a series of responsive nanomaterials have been designed to target the redox imbalance in cancer cells for selectively destroying cancer cells, triggering mechanisms such as apoptosis, autophagy, necrosis, or ferroptosis. These agents could either activate a direct increase in ROS production and/or induce an indirect elevation of ROS by inhibiting or decreasing the antioxidative processes of ROS removal above the safe threshold or cause the accumulation of superfluous reducing equivalents leading to the death of cancer cells. Therefore, in this review, we introduced stimuli‐responsive nanomaterials that selectively tip the redox imbalance in cancer cells over the threshold for activating cell death in response to external stimuli and tumor internal stimuli. While significant progress has been made in nanomaterials targeting redox imbalance, ongoing research is needed to address the remaining limitations and difficulties for their development into effective anti‐tumor treatments.

First, a large number of responsive nanomaterials are being studied for their ability to regulate the redox balance in cancer cells for the management of cancer. It remains to be seen which of these will prove to be the most clinically effective agents. To develop them to become an actual anti‐tumor treatment, it is important to more thoroughly understand the transition of the metabolic profile under nanomaterials‐induced oxidative and reductive damages in the TME. A recent study^[^ ^50b^ ^]^ employed mRNA analysis to identify differential gene expression profiles associated with cell apoptosis and ferroptosis in various breast cancer cell lines. Metere et al. also have reported that oxidative stress provokes the alteration of the metabolic profile in thyroid cancer cells. Through metabolomic analysis, this study revealed increases in lactate and aromatic amino acids, such as tyrosine and phenylalanine, and an average decrease in citric acid in thyroid cancer tissue compared to healthy tissue.^[^ ^380^ ^]^ Therefore, the application of genomic/transcriptomic/proteomic analysis and metabolomic analysis might help understand the antitumor mechanism of redox‐regulating nanomaterials.

Second, most tumor cells exhibit abnormally high expression of ROS due to factors such as hypoxia.^[^ ^381^ ^]^ However, certain tumor cells such as human breast cancer cells and cancer stem cells were proposed not to maintain high ROS levels as they have increased antioxidant capacity.^[^ ^382^ ^]^ This enables them to acquire tolerance/resistance to chemotherapy and radiotherapy. Therefore, it is crucial to understand the redox states of tumor cells as well as the oxidative and reductive stress underlying the regulation of antioxidant enzymes and metabolic pathways in different types of tumors, which will provide a guide on designing nanomaterials specific to different tumors.

Third, the redox homeostasis of tumors is not a static balance, and it is constantly subjected to dynamic biochemical changes and influenced by the heterogeneity of the TME, the stroma, endothelium, immune cells, and circulating cancer cells.^[^ ^26^ ^]^ For example, a large number of evidence indicates progressively increased ROS production with advanced stages of malignancy. Early‐onset primary tumors will more closely resemble normal cells with greater antioxidative defense systems and be less likely to succumb to the actions of excessive ROS production. Responsive nanomaterials that can activate a direct increase in ROS production and/or indirect elevation of ROS will have a greater impact on preventing metastasis and addressing more advanced stages of cancer. After undergoing metabolic reprogramming to higher antioxidative defense mechanisms, cancer cells might be more susceptible to the cytotoxic actions of ROS‐based therapy. Moreover, ROS‐based responsive nanomaterials might be used in combination with commonly used chemotherapeutic agents and other forms of cancer treatment, such as radiotherapy. Therefore, it is important to monitor the transition of redox homeostasis and optimize the timing of administration for the clinical application of responsive nanomaterials. Incorporating redox state‐imaging agents into redox‐regulating nanomaterials might allow for spatial and temporal monitoring of the transition of the redox homeostasis of tumors during the treatment process, which will help to understand the subcellular localization of nanomaterials and their pathway or mechanism to exert redox regulation.

Finally, although enormous progress has been made in using nanomaterials for inducing excessive reactive oxidative species generation to induce tumor destruction, only a few nanomedicines have been approved in clinics or clinical trials. Fortunately, several nanomaterials exhibit vast clinical implications and exciting therapeutic potential.^[^ ^379^ ^]^ However, no actively targeted or stimulus‐responsive nanomedicine has yet been granted regulatory approval. The complexity of the nano‐bio interface in the dynamic, highly heterogeneous pathological characteristics of malignant tumors, poor controllability and low specificity of nanosystems, and difficulty of large‐scale and repeated preparation are still insurmountable challenges for the transition of responsive nanomaterials from bench to bedside. The application of artificial intelligence and machine learning may improve the simulation and modeling process of in vitro and in vivo pharmacokinetics and pharmacodynamics, absorption, distribution, metabolism, and excretion of nanomaterials, enhancing the understanding of their specific toxicity mechanisms, thus accelerating the development of safe and effective nanomedicine.

## Conflict of Interest

The authors declare no conflict of interest.

## References

[1] H. Sung , J. Ferlay , R. L. Siegel , M. Laversanne , I. Soerjomataram , A. Jemal , F. Bray , Ca‐Cancer J. Clin. 2021, 71, 209. 33538338 10.3322/caac.21660

[2] a) D. Hanahan , R. A. Weinberg , Cell 2011, 144, 646; 21376230 10.1016/j.cell.2011.02.013

[3] T. Fiaschi , P. Chiarugi , Int. J. Cell Biol. 2012, 2012, 762825. 22666258 10.1155/2012/762825 PMC3361160

[4] a) Y. Mitsuishi , K. Taguchi , Y. Kawatani , T. Shibata , T. Nukiwa , H. Aburatani , M. Yamamoto , H. Motohashi , Cancer Cell 2012, 22, 66; 22789539 10.1016/j.ccr.2012.05.016

[5] L. B. Sullivan , N. S. Chandel , Cancer Metab. 2014, 2, 1. 25671107 10.1186/2049-3002-2-17 PMC4323058

[6] a) J. Carew , Y. Zhou , M. Albitar , J. Carew , M. Keating , P. Huang , Leukemia 2003, 17, 1437; 12886229 10.1038/sj.leu.2403043

[7] V. Raimondi , F. Ciccarese , V. Ciminale , Br. J. Cancer 2020, 122, 168. 31819197 10.1038/s41416-019-0651-y PMC7052168

[8] R. A. Cairns , I. S. Harris , T. W. Mak , Nat. Rev. Cancer 2011, 11, 85. 21258394 10.1038/nrc2981

[9] I. S. Harris , A. E. Treloar , S. Inoue , M. Sasaki , C. Gorrini , K. C. Lee , K. Y. Yung , D. Brenner , C. B. Knobbe‐Thomsen , M. A. Cox , Cancer Cell 2015, 27, 211. 25620030 10.1016/j.ccell.2014.11.019

[10] C. W. Barrett , W. Ning , X. Chen , J. J. Smith , M. K. Washington , K. E. Hill , L. A. Coburn , R. M. Peek , R. Chaturvedi , K. T. Wilson , Cancer Res. 2013, 73, 1245. 23221387 10.1158/0008-5472.CAN-12-3150 PMC3563732

[11] C. A. Davison , S. M. Durbin , M. R. Thau , V. R. Zellmer , S. E. Chapman , J. Diener , C. Wathen , W. M. Leevy , Z. T. Schafer , Cancer Res. 2013, 73, 3704. 23771908 10.1158/0008-5472.CAN-12-2482

[12] a) M. L. Gomez , N. Shah , T. C. Kenny , E. C. Jenkins Jr, D. Germain , Oncogene 2019, 38, 5751; 31222103 10.1038/s41388-019-0839-x PMC6639133

[13] a) G. M. DeNicola , F. A. Karreth , T. J. Humpton , A. Gopinathan , C. Wei , K. Frese , D. Mangal , K. H. Yu , C. J. Yeo , E. S. Calhoun , Nature 2011, 475, 106; 21734707 10.1038/nature10189 PMC3404470

[14] C. Wiel , K. Le Gal , M. X. Ibrahim , C. A. Jahangir , M. Kashif , H. Yao , D. V. Ziegler , X. Xu , T. Ghosh , T. Mondal , Cell 2019, 178, 330. 31257027 10.1016/j.cell.2019.06.005

[15] a) X. Dai , D. Wang , J. Zhang , Apoptosis 2021, 26, 385; 34236569 10.1007/s10495-021-01682-0

[16] a) M. Ebbing , K. H. Bønaa , O. Nygård , E. Arnesen , P. M. Ueland , J. E. Nordrehaug , K. Rasmussen , I. Njølstad , H. Refsum , D. W. Nilsen , JAMA, J. Am. Med. Assoc. 2009, 302, 2119; 10.1001/jama.2009.1622 19920236

[17] T. Zaidieh , J. R. Smith , K. E. Ball , Q. An , BMC Cancer 2019, 19, 1. 31842863 10.1186/s12885-019-6438-y PMC6916036

[18] a) C. Martin‐Cordero , A. Jose Leon‐Gonzalez , J. Manuel Calderon‐Montano , E. Burgos‐Moron , M. Lopez‐Lazaro , Curr. Drug Targets 2012, 13, 1006; 22594470 10.2174/138945012802009044

[19] Y. Wu , Y. Li , G. Lv , W. Bu , Chem. Sci. 2022, 13, 2202. 35310479 10.1039/d1sc06315d PMC8864817

[20] a) F. Ciccarese , V. Raimondi , E. Sharova , M. Silic‐Benussi , V. Ciminale , Antioxidants 2020, 9, 211; 32143322 10.3390/antiox9030211 PMC7139659

[21] H. Sies , C. Berndt , D. P. Jones , Ann. Rev. Biochem. 2017, 86, 715. 28441057 10.1146/annurev-biochem-061516-045037

[22] B. Yang , Y. Chen , J. Shi , Chem. Rev. 2019, 119, 4881. 30973011 10.1021/acs.chemrev.8b00626

[23] N. Kwon , D. Kim , K. Swamy , J. Yoon , Coord. Chem. Rev. 2021, 427, 213581.

[24] a) S. M. Clemente , O. H. Martínez‐Costa , M. Monsalve , A. K. Samhan‐Arias , Molecules 2020, 25, 5144; 33167334 10.3390/molecules25215144 PMC7663840

[25] B. I. Ratnikov , D. A. Scott , A. L. Osterman , J. W. Smith , Z. e. A. Ronai , Oncogene 2017, 36, 147. 27270434 10.1038/onc.2016.198 PMC5140782

[26] J. Kim , J. Kim , J.‐S. Bae , Exp. Mol. Med. 2016, 48, e269. 27811934 10.1038/emm.2016.119 PMC5133371

[27] S. S. Sabharwal , P. T. Schumacker , Nat. Rev. Cancer 2014, 14, 709. 25342630 10.1038/nrc3803 PMC4657553

[28] V. Sosa , T. Moliné , R. Somoza , R. Paciucci , H. Kondoh , M. E. LLeonart , Ageing Res. Rev. 2013, 12, 376. 23123177 10.1016/j.arr.2012.10.004

[29] R. O. Poyton , K. A. Ball , P. R. Castello , Trends Endocrinol. Metab. 2009, 20, 332. 19733481 10.1016/j.tem.2009.04.001

[30] T. P. Szatrowski , C. F. Nathan , Cancer Res. 1991, 51, 794. 1846317

[31] J. Kim , H. R. Cho , H. Jeon , D. Kim , C. Song , N. Lee , S. H. Choi , T. Hyeon , J. Am. Chem. Soc. 2017, 139, 10992. 28737393 10.1021/jacs.7b05559

[32] J. D. Hayes , A. T. Dinkova‐Kostova , K. D. Tew , Cancer Cell 2020, 38, 167. 32649885 10.1016/j.ccell.2020.06.001 PMC7439808

[33] Y.‐T. Jiao , Y.‐R. Kang , M.‐Y. Wen , H.‐Q. Wu , X. Zhang , W.‐H. Huang , Angew. Chem., Int. Ed. 2023, 62, e202313612. 10.1002/anie.202313612 37909054

[34] D. Trachootham , J. Alexandre , P. Huang , Nat. Rev. Drug Discovery 2009, 8, 579. 19478820 10.1038/nrd2803

[35] M. Najafi , N. Hashemi Goradel , B. Farhood , E. Salehi , M. S. Nashtaei , N. Khanlarkhani , Z. Khezri , J. Majidpoor , M. Abouzaripour , M. Habibi , J. Cell. Biochem. 2019, 120, 2756. 30270458 10.1002/jcb.27646

[36] B. Marengo , M. Nitti , A. L. Furfaro , R. Colla , C. D. Ciucis , U. M. Marinari , M. A. Pronzato , N. Traverso , C. Domenicotti , Oxid. Med. Cell. Longev. 2016, 2016, 6235641. 27418953 10.1155/2016/6235641 PMC4932173

[37] S. Kumar , M. Lemos , M. Sharma , V. Shriram , Adv. Appl. Sci. Res. 2011, 2, 129.

[38] M. K. Foret , R. Lincoln , S. Do Carmo , A. C. Cuello , G. Cosa , Chem. Rev. 2020, 120, 12757. 33211489 10.1021/acs.chemrev.0c00761

[39] J. Zhang , X. Wang , V. Vikash , Q. Ye , D. Wu , Y. Liu , W. Dong , Oxid. Med. Cell. Longev. 2016, 2016, 4350965. 26998193 10.1155/2016/4350965 PMC4779832

[40] J. Wang , K. Pantopoulos , Biochem. J. 2011, 434, 365. 21348856 10.1042/BJ20101825 PMC3048577

[41] Z. Zou , H. Chang , H. Li , S. Wang , Apoptosis 2017, 22, 1321. 28936716 10.1007/s10495-017-1424-9

[42] D. S. Kalinowski , C. Stefani , S. Toyokuni , T. Ganz , G. J. Anderson , N. V. Subramaniam , D. Trinder , J. K. Olynyk , A. Chua , P. J. Jansson , Biochim. Biophys. Acta, Mol. Cell Res. 2016, 1863, 727. 10.1016/j.bbamcr.2016.01.026 26844773

[43] C. Zhang , W. Bu , D. Ni , S. Zhang , Q. Li , Z. Yao , J. Zhang , H. Yao , Z. Wang , J. Shi , Angew. Chem., Int. Ed. 2016, 128, 2141. 10.1002/anie.201510031 26836344

[44] a) Y. Wu , T. Guo , Y. Qiu , Y. Lin , Y. Yao , W. Lian , L. Lin , J. Song , H. Yang , Chem. Sci. 2019, 10, 7068; 31588274 10.1039/c9sc01070j PMC6676468

[45] a) T. Xu , Y. Ma , Q. Yuan , H. Hu , X. Hu , Z. Qian , J. K. Rolle , Y. Gu , S. Li , ACS Nano 2020, 14, 3414; 32155051 10.1021/acsnano.9b09426

[46] a) W. Zuo , W. Chen , J. Liu , S. Huang , L. Chen , Q. Liu , N. Liu , Q. Jin , Y. Li , P. Wang , ACS Appl. Mater. Interfaces 2022, 14, 5053; 35040616 10.1021/acsami.1c22432

[47] a) J. Wang , H. Ding , Y. Zhu , Y. Liu , M. Yu , H. Cai , R. Ao , H. Huang , P. Gong , Y. Liao , Angew. Chem., Int. Ed. 2023, 135, e202302255; 10.1002/anie.202302255 36959091

[48] X. Li , Q. Zhou , A. A.‐W. M. M. Japir , D. Dutta , N. Lu , Z. Ge , ACS Nano 2022, 16, 14982. 36017992 10.1021/acsnano.2c06026

[49] L. Feng , R. Xie , C. Wang , S. Gai , F. He , D. Yang , P. Yang , J. Lin , ACS Nano 2018, 12, 11000. 30339353 10.1021/acsnano.8b05042

[50] a) J. S. Koo , S. Y. Lee , M. O. K. Azad , M. Kim , S. J. Hwang , S. Nam , S. Kim , B.‐J. Chae , W.‐S. Kang , H.‐J. Cho , Int. J. Pharm. 2019, 558, 388; 30665001 10.1016/j.ijpharm.2019.01.018

[51] C. Liu , W. Chen , Z. Qing , J. Zheng , Y. Xiao , S. Yang , L. Wang , Y. Li , R. Yang , Anal. Chem. 2016, 88, 3998. 26948406 10.1021/acs.analchem.6b00267

[52] a) B. Yang , J. Shi , J. Am. Chem. Soc. 2020, 142, 21775; 33314928 10.1021/jacs.0c09984

[53] J. Yang , H. Yao , Y. Guo , B. Yang , J. Shi , Angew. Chem., Int. Ed. 2022, 134, e202200480. 10.1002/anie.202200480 35143118

[54] a) F. Liu , L. Lin , Y. Zhang , Y. Wang , S. Sheng , C. Xu , H. Tian , X. Chen , Adv. Mater. 2019, 31, 1902885; 10.1002/adma.201902885 31423690

[55] a) L. An , C. Wang , Q. Tian , C. Tao , F. Xue , S. Yang , X. Zhou , X. Chen , G. Huang , Nano Today 2022, 43, 101397;

[56] H. Zhu , S. Huang , M. Ding , Z. Li , J. Li , S. Wang , D. T. Leong , ACS Appl. Mater. Interfaces 2022, 14, 25183. 35638599 10.1021/acsami.2c05170

[57] Y. Qian , J. Zhang , J. Zou , X. Wang , X. Meng , H. Liu , Y. Lin , Q. Chen , L. Sun , W. Lin , Theranostics 2022, 12, 3690. 35664059 10.7150/thno.70841 PMC9131281

[58] a) T. He , X. Qin , C. Jiang , D. Jiang , S. Lei , J. Lin , W.‐G. Zhu , J. Qu , P. Huang , Theranostics 2020, 10, 2453; 32194812 10.7150/thno.42981 PMC7052883

[59] G. Liu , J. Zhu , H. Guo , A. Sun , P. Chen , L. Xi , W. Huang , X. Song , X. Dong , Angew. Chem., Int. Ed. 2019, 58, 18641. 10.1002/anie.201910815 31605417

[60] K. Xu , Y. Cheng , J. Yan , Y. Feng , R. Zheng , X. Wu , Y. Wang , P. Song , H. Zhang , Nano Res. 2019, 12, 2947.

[61] W.‐L. Wang , Z. Guo , Y. Lu , X.‐C. Shen , T. Chen , R.‐T. Huang , B. Zhou , C. Wen , H. Liang , B.‐P. Jiang , ACS Appl. Mater. Interfaces 2019, 11, 17294. 30977628 10.1021/acsami.9b04531

[62] P. Liu , Y. Wang , L. An , Q. Tian , J. Lin , S. Yang , ACS Appl. Mater. Interfaces 2018, 10, 38833. 30351904 10.1021/acsami.8b15678

[63] X. Wang , X. Zhong , L. Bai , J. Xu , F. Gong , Z. Dong , Z. Yang , Z. Zeng , Z. Liu , L. Cheng , J. Am. Chem. Soc. 2020, 142, 6527. 32191455 10.1021/jacs.9b10228

[64] L.‐S. Lin , T. Huang , J. Song , X.‐Y. Ou , Z. Wang , H. Deng , R. Tian , Y. Liu , J.‐F. Wang , Y. Liu , J. Am. Chem. Soc. 2019, 141, 9937. 31199131 10.1021/jacs.9b03457

[65] a) Q. Tian , F. Xue , Y. Wang , Y. Cheng , L. An , S. Yang , X. Chen , G. Huang , Nano Today 2021, 39, 101162;

[66] X. Zhang , Y. Lin , R. J. Gillies , J. Nucl. Med. 2010, 51, 1167. 20660380 10.2967/jnumed.109.068981 PMC4351768

[67] a) X. Chen , H. Zhang , M. Zhang , P. Zhao , R. Song , T. Gong , Y. Liu , X. He , K. Zhao , W. Bu , Adv. Funct. Mater. 2020, 30, 1908365;

[68] X. Zhang , C. He , Y. Chen , C. Chen , R. Yan , T. Fan , Y. Gai , T. Yang , Y. Lu , G. Xiang , Biomaterials 2021, 275, 120987. 34175561 10.1016/j.biomaterials.2021.120987

[69] X. Song , J. Xu , C. Liang , Y. Chao , Q. Jin , C. Wang , M. Chen , Z. Liu , Nano Lett. 2018, 18, 6360. 30247918 10.1021/acs.nanolett.8b02720

[70] W. Feng , X. Han , R. Wang , X. Gao , P. Hu , W. Yue , Y. Chen , J. Shi , Adv. Mater. 2019, 31, 1805919. 10.1002/adma.201805919 30536723

[71] Y. Zhang , Y. Wan , Y. Liao , Y. Hu , T. Jiang , T. He , W. Bi , J. Lin , P. Gong , L. Tang , Sci. Bull. 2020, 65, 564. 10.1016/j.scib.2019.12.024 36659188

[72] C. Fang , Z. Deng , G. Cao , Q. Chu , Y. Wu , X. Li , X. Peng , G. Han , Adv. Funct. Mater. 2020, 30, 1910085.

[73] P. R. Ogilby , Chem. Soc. Rev. 2010, 39, 3181. 20571680 10.1039/b926014p

[74] A. Shrivastava , L. M. Aggarwal , S. P. Mishra , H. D. Khanna , U. P. Shahi , S. Pradhan , Indian J. Biochem. Biophys. 2019, 56, 7.

[75] M. Riethmüller , N. Burger , G. Bauer , Redox Biol. 2015, 6, 157. 26225731 10.1016/j.redox.2015.07.006 PMC4532730

[76] T. Chen , P. Hou , Y. Zhang , R. Ao , L. Su , Y. Jiang , Y. Zhang , H. Cai , J. Wang , Q. Chen , Angew. Chem., Int. Ed. 2021, 133, 15133. 10.1002/anie.202102097 33871140

[77] C. Lu , C. Zhang , P. Wang , Y. Zhao , Y. Yang , Y. Wang , H. Yuan , S. Qu , X. Zhang , G. Song , Chem 2020, 6, 2314.

[78] Y. Chen , J. Deng , F. Liu , P. Dai , Y. An , Z. Wang , Y. Zhao , Adv. Healthcare Mater. 2019, 8, 1900366. 10.1002/adhm.201900366 31365192

[79] K. Schröder , Redox Biol. 2020, 34, 101512. 32480354 10.1016/j.redox.2020.101512 PMC7262010

[80] M. Li , J. Xia , R. Tian , J. Wang , J. Fan , J. Du , S. Long , X. Song , J. W. Foley , X. Peng , J. Am. Chem. Soc. 2018, 140, 14851. 30362735 10.1021/jacs.8b08658

[81] a) G.‐Y. Liou , P. Storz , Free Radic. Res. 2010, 44, 479; 20370557 10.3109/10715761003667554 PMC3880197

[82] a) M. Li , Y. Shao , J. H. Kim , Z. Pu , X. Zhao , H. Huang , T. Xiong , Y. Kang , G. Li , K. Shao , J. Am. Chem. Soc. 2020, 142, 5380; 32105455 10.1021/jacs.0c00734

[83] B. Yang , Y. Chen , J. Shi , Angew. Chem., Int. Ed. 2020, 132, 9780.

[84] Y. Dai , Z. Yang , S. Cheng , Z. Wang , R. Zhang , G. Zhu , Z. Wang , B. C. Yung , R. Tian , O. Jacobson , Adv. Mater. 2018, 30, 1704877. 10.1002/adma.201704877 29315862

[85] S. Cai , J. Liu , J. Ding , Z. Fu , H. Li , Y. Xiong , Z. Lian , R. Yang , C. Chen , Angew. Chem., Int. Ed. 2022, 61, e202204502. 10.1002/anie.202204502 35972794

[86] Z. Ma , Y. Yang , Y. Jiang , B. Xi , T. Yang , X. Peng , X. Lian , K. Yan , H. Liu , Chem. Eng. J. 2017, 311, 183.

[87] S. Ding , M. Li , H. Gong , Q. Zhu , G. Shi , A. Zhu , Anal. Chem. 2020, 92, 2543. 31927939 10.1021/acs.analchem.9b04139

[88] C. Y. Toe , C. Tsounis , J. Zhang , H. Masood , D. Gunawan , J. Scott , R. Amal , Energy Environ. Sci. 2021, 14, 1140.

[89] S. Xiong , S. George , Z. Ji , S. Lin , H. Yu , R. Damoiseaux , B. France , K. W. Ng , S. C. J. Loo , Arch. Toxicol. 2013, 87, 99. 22885792 10.1007/s00204-012-0912-5 PMC5889301

[90] A. Glasauer , N. S. Chandel , Biochem. Pharmacol. 2014, 92, 90. 25078786 10.1016/j.bcp.2014.07.017

[91] S. Zhu , X. Li , J. Kang , X. Duan , S. Wang , Environ. Sci. Technol. 2018, 53, 307. 30479119 10.1021/acs.est.8b04669

[92] R. Mittler , Trends Plant Sci. 2017, 22, 11. 27666517 10.1016/j.tplants.2016.08.002

[93] L. Wang , K. Xiao , H. Zhao , Water Res. 2023, 235, 119925. 37028213 10.1016/j.watres.2023.119925

[94] P. Yang , Y. Long , W. Huang , D. Liu , Appl. Catal., B 2023, 324, 122245.

[95] A. Blazquez‐Castro , Redox Biol. 2017, 13, 39. 28570948 10.1016/j.redox.2017.05.011 PMC5451181

[96] P. Rao , E. Hayon , Biochem. Biophys. Res. Commun. 1973, 51, 468. 4693487 10.1016/0006-291x(73)91280-1

[97] Y. Sheng , I. A. Abreu , D. E. Cabelli , M. J. Maroney , A.‐F. Miller , M. Teixeira , J. S. Valentine , Chem. Rev. 2014, 114, 3854. 24684599 10.1021/cr4005296 PMC4317059

[98] M. Hayyan , M. A. Hashim , I. M. AlNashef , Chem. Rev. 2016, 116, 3029. 26875845 10.1021/acs.chemrev.5b00407

[99] J. Fujii , T. Homma , T. Osaki , Antioxidants 2022, 11, 501. 35326151 10.3390/antiox11030501 PMC8944419

[100] J. C. Toledo Jr, O. Augusto , Chem. Res. Toxicol. 2012, 25, 975. 22449080 10.1021/tx300042g

[101] a) J. S. Stamler , D. J. Singel , J. Loscalzo , Science 1992, 258, 1898; 1281928 10.1126/science.1281928

[102] S. Habib , A. Ali , Indian J. Clin. Biochem. 2011, 26, 3. 22211007 10.1007/s12291-011-0108-4 PMC3068772

[103] T. Kim , Y. Nah , J. Kim , S. Lee , W. J. Kim , Acc. Chem. Res. 2022, 55, 2384. 35786846 10.1021/acs.accounts.2c00159

[104] W. Zhou , Y. Cao , D. Sui , C. Lu , Anal. Chem. 2016, 88, 2659. 26894599 10.1021/acs.analchem.5b03827

[105] a) R. Radi , G. Peluffo , M. A. N. Alvarez , M. Naviliat , A. Cayota , Free Radical Biol. Med. 2001, 30, 463; 11182518 10.1016/s0891-5849(00)00373-7

[106] J. S. Beckman , W. H. Koppenol , Am. J. Physiol. Cell Physiol. 1996, 271, C1424. 10.1152/ajpcell.1996.271.5.C1424 8944624

[107] R. Radi , Proc. Natl. Acad. Sci. USA 2018, 115, 5839. 29802228 10.1073/pnas.1804932115 PMC6003358

[108] a) P. Neta , R. E. Huie , A. B. Ross , J. Phys. Chem. Ref. Data 1988, 17, 1027;

[109] M. Kelm , Biochim. Biophys. Acta, Bioenerg. 1999, 1411, 273. 10.1016/s0005-2728(99)00020-1 10320663

[110] W. A. Pryor , J. W. Lightsey , Science 1981, 214, 435. 17730242 10.1126/science.214.4519.435

[111] D. A. Wink , J. F. Darbyshire , R. W. Nims , J. E. Saavedra , P. C. Ford , Chem. Res. Toxicol. 1993, 6, 23. 8448345 10.1021/tx00031a003

[112] J. Sun , X. Zhang , X. Wang , J. Peng , G. Song , Y. Di , F. Feng , S. Wang , Angew. Chem., Int. Ed. 2022, 61, e202213765. 10.1002/anie.202213765 36342403

[113] a) S.‐Y. Oh , H.‐W. Kim , J.‐M. Park , H.‐S. Park , C. Yoon , J. Hazard. Mater. 2009, 168, 346; 19285795 10.1016/j.jhazmat.2009.02.065

[114] V. Hasija , P. Raizada , V. K. Thakur , T. Ahamad , S. M. Alshehri , S. Thakur , V.‐H. Nguyen , Q. Van Le , P. Singh , Curr. Opin. Chem. Eng. 2022, 37, 100841.

[115] X. Li , X. Huang , S. Xi , S. Miao , J. Ding , W. Cai , S. Liu , X. Yang , H. Yang , J. Gao , J. Am. Chem. Soc. 2018, 140, 12469. 30165734 10.1021/jacs.8b05992

[116] C. Liu , B. Wu , Chem. Eng. J. 2018, 335, 865.

[117] S. Giannakis , K.‐Y. A. Lin , F. Ghanbari , Chem. Eng. J. 2021, 406, 127083.

[118] R. Hu , X. Chen , Z. Li , G. Zhao , L. Ding , L. Chen , C. Dai , Y. Chen , B. Zhang , Adv. Mater. 2023, 35, 2306469. 10.1002/adma.202306469 37669827

[119] a) Y. Ji , J. Bai , J. Li , T. Luo , L. Qiao , Q. Zeng , B. Zhou , Water Res. 2017, 125, 512; 28957768 10.1016/j.watres.2017.08.053

[120] J. E. Grebel , J. J. Pignatello , W. A. Mitch , Environ. Sci. Technol. 2010, 44, 6822. 20681567 10.1021/es1010225

[121] F. Z. Meghlaoui , S. Merouani , O. Hamdaoui , M. Bouhelassa , M. Ashokkumar , Sep. Purif. Technol. 2019, 227, 115685.

[122] Z. Luo , Z. Yi , X. Liu , Acc. Chem. Res. 2023, 56, 425. 36745051 10.1021/acs.accounts.2c00681

[123] M. S. Koo , S. Han , K. Cho , W. Choi , ACS ES&T Eng 2021, 1, 1287.

[124] N. Schürmann , P. Forrer , O. Casse , J. Li , B. Felmy , A.‐V. Burgener , N. Ehrenfeuchter , W.‐D. Hardt , M. Recher , C. Hess , Nat. Microbiol. 2017, 2, 1. 10.1038/nmicrobiol.2016.268 28112722

[125] L. Wang , M. Bassiri , R. Najafi , K. Najafi , J. Yang , B. Khosrovi , W. Hwong , E. Barati , B. Belisle , C. Celeri , J. Burns Wounds 2007, 6, e5. 17492050 PMC1853323

[126] C. L. Hawkins , Essays Biochem. 2020, 64, 75. 31867603 10.1042/EBC20190045

[127] J. Q. Del Rosso , N. Bhatia , J. Clin. Aesthet. Dermatol. 2018, 11, 36. PMC6303114 30588272

[128] G. Bauer , J. Inorg. Biochem. 2018, 179, 10. 29156213 10.1016/j.jinorgbio.2017.11.005

[129] L. K. Folkes , M. Trujillo , S. Bartesaghi , R. Radi , P. Wardman , Arch. Biochem. Biophys. 2011, 506, 242. 21147061 10.1016/j.abb.2010.12.006

[130] Y. Song , J. Jiang , J. Ma , S.‐Y. Pang , Y.‐Z. Liu , Y. Yang , C.‐W. Luo , J.‐Q. Zhang , J. Gu , W. Qin , Environ. Sci. Technol. 2015, 49, 11764. 26378975 10.1021/acs.est.5b03358

[131] A. Campos , E. Lissi , Int. J. Chem. Kinet. 1997, 29, 219.

[132] C. Henriquez , C. Aliaga , E. Lissi , Int. J. Chem. Kinet. 2002, 34, 659.

[133] K. W. Lee , Y. Wan , X. Li , X. Cui , S. Li , C. S. Lee , Adv. Healthcare Mater. 2021, 10, 2100055.

[134] C. P. Andrieux , L. Gelis , M. Medebielle , J. Pinson , J. M. Saveant , J. Am. Chem. Soc. 1990, 112, 3509.

[135] A. Krivenko , A. Kotkin , V. Kurmaz , V. Titov , V. Lopushanskaya , V. Koshechko , Theor. Exp. Chem. 2000, 36, 325.

[136] M. Cheng , B. Zhang , W. Cui , M. L. Gross , Angew. Chem., Int. Ed. 2017, 56, 14007. 10.1002/anie.201706697 PMC5663992 28901679

[137] H. Ge , J. Du , J. Zheng , N. Xu , Q. Yao , S. Long , J. Fan , X. Peng , Chem. Eng. J. 2022, 446, 137040.

[138] M. Shee , N. P. Singh , Chem. Soc. Rev. 2022, 51, 2255. 35229836 10.1039/d1cs00494h

[139] F. Gao , B. Yu , H. Cong , Y. Shen , J. Mater. Chem. B 2022, 10, 6896. 36048171 10.1039/d2tb01326f

[140] H. M. Hoang , H. E. Johnson , J. Heo , J. Biol. Chem. 2021, 297, 100982. 34293347 10.1016/j.jbc.2021.100982 PMC8353492

[141] a) L. J. Ignarro , Angew. Chem., Int. Ed. 1999, 38, 1882; 10.1002/(SICI)1521-3773(19990712)38:13/14<1882::AID-ANIE1882>3.0.CO;2-V 34182699

[142] A. Kamm , P. Przychodzen , A. Kuban‐Jankowska , D. Jacewicz , A. M. Dabrowska , S. Nussberger , M. Wozniak , M. Gorska‐Ponikowska , Nitric Oxide 2019, 93, 102. 31541733 10.1016/j.niox.2019.09.005

[143] S. Shiva , Redox Biol. 2013, 1, 40. 23710434 10.1016/j.redox.2012.11.005 PMC3661298

[144] H. Cheng , L. Wang , M. Mollica , A. T. Re , S. Wu , L. Zuo , Cancer Lett. 2014, 353, 1. 25079686 10.1016/j.canlet.2014.07.014 PMC4150837

[145] X. Zheng , Y. Jin , X. Liu , T. Liu , W. Wang , H. Yu , Bioact. Mater. 2021, 6, 4301. 33997507 10.1016/j.bioactmat.2021.04.030 PMC8105601

[146] W. Jiang , W. Dong , M. Li , Z. Guo , Q. Wang , Y. Liu , Y. Bi , H. Zhou , Y. Wang , ACS Nano 2022, 16, 3881. 35238549 10.1021/acsnano.1c09048

[147] M. Sun , Y. Sang , Q. Deng , Z. Liu , J. Ren , X. Qu , Nano Res. 2022, 15, 5273.

[148] Y. Deng , Y. Wang , F. Jia , W. Liu , D. Zhou , Q. Jin , J. Ji , ACS Nano 2021, 15, 8663. 33929183 10.1021/acsnano.1c00698

[149] S. Huerta , Future Sci. OA 2015, 1. 10.4155/fso.15.44 PMC5137992 28031862

[150] a) D. A. Riccio , M. H. Schoenfisch , Chem. Soc. Rev. 2012, 41, 3731; 22362355 10.1039/c2cs15272j PMC3341515

[151] a) P. G. Wang , M. Xian , X. Tang , X. Wu , Z. Wen , T. Cai , A. J. Janczuk , Chem. Rev. 2002, 102, 1091; 11942788 10.1021/cr000040l

[152] a) A. C. Gorren , A. Schrammel , K. Schmidt , B. Mayer , Arch. Biochem. Biophys. 1996, 330, 219; 8660650 10.1006/abbi.1996.0247

[153] H. J. Lee , D. J. Park , G. H. Choi , D.‐N. Yang , J. S. Heo , S. C. Lee , Colloids Surf., B 2016, 146, 1. 10.1016/j.colsurfb.2016.05.039 27240199

[154] Q. Song , S. Tan , X. Zhuang , Y. Guo , Y. Zhao , T. Wu , Q. Ye , L. Si , Z. Zhang , Mol. Pharmaceutics 2014, 11, 4118. 10.1021/mp5003009 25222114

[155] R. Chandrawati , J. Y. Chang , E. Reina‐Torres , C. Jumeaux , J. M. Sherwood , W. D. Stamer , A. N. Zelikin , D. R. Overby , M. M. Stevens , Adv. Mater. 2017, 29, 1604932. 28221702 10.1002/adma.201604932 PMC5400071

[156] a) H. Y. Jia , Y. Liu , X. J. Zhang , L. Han , L. B. Du , Q. Tian , Y. C. Xu , J. Am. Chem. Soc. 2009, 131, 40; 19072650 10.1021/ja808033w

[157] Y. Deng , F. Jia , X. Chen , Q. Jin , J. Ji , Small 2020, 16, 2001747. 10.1002/smll.202001747 32378343

[158] C. Yang , S. Jeong , S. Ku , K. Lee , M. H. Park , J. Control Release 2018, 279, 157. 29673643 10.1016/j.jconrel.2018.04.025

[159] G. Walford , J. Loscalzo , J. Thromb. Haemost. 2003, 1, 2112. 14521592 10.1046/j.1538-7836.2003.00345.x

[160] C. Wang , G. Tian , X. Yu , X. Zhang , Small 2023, 19, 2207261. 10.1002/smll.202207261 36808830

[161] a) W. Fan , N. Lu , P. Huang , Y. Liu , Z. Yang , S. Wang , G. Yu , Y. Liu , J. Hu , Q. He , Angew. Chem., Int. Ed. 2017, 56, 1229; 10.1002/anie.201610682 27936311

[162] a) J. Mu , L. He , W. Fan , W. Tang , Z. Wang , C. Jiang , D. Zhang , Y. Liu , H. Deng , J. Zou , Small 2020, 16, 2004016; 10.1002/smll.202004016 32985099

[163] J. S. Beckman , T. W. Beckman , J. Chen , P. A. Marshall , B. A. Freeman , Proc. Natl. Acad. Sci. USA 1990, 87, 1620. 2154753 10.1073/pnas.87.4.1620 PMC53527

[164] a) R. Radi , G. Peluffo , M. a. N. Alvarez , M. Naviliat , A. Cayota , Free Radical Biol. Med. 2001, 30, 463; 11182518 10.1016/s0891-5849(00)00373-7

[165] J. M. Pérez de la Lastra , C. A. Juan , F. J. Plou , E. Pérez‐Lebeña , Stresses 2022, 2, 53.

[166] a) R. Sullivan , C. H. Graham , Cancer Metastasis Rev. 2007, 26, 319; 17458507 10.1007/s10555-007-9062-2

[167] a) S. W. Ryter , J. Alam , A. M. Choi , Physiol. Rev. 2006, 86, 583; 16601269 10.1152/physrev.00011.2005

[168] C. C. Romão , W. A. Blättler , J. D. Seixas , G. J. Bernardes , Chem. Soc. Rev. 2012, 41, 3571. 22349541 10.1039/c2cs15317c

[169] a) Y. Zhou , W. Yu , J. Cao , H. Gao , Biomaterials 2020, 255, 120193; 32569866 10.1016/j.biomaterials.2020.120193

[170] a) B. F. Spielvogel , M. K. Das , A. T. McPhail , K. D. Onan , I. H. Hall , J. Am. Chem. Soc. 1980, 102, 6343;

[171] Y. Lin , W. Zhong , M. Wang , Z. Chen , C. Lu , H. Yang , ACS Appl. Bio Mater. 2021, 4, 4557. 10.1021/acsabm.1c00285 35006792

[172] C. Gao , X. Liang , Z. Guo , B.‐P. Jiang , X. Liu , X.‐C. Shen , ACS Omega 2018, 3, 2683. 30023846 10.1021/acsomega.8b00052 PMC6044757

[173] a) N. S. Sitnikov , Y. Li , D. Zhang , B. Yard , H. G. Schmalz , Angew. Chem., Int. Ed. 2015, 54, 12314; 10.1002/anie.201502445 26037072

[174] a) U. Hasegawa , A. J. Van Der Vlies , E. Simeoni , C. Wandrey , J. A. Hubbell , J. Am. Chem. Soc. 2010, 132, 18273; 21128648 10.1021/ja1075025

[175] a) Z. Jin , Y. Wen , L. Xiong , T. Yang , P. Zhao , L. Tan , T. Wang , Z. Qian , B.‐L. Su , Q. He , Chem. Commun. 2017, 53, 5557; 10.1039/c7cc01576c 28474016

[176] L. Wang , X. Niu , Q. Song , J. Jia , Y. Hao , C. Zheng , K. Ding , H. Xiao , X. Liu , Z. Zhang , J. Control Release 2020, 318, 197. 31672622 10.1016/j.jconrel.2019.10.017

[177] Y. Liu , W. Zhen , Y. Wang , S. Song , H. Zhang , J. Am. Chem. Soc. 2020, 142, 21751. 33337859 10.1021/jacs.0c09482

[178] W.‐D. Oh , Z. Dong , T.‐T. Lim , Appl. Catal., B 2016, 194, 169.

[179] S. S. Lucky , K. C. Soo , Y. Zhang , Chem. Rev. 2015, 115, 1990. 25602130 10.1021/cr5004198

[180] Z. Zhou , J. Song , L. Nie , X. Chen , Chem. Soc. Rev. 2016, 45, 6597. 27722328 10.1039/c6cs00271d PMC5118097

[181] C. A. Robertson , D. H. Evans , H. Abrahamse , J. Photochem. Photobiol. B Biol. 2009, 96, 1. 10.1016/j.jphotobiol.2009.04.001 19406659

[182] A. P. Castano , T. N. Demidova , M. R. Hamblin , Photodiagnosis Photodyn. Ther. 2004, 1, 279. 25048432 10.1016/S1572-1000(05)00007-4 PMC4108220

[183] a) X. Zhao , J. Liu , J. Fan , H. Chao , X. Peng , Chem. Soc. Rev. 2021, 50, 4185; 33527104 10.1039/d0cs00173b

[184] D. Yang , G. Yang , Q. Sun , S. Gai , F. He , Y. Dai , C. Zhong , P. Yang , Adv. Healthcare Mater. 2018, 7, 1800042. 10.1002/adhm.201800042 29527835

[185] C. Yi , Z. Yu , Q. Ren , X. Liu , Y. Wang , X. Sun , S. Yin , J. Pan , X. Huang , Photodiagn. Photodyn. Ther. 2020, 30, 101694. 10.1016/j.pdpdt.2020.101694 32109615

[186] E. Ju , K. Dong , Z. Chen , Z. Liu , C. Liu , Y. Huang , Z. Wang , F. Pu , J. Ren , X. Qu , Angew. Chem., Int. Ed. 2016, 55, 11467. 10.1002/anie.201605509 27504861

[187] D. Guo , J. H. Lei , D. Rong , T. Zhang , B. Zhang , Z. Tang , H. M. Shen , C. X. Deng , S. Qu , Adv. Sci. 2022, 9, 2205106. 10.1002/advs.202205106 PMC9798972 36307905

[188] M. Qiu , D. Wang , H. Huang , T. Yin , W. Bao , B. Zhang , Z. Xie , N. Xie , Z. Wu , C. Ge , Adv. Mater. 2021, 33, 2102562. 10.1002/adma.202102562 34643001

[189] R. Han , M. Zhao , Z. Wang , H. Liu , S. Zhu , L. Huang , Y. Wang , L. Wang , Y. Hong , Y. Sha , ACS Nano 2019, 14, 9532. 31670942 10.1021/acsnano.9b05169

[190] X. Wu , L. Yang , L. Luo , G. Shi , X. Wei , F. Wang , ACS Appl. Bio Mater. 2019, 2, 1998. 10.1021/acsabm.9b00055 35030688

[191] K. Deng , C. Li , S. Huang , B. Xing , D. Jin , Q. Zeng , Z. Hou , J. Lin , Small 2017, 13, 1702299. 10.1002/smll.201702299 28961374

[192] J. G. Croissant , J.‐O. Durand , The Enzymes 2018, 43, 67. 30244809 10.1016/bs.enz.2018.07.004

[193] L. Guo , J. Ge , Q. Liu , Q. Jia , H. Zhang , W. Liu , G. Niu , S. Liu , J. Gong , S. Hackbarth , Adv. Healthcare Mater. 2017, 6, 1601431. 10.1002/adhm.201601431 28338291

[194] Y. Shen , A. J. Shuhendler , D. Ye , J.‐J. Xu , H.‐Y. Chen , Chem. Soc. Rev. 2016, 45, 6725. 27711672 10.1039/c6cs00442c

[195] a) Z. Hou , K. Deng , M. Wang , Y. Liu , M. Chang , S. Huang , C. Li , Y. Wei , Z. Cheng , G. Han , Chem. Mater. 2019, 31, 774;

[196] Y. Chen , H. Xiang , S. Zhuang , Y. Shen , Y. Chen , J. Zhang , Adv. Mater. 2021, 33, 2100129. 10.1002/adma.202100129 34302402

[197] M.‐D. Li , N.‐K. Wong , J. Xiao , R. Zhu , L. Wu , S.‐Y. Dai , F. Chen , G. Huang , L. Xu , X. Bai , J. Am. Chem. Soc. 2018, 140, 15957. 30269478 10.1021/jacs.8b10235

[198] H. Chen , G. D. Wang , Y.‐J. Chuang , Z. Zhen , X. Chen , P. Biddinger , Z. Hao , F. Liu , B. Shen , Z. Pan , Nano Lett. 2015, 15, 2249. 25756781 10.1021/nl504044p PMC5233724

[199] a) L. Ma , X. Zou , B. Bui , W. Chen , K. H. Song , T. Solberg , Appl. Phys. Lett. 2014, 105, 013702;

[200] H. Wang , X. Pan , X. Wang , W. Wang , Z. Huang , K. Gu , S. Liu , F. Zhang , H. Shen , Q. Yuan , ACS Nano 2020, 14, 2847. 31909977 10.1021/acsnano.9b06168

[201] a) Y. Chen , Z. H. Li , P. Pan , J. J. Hu , S. X. Cheng , X. Z. Zhang , Adv. Mater. 2020, 32, 2001452; 10.1002/adma.202001452 32374492

[202] L. He , N. Zheng , Q. Wang , J. Du , S. Wang , Z. Cao , Z. Wang , G. Chen , J. Mu , S. Liu , Adv. Sci. 2023, 10, 2205208. 10.1002/advs.202205208 PMC9811476 36373690

[203] a) M. Guo , H.‐J. Xiang , Y. Wang , Q.‐L. Zhang , L. An , S.‐P. Yang , Y. Ma , Y. Wang , J.‐G. Liu , Chem. Commun. 2017, 53, 3253; 10.1039/c7cc00670e 28261712

[204] H. W. Choi , J. Kim , J. Kim , Y. Kim , H. B. Song , J. H. Kim , K. Kim , W. J. Kim , ACS Nano 2016, 10, 4199. 26953516 10.1021/acsnano.5b07483

[205] H.‐J. Xiang , Q. Deng , L. An , M. Guo , S.‐P. Yang , J.‐G. Liu , Chem. Commun. 2016, 52, 148. 10.1039/c5cc07006f 26503188

[206] L. Wang , Y. Chang , Y. Feng , X. Li , Y. Cheng , H. Jian , X. Ma , R. Zheng , X. Wu , K. Xu , Nano Lett. 2019, 19, 6800. 31466437 10.1021/acs.nanolett.9b01869

[207] a) X. Zhou , Z. Meng , J. She , Y. Zhang , X. Yi , H. Zhou , J. Zhong , Z. Dong , X. Han , M. Chen , Nano‐Micro Lett. 2020, 12, 1; 10.1007/s40820-020-00431-3 PMC7770715 34138094

[208] R. Guo , Y. Tian , Y. Wang , W. Yang , Adv. Funct. Mater. 2017, 27, 1606398.

[209] J. Fan , Q. He , Y. Liu , F. Zhang , X. Yang , Z. Wang , N. Lu , W. Fan , L. Lin , G. Niu , ACS Appl. Mater. Interfaces 2016, 8, 13804. 27213922 10.1021/acsami.6b03737 PMC5233726

[210] a) L. Tan , R. Huang , X. Li , S. Liu , Y.‐M. Shen , Acta Biomater. 2017, 57, 498; 28499633 10.1016/j.actbio.2017.05.019

[211] Y. Wang , X. Huang , Y. Tang , J. Zou , P. Wang , Y. Zhang , W. Si , W. Huang , X. Dong , Chem. Sci. 2018, 9, 8103. 30542560 10.1039/c8sc03386b PMC6238752

[212] a) I. Chakraborty , S. J. Carrington , P. K. Mascharak , Acc. Chem. Res. 2014, 47, 2603; 25003608 10.1021/ar500172f

[213] Q. He , D. O. Kiesewetter , Y. Qu , X. Fu , J. Fan , P. Huang , Y. Liu , G. Zhu , Y. Liu , Z. Qian , Adv. Mater. 2015, 27, 6741. 26401893 10.1002/adma.201502762 PMC4921239

[214] X.‐S. Wang , J.‐Y. Zeng , M.‐J. Li , Q.‐R. Li , F. Gao , X.‐Z. Zhang , ACS Nano 2020, 14, 9848. 32658459 10.1021/acsnano.0c02516

[215] D. W. Zheng , B. Li , C. X. Li , L. Xu , J. X. Fan , Q. Lei , X. Z. Zhang , Adv. Mater. 2017, 29, 1703822. 10.1002/adma.201703822 29024101

[216] X. Q. Wang , F. Gao , X. Z. Zhang , Angew. Chem., Int. Ed. 2017, 56, 9029. 10.1002/anie.201703159 28585742

[217] S. Shen , C. Zhu , D. Huo , M. Yang , J. Xue , Y. Xia , Angew. Chem., Int. Ed. 2017, 56, 8801. 10.1002/anie.201702898 PMC5543408 28464414

[218] R. Xia , X. Zheng , X. Hu , S. Liu , Z. Xie , ACS Appl. Mater. Interfaces 2019, 11, 5782. 30663874 10.1021/acsami.8b18953

[219] L. Feng , S. Gai , Y. Dai , F. He , C. Sun , P. Yang , R. Lv , N. Niu , G. An , J. Lin , Chem. Mater. 2018, 30, 526.

[220] J. Yang , R. Xie , L. Feng , B. Liu , R. Lv , C. Li , S. Gai , F. He , P. Yang , J. Lin , ACS Nano 2019, 13, 13144. 31609581 10.1021/acsnano.9b05985

[221] S. Wu , X. Liu , J. Ren , X. Qu , Small 2019, 15, 1904870. 10.1002/smll.201904870 31750615

[222] X. Li , Y. Liu , F. Fu , M. Cheng , Y. Liu , L. Yu , W. Wang , Y. Wan , Z. Yuan , Nano‐Micro Lett. 2019, 11, 1. 10.1007/s40820-019-0298-5 PMC7770756 34137996

[223] a) L. Zhang , Y. Fan , Z. Yang , M. Yang , C.‐Y. Wong , J. Nanobiotechnol. 2021, 19, 1; 10.1186/s12951-021-01003-2 PMC8420023 34488803

[224] J. Zheng , J. Du , H. Ge , N. Xu , Q. Yao , S. Long , J. Fan , X. Peng , Chem. Eng. J. 2022, 449, 136565.

[225] a) H. Shi , C. Imberti , P. J. Sadler , Inorg. Chem. Front. 2019, 6, 1623; 10.1039/D0QI00685H PMC7610473 33786191

[226] D. Zhou , S. He , Y. Cong , Z. Xie , X. Chen , X. Jing , Y. Huang , J. Mater. Chem. B 2015, 3, 4913. 32262680 10.1039/c5tb00576k

[227] a) Q. Zhang , G. Kuang , D. Zhou , Y. Qi , M. Wang , X. Li , Y. Huang , J. Mater. Chem. B 2020, 8, 5903; 32538396 10.1039/d0tb01103g

[228] Q. Zhang , G. Kuang , S. He , H. Lu , Y. Cheng , D. Zhou , Y. Huang , Nano Lett. 2020, 20, 3039. 32250633 10.1021/acs.nanolett.9b04981

[229] S. Wu , P. Wang , J. Qin , Y. Pei , Y. Wang , Adv. Funct. Mater. 2021, 31, 2102160.

[230] D. Ding , Z. Mei , H. Huang , W. Feng , L. Chen , Y. Chen , J. Zhou , Adv. Sci. 2022, 9, 2200974. 10.1002/advs.202200974 PMC9189647 35488513

[231] R. Song , H. Wang , M. Zhang , Y. Liu , X. Meng , S. Zhai , C. c. Wang , T. Gong , Y. Wu , X. Jiang , Angew. Chem., Int. Ed. 2020, 59, 21032. 10.1002/anie.202007434 32667130

[232] A. Adhikary , A. Kumar , B. J. Palmer , A. D. Todd , M. D. Sevilla , J. Am. Chem. Soc. 2013, 135, 12827. 23885974 10.1021/ja406121x PMC3789529

[233] T. Liu , K. Yang , Z. Liu , Prog. Nat. Sci.: Mater. Int. 2020, 30, 567.

[234] A. C. Begg , F. A. Stewart , C. Vens , Nat. Rev. Cancer 2011, 11, 239. 21430696 10.1038/nrc3007

[235] X. Zhong , X. Wang , G. Zhan , Y. A. Tang , Y. Yao , Z. Dong , L. Hou , H. Zhao , S. Zeng , J. Hu , Nano Lett. 2019, 19, 8234. 31576757 10.1021/acs.nanolett.9b03682

[236] W. Sun , L. Luo , Y. Feng , Y. Cai , Y. Zhuang , R. J. Xie , X. Chen , H. Chen , Angew. Chem., Int. Ed. 2020, 132, 10000. 10.1002/anie.201908712 31418982

[237] a) K. Lu , C. He , N. Guo , C. Chan , K. Ni , G. Lan , H. Tang , C. Pelizzari , Y.‐X. Fu , M. T. Spiotto , Nat. Biomed. Eng. 2018, 2, 600; 31015630 10.1038/s41551-018-0203-4

[238] X. Wang , W. Sun , H. Shi , H. Ma , G. Niu , Y. Li , J. Zhi , X. Yao , Z. Song , L. Chen , Nat. Commun. 2022, 13, 5091. 36042210 10.1038/s41467-022-32054-0 PMC9428140

[239] a) Y. Wang , H. Zhang , Y. Liu , M. H. Younis , W. Cai , W. Bu , Mater. Today 2022, 57, 262; 10.1016/j.mattod.2022.05.022 PMC9681018 36425004

[240] a) L. Chan , P. Gao , W. Zhou , C. Mei , Y. Huang , X.‐F. Yu , P. K. Chu , T. Chen , ACS Nano 2018, 12, 12401; 30407787 10.1021/acsnano.8b06483

[241] X. Wang , Z. Guo , C. Zhang , S. Zhu , L. Li , Z. Gu , Y. Zhao , Adv. Sci. 2020, 7, 1902561. 10.1002/advs.201902561 PMC7080545 32195085

[242] Q. Xu , Y. Yang , J. Lu , Y. Lin , S. Feng , X. Luo , D. Di , S. Wang , Q. Zhao , Coord. Chem. Rev. 2022, 469, 214687.

[243] a) W. Tang , Z. Dong , R. Zhang , X. Yi , K. Yang , M. Jin , C. Yuan , Z. Xiao , Z. Liu , L. Cheng , ACS Nano 2018, 13, 284; 30543399 10.1021/acsnano.8b05982

[244] N. Zheng , S. Zhang , L. Wang , Z. Qi , Q. Peng , L. Jian , Y. Bai , Y. Feng , J. Shen , R. Wang , Nano Res. 2022, 15, 2315.

[245] C. Zhang , X. Wang , X. Dong , L. Mei , X. Wu , Z. Gu , Y. Zhao , Biomaterials 2021, 276, 121023. 34274779 10.1016/j.biomaterials.2021.121023

[246] B. L. Cline , W. Jiang , C. Lee , Z. Cao , X. Yang , S. Zhan , H. Chong , T. Zhang , Z. Han , X. Wu , ACS Nano 2021, 15, 17401. 34694109 10.1021/acsnano.1c01435 PMC9035482

[247] a) S. Liu , W. Li , Y. Zhang , J. Zhou , Y. Du , S. Dong , B. Tian , L. Fang , H. Ding , S. Gai , Nano Lett. 2022, 22, 6409; 35867897 10.1021/acs.nanolett.2c02472

[248] Z. Du , X. Zhang , Z. Guo , J. Xie , X. Dong , S. Zhu , J. Du , Z. Gu , Y. Zhao , Adv. Mater. 2018, 30, 1804046. 10.1002/adma.201804046 30260520

[249] Y. Li , T. Gong , H. Gao , Y. Chen , H. Li , P. Zhao , Y. Jiang , K. Wang , Y. Wu , X. Zheng , Angew. Chem., Int. Ed. 2021, 133, 15600. 10.1002/anie.202103015 33964189

[250] X. Liu , X. Pan , C. Wang , H. Liu , Particuology 2023, 75, 199.

[251] M. Yuan , S. Liang , Y. Zhou , X. Xiao , B. Liu , C. Yang , P. a. Ma , Z. Cheng , J. Lin , Nano Lett. 2021, 21, 6042. 34254814 10.1021/acs.nanolett.1c01220

[252] a) D. Wang , M. Zhang , Y. Zhang , G. Qiu , J. Chen , X. Zhu , C. Kong , X. Lu , X. Liang , L. Duan , Adv. Sci. 2022, 9, 2203106; 10.1002/advs.202203106 PMC9661857 36156442

[253] K. Liu , Z. Jiang , F. Zhao , W. Wang , F. Jäkle , N. Wang , X. Tang , X. Yin , P. Chen , Adv. Mater. 2022, 34, 2206594. 10.1002/adma.202206594 36193773

[254] J. Chen , H. Luo , Y. Liu , W. Zhang , H. Li , T. Luo , K. Zhang , Y. Zhao , J. Liu , ACS Nano 2017, 11, 12849. 29236476 10.1021/acsnano.7b08225

[255] H. Wang , J. Guo , W. Lin , Z. Fu , X. Ji , B. Yu , M. Lu , W. Cui , L. Deng , J. W. Engle , Adv. Mater. 2022, 34, 2110283. 10.1002/adma.202110283 PMC9012683 35179801

[256] S. Yamaguchi , H. Kobayashi , T. Narita , K. Kanehira , S. Sonezaki , N. Kudo , Y. Kubota , S. Terasaka , K. Houkin , Ultrason. Sonochem. 2011, 18, 1197. 21257331 10.1016/j.ultsonch.2010.12.017

[257] a) V. Deepagan , D. G. You , W. Um , H. Ko , S. Kwon , K. Y. Choi , G.‐R. Yi , J. Y. Lee , D. S. Lee , K. Kim , Nano Lett. 2016, 16, 6257; 27643533 10.1021/acs.nanolett.6b02547

[258] C. Dai , S. Zhang , Z. Liu , R. Wu , Y. Chen , ACS Nano 2017, 11, 9467. 28829584 10.1021/acsnano.7b05215

[259] X. Han , J. Huang , X. Jing , D. Yang , H. Lin , Z. Wang , P. Li , Y. Chen , ACS Nano 2018, 12, 4545. 29697960 10.1021/acsnano.8b00899

[260] B. Geng , S. Xu , P. Li , X. Li , F. Fang , D. Pan , L. Shen , Small 2022, 18, 2103528. 10.1002/smll.202103528 34859576

[261] a) F. Gong , L. Cheng , N. Yang , O. Betzer , L. Feng , Q. Zhou , Y. Li , R. Chen , R. Popovtzer , Z. Liu , Adv. Mater. 2019, 31, 1900730; 10.1002/adma.201900730 30977247

[262] S. Liang , B. Liu , X. Xiao , M. Yuan , L. Yang , P. a. Ma , Z. Cheng , J. Lin , Adv. Mater. 2021, 33, 2101467. 10.1002/adma.202101467 34296464

[263] a) Z. Li , T. Zhang , F. Fan , F. Gao , H. Ji , L. Yang , J. Phys. Chem. Lett. 2020, 11, 1228; 31990196 10.1021/acs.jpclett.9b03769

[264] X. Meng , S. Sun , C. Gong , J. Yang , Z. Yang , X. Zhang , H. Dong , ACS Nano 2022, 17, 1174. 10.1021/acsnano.2c08687 36583572

[265] F. Gong , L. Cheng , N. Yang , Y. Gong , Y. Ni , S. Bai , X. Wang , M. Chen , Q. Chen , Z. Liu , Nat. Commun. 2020, 11, 3712. 32709842 10.1038/s41467-020-17485-x PMC7381661

[266] a) X. Cao , Y. Wang , X. Song , W. Lou , X. Li , W. Lu , K. Chen , L. Chen , Y. Chen , B. Huang , Adv. Funct. Mater. 2023, 33, 2300777;

[267] T. Hu , W. Shen , F. Meng , S. Yang , S. Yu , H. Li , Q. Zhang , L. Gu , C. Tan , R. Liang , Adv. Mater. 2023, 35, 2209692. 10.1002/adma.202209692 36780890

[268] L. Sun , Y. Cao , Z. Lu , P. Ding , Z. Wang , F. Ma , Z. Wang , R. Pei , Nano Today 2022, 43, 101434.

[269] X. Lin , Y. Qiu , L. Song , S. Chen , X. Chen , G. Huang , J. Song , X. Chen , H. Yang , Nanoscale Horiz. 2019, 4, 747.

[270] F. Yang , P. Chen , W. He , N. Gu , X. Zhang , K. Fang , Y. Zhang , J. Sun , J. Tong , Small 2010, 6, 1300. 20486225 10.1002/smll.201000173

[271] Z. Jin , Y. Wen , Y. Hu , W. Chen , X. Zheng , W. Guo , T. Wang , Z. Qian , B.‐L. Su , Q. He , Nanoscale 2017, 9, 3637. 28247895 10.1039/c7nr00231a

[272] W. Guo , T. Wang , C. Huang , S. Ning , Q. Guo , W. Zhang , H. Yang , D. Zhu , Q. Huang , H. Qian , Nano Res. 2023, 16, 782.

[273] X. Qiao , J. Liang , L. Qiu , W. Feng , G. Cheng , Y. Chen , H. Ding , Biomaterials 2023, 301, 122252. 37542858 10.1016/j.biomaterials.2023.122252

[274] Z. Lu , L. Zhou , Y. Yao , F. Li , Z. Hong , Y. Wu , X. Li , Adv. Funct. Mater. 2023, 33, 2214749.

[275] T. Gu , Y. Wang , Y. Lu , L. Cheng , L. Feng , H. Zhang , X. Li , G. Han , Z. Liu , Adv. Mater. 2019, 31, 1806803. 10.1002/adma.201806803 30734370

[276] a) Z. Lu , J. Gao , C. Fang , Y. Zhou , X. Li , G. Han , Adv. Sci. 2020, 7, 2001223; 10.1002/advs.202001223 PMC7507307 32995127

[277] a) T. Chen , Y. Fu , R. Zhang , G. Han , X. Li , Biomater. Sci. 2022, 10, 376; 34928270 10.1039/d1bm01464a

[278] J. Zhu , J. Wang , Y. Li , Biomed. Pharmacother. 2023, 159, 114227. 36638597 10.1016/j.biopha.2023.114227

[279] M. Ge , D. Xu , Z. Chen , C. Wei , Y. Zhang , C. Yang , Y. Chen , H. Lin , J. Shi , Nano Lett. 2021, 21, 6764. 34342999 10.1021/acs.nanolett.1c01313

[280] J. A. Rodrigues , J. H. Correia , Cells 2022, 11, 3995.36552759

[281] S. Wang , R. Tian , X. Zhang , G. Cheng , P. Yu , J. Chang , X. Chen , Adv. Mater. 2021, 33, 2007488. 10.1002/adma.202007488 33987898

[282] Q. Wu , N. Xia , D. Long , L. Tan , W. Rao , J. Yu , C. Fu , X. Ren , H. Li , L. Gou , Nano Lett. 2019, 19, 5277. 31331173 10.1021/acs.nanolett.9b01735

[283] T. Tang , X. Xu , Z. Wang , J. Tian , Y. Yang , C. Ou , H. Bao , T. Liu , Chem. Commun. 2019, 55, 13148. 10.1039/c9cc07762f 31617549

[284] X. Chu , K. Li , H. Guo , H. Zheng , S. Shuda , X. Wang , J. Zhang , W. Chen , Y. Zhang , ACS Biomater. Sci. Eng. 2017, 3, 1836. 33429665 10.1021/acsbiomaterials.7b00110

[285] Y. Wang , X. Ren , Y. Zheng , L. Tan , B. Li , C. Fu , Q. Wu , Z. Chen , J. Ren , D. Yang , Small 2023, 19, 2304440. 10.1002/smll.202304440 37544921

[286] S. Li , Z. Chen , L. Tan , Q. Wu , X. Ren , C. Fu , M. Niu , H. Li , X. Meng , Biomaterials 2022, 283, 121472. 35313274 10.1016/j.biomaterials.2022.121472

[287] K. Maier‐Hauff , F. Ulrich , D. Nestler , H. Niehoff , P. Wust , B. Thiesen , H. Orawa , V. Budach , A. Jordan , J. Neurooncol. 2011, 103, 317. 20845061 10.1007/s11060-010-0389-0 PMC3097345

[288] A. R. Rastinehad , H. Anastos , E. Wajswol , J. S. Winoker , J. P. Sfakianos , S. K. Doppalapudi , M. R. Carrick , C. J. Knauer , B. Taouli , S. C. Lewis , Proc. Natl. Acad. Sci. USA 2019, 116, 18590. 31451630 10.1073/pnas.1906929116 PMC6744844

[289] a) S. Bonvalot , P. L. Rutkowski , J. Thariat , S. Carrère , A. Ducassou , M.‐P. Sunyach , P. Agoston , A. Hong , A. Mervoyer , M. Rastrelli , Lancet Oncol. 2019, 20, 1148; 31296491 10.1016/S1470-2045(19)30326-2

[290] S. Di Meo , T. T. Reed , P. Venditti , V. M. Victor , Oxid. Med. Cell. Longev. 2016, 2016, 1245049. 27478531 10.1155/2016/1245049 PMC4960346

[291] a) K. Mortezaee , Cell Biochem. Funct. 2018, 36, 292; 30028028 10.1002/cbf.3351

[292] S. Coso , I. Harrison , C. B. Harrison , A. Vinh , C. G. Sobey , G. R. Drummond , E. D. Williams , S. Selemidis , Antioxid. Redox Signal. 2012, 16, 1229. 22229841 10.1089/ars.2011.4489

[293] a) T. Pecchillo Cimmino , R. Ammendola , F. Cattaneo , G. Esposito , Int. J. Mol. Sci. 2023, 24, 2086; 36768405 10.3390/ijms24032086 PMC9916913

[294] L. Zhu , Y. Zhao , T. Liu , M. Chen , W. P. Qian , B. Jiang , B. G. Barwick , L. Zhang , T. M. Styblo , X. Li , ACS Nano 2022, 16, 18708. 36256454 10.1021/acsnano.2c07440 PMC9764083

[295] W. Zhang , C. Liu , Z. Liu , C. Zhao , J. Zhu , J. Ren , X. Qu , ACS Nano 2022, 16, 20975. 36394517 10.1021/acsnano.2c08604

[296] P. a. Ma , H. Xiao , C. Yu , J. Liu , Z. Cheng , H. Song , X. Zhang , C. Li , J. Wang , Z. Gu , Nano Lett. 2017, 17, 928. 28139118 10.1021/acs.nanolett.6b04269

[297] C. Liu , M. Li , C. Liu , S. Qiu , Y. Bai , L. Fan , W. Tian , J. Mater. Chem. B 2022, 10, 8981. 36300361 10.1039/d2tb01834a

[298] M. Sun , H. Ye , Q. Shi , J. Xie , X. Yu , H. Ling , S. You , Z. He , B. Qin , J. Sun , Adv. Healthcare Mater. 2021, 10, 2100950. 10.1002/adhm.202100950 34541825

[299] Y. He , X. Jin , S. Guo , H. Zhao , Y. Liu , H. Ju , ACS Appl. Mater. Interfaces 2021, 13, 31452. 34197086 10.1021/acsami.1c06613

[300] C. Wu , D. Xu , M. Ge , J. Luo , L. Chen , Z. Chen , Y. You , Y.‐x. Zhu , H. Lin , J. Shi , Nano Today 2022, 46, 101574.

[301] G. Chong , R. Su , J. Gu , Y. Yang , T. Zhang , J. Zang , Y. Zhao , X. Zheng , Y. Liu , S. Ruan , Chem. Eng. J. 2022, 435, 134993.

[302] M. S. Rahman , M. S. Uddin , M. A. Rahman , M. Samsuzzaman , T. Behl , A. Hafeez , A. Perveen , G. E. Barreto , G. M. Ashraf , Curr. Pharm. Des. 2021, 27, 4017. 34126892 10.2174/1381612827666210612051713

[303] a) J. C. Shih , J. B. Wu , K. Chen , J. Neural Transm. 2011, 118, 979; 21359973 10.1007/s00702-010-0562-9 PMC3125068

[304] M. E. Gross , D. B. Agus , T. B. Dorff , J. K. Pinski , D. I. Quinn , O. Castellanos , P. Gilmore , J. C. Shih , Prostate Cancer Prostatic Dis. 2021, 24, 61. 32123315 10.1038/s41391-020-0211-9 PMC7483294

[305] X. Wu , H. Xu , F. Luo , J. Wang , L. Zhao , X. Zhou , Y. Yang , H. Cai , P. Sun , H. Zhou , Biosens. Bioelectron. 2021, 189, 113377. 34090156 10.1016/j.bios.2021.113377

[306] J. D. Hayes , A. T. Dinkova‐Kostova , Trends Biochem. Sci 2014, 39, 199. 24647116 10.1016/j.tibs.2014.02.002

[307] A. T. Dinkova‐Kostova , A. Y. Abramov , Free Radical Biol. Med. 2015, 88, 179. 25975984 10.1016/j.freeradbiomed.2015.04.036 PMC4726722

[308] a) S. Menegon , A. Columbano , S. Giordano , Trends Mol. Med. 2016, 22, 578; 27263465 10.1016/j.molmed.2016.05.002

[309] a) N. Robledinos‐Antón , R. Fernández‐Ginés , G. Manda , A. Cuadrado , Oxid. Med. Cell. Longev. 2019, 2019, 9372182; 31396308 10.1155/2019/9372182 PMC6664516

[310] Z. Sezgin‐Bayindir , S. Losada‐Barreiro , C. Bravo‐Díaz , M. Sova , J. Kristl , L. Saso , Antioxidants 2021, 10, 685. 33925605 10.3390/antiox10050685 PMC8145905

[311] C.‐H. Hsieh , H.‐C. Hsieh , F.‐H. Shih , P.‐W. Wang , L.‐X. Yang , D.‐B. Shieh , Y.‐C. Wang , Theranostics 2021, 11, 7072. 34093872 10.7150/thno.57803 PMC8171079

[312] H. Sun , H. Cai , C. Xu , H. Zhai , F. Lux , Y. Xie , L. Feng , L. Du , Y. Liu , X. Sun , J. Nanobiotechnol. 2022, 20, 1. 10.1186/s12951-022-01654-9 PMC9569109 36242003

[313] a) W. Xiao , J. Loscalzo , Antioxid. Redox Signal. 2020, 32, 1330; 31218894 10.1089/ars.2019.7803 PMC7247050

[314] Y. Liu , Q. Li , L. Zhou , N. Xie , E. C. Nice , H. Zhang , C. Huang , Y. Lei , Oncotarget 2016, 7, 42740. 27057637 10.18632/oncotarget.8600 PMC5173169

[315] a) C. S.‐G. José , L. Quiles , L. Vera‐Ramírez , F. Giampieri , M. D. Navarro‐Hortal , J. Xiao , J. Llopis , M. Battino , A. Varela‐López , Antioxid. Redox Signal. 2020, 33, 860; 32064905 10.1089/ars.2020.8051

[316] a) N. S. Rajasekaran , P. Connell , E. S. Christians , L.‐J. Yan , R. P. Taylor , A. Orosz , X. Q. Zhang , T. J. Stevenson , R. M. Peshock , J. A. Leopold , Cell 2007, 130, 427; 17693254 10.1016/j.cell.2007.06.044 PMC2962423

[317] a) V. R. Martínez , M. V. Aguirre , J. S. Todaro , E. G. Ferrer , P. A. Williams , New J. Chem. 2021, 45, 939;

[318] a) X. Pan , Y. Zhao , T. Cheng , A. Zheng , A. Ge , L. Zang , K. Xu , B. Tang , Chem. Sci. 2019, 10, 8179; 31857884 10.1039/c9sc02020a PMC6836941

[319] a) X. Gao , C. Zhao , K. Wei , B. Hu , Y. Chen , K. Xu , B. Tang , Analyst 2020, 145, 6363; 32985627 10.1039/d0an01011a

[320] X. Gao , K. Wei , B. Hu , K. Xu , B. Tang , Theranostics 2019, 9, 4233. 31281544 10.7150/thno.33783 PMC6592181

[321] X. Zhao , X. Guo , M. Pang , W. Qiu , Z. Luo , Q. Lin , Y. Lu , H. Yin , S. Wang , H. Liu , Nano Res. 2023, 1.

[322] H. Ding , J. Chang , F. He , S. Gai , P. Yang , Adv. Healthcare Mater. 2022, 11, 2101984. 10.1002/adhm.202101984 34788499

[323] a) R. Kumar , R. Banerjee , Crit. Rev. Biochem. Mol. Biol. 2021, 56, 221; 33722121 10.1080/10409238.2021.1893641 PMC8136436

[324] Z. W. Lee , X. Y. Teo , E. W. Tay , C. H. Tan , T. Hagen , P. Moore , L. W. Deng , Br. J. Pharmacol. 2014, 171, 4322. 24827113 10.1111/bph.12773 PMC4241097

[325] a) J. Li , X. Li , Y. Yuan , Q. Wang , L. Xie , Y. Dai , W. Wang , L. Li , X. Lu , Q. Fan , Small 2020, 16, 2002939; 10.1002/smll.202002939 32875678

[326] a) C. Fang , D. Cen , Y. Wang , Y. Wu , X. Cai , X. Li , G. Han , Theranostics 2020, 10, 7671; 32685012 10.7150/thno.45079 PMC7359076

[327] D. Cen , Q. Ge , C. Xie , Q. Zheng , J. Guo , Y. Zhang , Y. Wang , X. Li , Z. Gu , X. Cai , Adv. Mater. 2021, 33, 2104037. 10.1002/adma.202104037 34622500

[328] K. A. Cupp‐Sutton , M. T. Ashby , Antioxidants 2016, 5, 42. 27879667 10.3390/antiox5040042 PMC5187540

[329] a) A. Bhattacharya , M. Seshadri , S. D. Oven , K. Toth , M. M. Vaughan , Y. M. Rustum , Clin. Cancer Res. 2008, 14, 3926; 18559614 10.1158/1078-0432.CCR-08-0212 PMC2504718

[330] a) V. Gandhi , P. P. Phadnis , A. Kunwar , Metallomics 2020, 12, 1253; 32812608 10.1039/d0mt00106f

[331] Z. Chen , Y. Lu , X. Dun , X. Wang , H. Wang , Nutrients 2023, 15, 4189. 37836473 10.3390/nu15194189 PMC10574215

[332] X. Pan , X. Song , C. Wang , T. Cheng , D. Luan , K. Xu , B. Tang , Theranostics 2019, 9, 1794. 31037139 10.7150/thno.31841 PMC6485193

[333] A. Krakowiak , L. Czernek , M. Pichlak , R. Kaczmarek , Int. J. Mol. Sci. 2022, 23, 607. 35054788 10.3390/ijms23020607 PMC8775712

[334] a) T. D. Newton , M. D. Pluth , Chem. Sci. 2019, 10, 10723; 32110352 10.1039/c9sc04616j PMC7006510

[335] S. Peng , H. Wang , Y. Xin , W. Zhao , M. Zhan , J. Li , R. Cai , L. Lu , Nano Today 2021, 40, 101240.

[336] a) I. Ohsawa , M. Ishikawa , K. Takahashi , M. Watanabe , K. Nishimaki , K. Yamagata , K.‐i. Katsura , Y. Katayama , S. Asoh , S. Ohta , Nat. Med. 2007, 13, 688; 17486089 10.1038/nm1577

[337] a) L. Yu , P. Hu , Y. Chen , Adv. Mater. 2018, 30, 1801964; 10.1002/adma.201801964 30066474

[338] G. Zhou , E. Goshi , Q. He , Adv. Healthcare Mater. 2019, 8, 1900463. 10.1002/adhm.201900463 31267691

[339] a) W. Wang , C. Chen , Y. Ying , S. Lv , Y. Wang , X. Zhang , Z. Cai , W. Gu , Z. Li , G. Jiang , ACS Nano 2022, 16, 5597; 35315637 10.1021/acsnano.1c10450

[340] M. Fan , Y. Wen , D. Ye , Z. Jin , P. Zhao , D. Chen , X. Lu , Q. He , Adv. Healthcare Mater. 2019, 8, 1900157. 10.1002/adhm.201900157 30968583

[341] a) G. Yuan , J. Cen , J. Liao , Y. Huang , L. Jie , Nanoscale 2021, 13, 15576; 34524338 10.1039/d1nr03260g

[342] Z. Kou , P. Zhao , Z. Wang , Z. Jin , L. Chen , B.‐L. Su , Q. He , J. Mater. Chem. B 2019, 7, 2759. 32255077 10.1039/c9tb00338j

[343] F. Gong , J. Xu , B. Liu , N. Yang , L. Cheng , P. Huang , C. Wang , Q. Chen , C. Ni , Z. Liu , Chem 2022, 8, 268.

[344] P. Zhao , Z. Jin , Q. Chen , T. Yang , D. Chen , J. Meng , X. Lu , Z. Gu , Q. He , Nat. Commun. 2018, 9, 4241. 30315173 10.1038/s41467-018-06630-2 PMC6185976

[345] N. Yang , F. Gong , B. Liu , Y. Hao , Y. Chao , H. Lei , X. Yang , Y. Gong , X. Wang , Z. Liu , Nat. Commun. 2022, 13, 2336. 35484138 10.1038/s41467-022-29938-6 PMC9051066

[346] H. Zhang , P. Limphong , J. Pieper , Q. Liu , C. K. Rodesch , E. Christians , I. J. Benjamin , The FASEB J. 2012, 26, 1442. 22202674 10.1096/fj.11-199869 PMC3316899

[347] M. Wang , R. J. Kaufman , Nat. Rev. Cancer 2014, 14, 581. 25145482 10.1038/nrc3800

[348] L. Chen , X. Jiang , M. Lv , X. Wang , P. Zhao , M. Zhang , G. Lv , J. Wu , Y. Liu , Y. Yang , Chem 2022, 8, 866.

[349] X. Cao , L. Wu , J. Zhang , M. Dolg , J. Comput. Chem. 2020, 41, 305. 31713255 10.1002/jcc.26103

[350] a) W. Xiao , R.‐S. Wang , D. E. Handy , J. Loscalzo , Antioxid. Redox Signal. 2018, 28, 251; 28648096 10.1089/ars.2017.7216 PMC5737637

[351] D. Trachootham , W. Khoonin , Cancer Commun. 2019, 39, 1. 10.1186/s40880-019-0355-y PMC6416897 30867065

[352] a) Z. Li , B. W. Ji , P. D. Dixit , K. Tchourine , E. C. Lien , A. M. Hosios , K. L. Abbott , J. C. Rutter , A. M. Westermark , E. F. Gorodetsky , Nat. Metab. 2022, 4, 711; 35739397 10.1038/s42255-022-00588-8 PMC10305743

[353] S. Zhang , H. Qin , S. Cheng , Y. Zhang , N. Gao , M. Zhang , Angew. Chem., Int. Ed. 2023, 62, e202300083. 10.1002/anie.202300083 36807970

[354] X. Cheng , H. Li , X. Ge , L. Chen , Y. Liu , W. Mao , B. Zhao , W.‐E. Yuan , Front. Mol. Biosci. 2020, 7, 576420. 33330618 10.3389/fmolb.2020.576420 PMC7729065

[355] a) H. Min , J. Wang , Y. Qi , Y. Zhang , X. Han , Y. Xu , J. Xu , Y. Li , L. Chen , K. Cheng , Adv. Mater. 2019, 31, 1808200; 10.1002/adma.201808200 30773718

[356] Y. Xiong , C. Xiao , Z. Li , X. Yang , Chem. Soc. Rev. 2021, 50, 6013. 34027953 10.1039/d0cs00718h

[357] H. Fan , G. Yan , Z. Zhao , X. Hu , W. Zhang , H. Liu , X. Fu , T. Fu , X. B. Zhang , W. Tan , Angew. Chem., Int. Ed. 2016, 55, 5477. 10.1002/anie.201510748 PMC4971833 27010667

[358] J. Liu , Y. Yuan , Y. Cheng , D. Fu , Z. Chen , Y. Wang , L. Zhang , C. Yao , L. Shi , M. Li , J. Am. Chem. Soc. 2022, 144, 4799. 35192770 10.1021/jacs.1c11856

[359] L. An , X. Wang , X. Rui , J. Lin , H. Yang , Q. Tian , C. Tao , S. Yang , Angew. Chem., Int. Ed. 2018, 57, 15782. 10.1002/anie.201810082 30307092

[360] L. Kou , R. Sun , S. Xiao , Y. Zheng , Z. Chen , A. Cai , H. Zheng , Q. Yao , V. Ganapathy , R. Chen , ACS Appl. Mater. Interfaces 2019, 11, 26722. 31276364 10.1021/acsami.9b09784

[361] H. Zhong , P. Y. Huang , P. Yan , P. L. Chen , Q. Y. Shi , Z. A. Zhao , J. X. Chen , X. Shu , P. Wang , B. Yang , Adv. Healthcare Mater. 2021, 10, 2100770.

[362] P. K. Mandal , A. Seiler , T. Perisic , P. Kölle , A. B. Canak , H. Förster , N. Weiss , E. Kremmer , M. W. Lieberman , S. Bannai , J. Biol. Chem. 2010, 285, 22244. 20463017 10.1074/jbc.M110.121327 PMC2903358

[363] S. L. Cramer , A. Saha , J. Liu , S. Tadi , S. Tiziani , W. Yan , K. Triplett , C. Lamb , S. E. Alters , S. Rowlinson , Nat. Med. 2017, 23, 120. 27869804 10.1038/nm.4232 PMC5218918

[364] Z.‐H. Li , Y. Chen , X. Zeng , X.‐Z. Zhang , Nano Today 2021, 38, 101150.

[365] L. Cai , S. Zhou , B. Yu , E. Zhou , Y. Zheng , N. S. I. Ahmed , X. Xu , Y. Wang , Z. Cai , L. Zhang , Chem. Eng. J. 2022, 446, 137110.

[366] B. Niu , K. Liao , Y. Zhou , T. Wen , G. Quan , X. Pan , C. Wu , Biomaterials 2021, 277, 121110. 34482088 10.1016/j.biomaterials.2021.121110

[367] K. Zhang , X. Meng , Z. Yang , H. Dong , X. Zhang , Biomaterials 2020, 258, 120278. 32781328 10.1016/j.biomaterials.2020.120278

[368] Y. Yang , Y. Yu , H. Chen , X. Meng , W. Ma , M. Yu , Z. Li , C. Li , H. Liu , X. Zhang , ACS Nano 2020, 14, 13536. 32924505 10.1021/acsnano.0c05541

[369] a) F. Gao , X. Yang , X. Luo , X. Xue , C. Qian , M. Sun , Adv. Funct. Mater. 2020, 30, 2001546;

[370] S. Jiang , Q. He , C. Li , K. Dang , L. Ye , W. Zhang , Y. Tian , Sci. China Mater. 2022, 65, 1112.

[371] Y. Su , Z. Zhang , L. T. O. Lee , L. Peng , L. Lu , X. He , X. Zhang , Adv. Healthcare Mater. 2023, 12, 2202663. 10.1002/adhm.202202663 36653312

[372] S. Huang , H. Le , G. Hong , G. Chen , F. Zhang , L. Lu , X. Zhang , Y. Qiu , Z. Wang , Q. Zhang , Acta Biomater. 2022, 148, 244. 35709941 10.1016/j.actbio.2022.06.017

[373] W. Bechtel , G. Bauer , Anticancer Res. 2009, 29, 4541. 20032403

[374] C. Xie , D. Cen , Z. Ren , Y. Wang , Y. Wu , X. Li , G. Han , X. Cai , Adv. Sci. 2020, 7, 1903512. 10.1002/advs.201903512 PMC7141047 32274323

[375] L.‐H. Fu , Z.‐Z. Wei , K.‐D. Hu , L.‐Y. Hu , Y.‐H. Li , X.‐Y. Chen , Z. Han , G.‐F. Yao , H. Zhang , J. Microbiol. 2018, 56, 238. 29492867 10.1007/s12275-018-7537-1

[376] a) C. Xie , D. Cen , Z. Ren , Y. Wang , Y. Wu , X. Li , G. Han , X. Cai , Adv. Sci. 2020, 7, 1903512; 10.1002/advs.201903512 PMC7141047 32274323

[377] B. Zhao , Y. Wang , X. Yao , D. Chen , M. Fan , Z. Jin , Q. He , Nat. Commun. 2021, 12, 1345. 33649319 10.1038/s41467-021-21618-1 PMC7921091

[378] H. Ge , J. Du , S. Long , X. Xia , J. Zheng , N. Xu , Q. Yao , J. Fan , X. Peng , Adv. Healthcare Mater. 2022, 11, 2101449. 10.1002/adhm.202101449 34879433

[379] I. de Lázaro , D. J. Mooney , Nat. Mater. 2021, 20, 1469. 34226688 10.1038/s41563-021-01047-7

[380] A. Metere , C. E. Graves , M. Chirico , M. J. Caramujo , M. E. Pisanu , E. Iorio , Biology 2020, 9, 112. 32471147 10.3390/biology9060112 PMC7345942

[381] H. J. Forman , H. Zhang , Nat. Rev. Drug Discovery 2021, 20, 689. 34194012 10.1038/s41573-021-00233-1 PMC8243062

[382] a) T. C. Jorgenson , W. Zhong , T. D. Oberley , Cancer Res. 2013, 73, 6118; 23878188 10.1158/0008-5472.CAN-13-1117 PMC3800221

[383] Y. Li , P. Zhao , T. Gong , H. Wang , X. Jiang , H. Cheng , Y. Liu , Y. Wu , W. Bu , Angew. Chem., Int. Ed. 2020, 59, 22537. 10.1002/anie.202003653 32856362

[384] M. Wang , M. Chang , C. Li , Q. Chen , Z. Hou , B. Xing , J. Lin , Adv. Mater. 2022, 34, 2106010. 10.1002/adma.202106010 34699627

[385] Y. Xu , S. Y. Liu , L. Zeng , H. Ma , Y. Zhang , H. Yang , Y. Liu , S. Fang , J. Zhao , Y. Xu , Adv. Mater. 2022, 34, 2204733. 10.1002/adma.202204733 36054475

[386] Y. Huang , S. Wu , L. Zhang , Q. Deng , J. Ren , X. Qu , ACS Nano 2022, 16, 4228. 35213138 10.1021/acsnano.1c10231

[387] Y. Tian , W. Yi , Q. Shao , M. Ma , L. Bai , R. Song , P. Zhang , J. Si , X. Hou , J. Fan , Chem. Eng. J. 2023, 462, 142156.

[388] L. Hou , F. Gong , Z. Han , Y. Wang , Y. Yang , S. Cheng , N. Yang , Z. Liu , L. Cheng , Angew. Chem., Int. Ed. 2022, 61, e202208849. 10.1002/anie.202208849 35929496

[389] Z. Huang , Y. Wang , D. Yao , J. Wu , Y. Hu , A. Yuan , Nat. Commun. 2021, 12, 145. 33420008 10.1038/s41467-020-20243-8 PMC7794559

[390] L. Liu , Q. Li , L. Chen , L. Song , X. Zhang , H. Huo , Z. You , Y. Wu , Z. Wu , J. Ye , Chem. Sci. 2022, 13, 12840. 36519050 10.1039/d2sc03036e PMC9645394

[391] M. Chang , Z. Hou , M. Wang , C. Yang , R. Wang , F. Li , D. Liu , T. Peng , C. Li , J. Lin , Angew. Chem., Int. Ed. 2021, 60, 12971. 10.1002/anie.202101924 33772996

[392] B. Geng , J. Hu , Y. Li , S. Feng , D. Pan , L. Feng , L. Shen , Nat. Commun. 2022, 13, 5735. 36175446 10.1038/s41467-022-33474-8 PMC9523047

[393] T. Nie , W. Zou , Z. Meng , L. Wang , T. Ying , X. Cai , J. Wu , Y. Zheng , B. Hu , Adv. Mater. 2022, 34, 2206286. 10.1002/adma.202206286 36134532

[394] L. Zhu , Y. You , M. Zhu , Y. Song , J. Zhang , J. Hu , X. Xu , X. Xu , Y. Du , J. Ji , Adv. Mater. 2022, 34, 2207174. 10.1002/adma.202207174 36210735

[395] C. Wu , Z. Liu , Z. Chen , D. Xu , L. Chen , H. Lin , J. Shi , Sci. Adv. 2021, 7, eabj8833. 34550744 10.1126/sciadv.abj8833 PMC8457667

[396] C. Li , J. Ye , X. Yang , S. Liu , Z. Zhang , J. Wang , K. Zhang , J. Xu , Y. Fu , P. Yang , ACS Nano 2022, 16, 18143. 36260703 10.1021/acsnano.2c05152

[397] L. Feng , R. Zhao , L. Yang , B. Liu , S. Dong , C. Qian , J. Liu , Y. Zhao , ACS Nano 2023, 17, 1622. 10.1021/acsnano.2c11470 36623255

[398] X. Fan , B. Chen , H. Xu , A. Pan , S. Liang , S. Tan , Y. He , Chem. Mater. 2023, 35, 3124.

[399] D. Liu , M. Liu , Y. Wan , X. Zhou , S. Yang , L. An , G. Huang , Q. Tian , Chem. Eng. J. 2021, 422, 130098.

[400] Y. Zhang , J. Fang , S. Ye , Y. Zhao , A. Wang , Q. Mao , C. Cui , Y. Feng , J. Li , S. Li , Nat. Commun. 2022, 13, 1685. 35354794 10.1038/s41467-022-29284-7 PMC8967875

[401] J. Chen , S. Lin , D. Zhao , L. Guan , Y. Hu , Y. Wang , K. Lin , Y. Zhu , Adv. Funct. Mater. 2021, 31, 2006853.

